# Differences in Outcomes and Factors Associated With Mortality Among Patients With SARS-CoV-2 Infection and Cancer Compared With Those Without Cancer: A Systematic Review and Meta-analysis

**DOI:** 10.1001/jamanetworkopen.2022.10880

**Published:** 2022-05-09

**Authors:** Emma Khoury, Sarah Nevitt, William Rohde Madsen, Lance Turtle, Gerry Davies, Carlo Palmieri

**Affiliations:** 1 Department of Molecular and Clinical Cancer Medicine, University of Liverpool, Institute of Translational Medicine, Liverpool, United Kingdom; 2 University of Liverpool, School of Medicine, Liverpool, United Kingdom; 3 Department of Health Data Science, Institute of Population Health, University of Liverpool, United Kingdom; 4 Department of Political Science and School of Public Policy, University College London, London, United Kingdom; 5 Department of Political Science, University of Copenhagen, Copenhagen, Denmark; 6 Tropical and Infectious Disease Unit, Liverpool University Hospitals National Health Service (NHS) Foundation Trust, Member of Liverpool Health Partners, Liverpool, United Kingdom; 7 Department of Clinical Infection Microbiology and Immunology, Department of Clinical Infection, University of Liverpool, Liverpool, United Kingdom; 8 University of Liverpool Institute of Infection and Global Health, Veterinary and Ecological Sciences, Liverpool, United Kingdom; 9 The Clatterbridge Cancer Centre NHS Foundation Trust, Liverpool, United Kingdom

## Abstract

**Importance:**

SARS-CoV-2 infection has been associated with more severe disease and death in patients with cancer. However, the implications of certain tumor types, treatments, and the age and sex of patients with cancer for the outcomes of COVID-19 remain unclear.

**Objective:**

To assess the differences in clinical outcomes between patients with cancer and SARS-CoV-2 infection and patients without cancer but with SARS-CoV-2 infection, and to identify patients with cancer at particularly high risk for a poor outcome.

**Data Sources:**

PubMed, Web of Science, and Scopus databases were searched for articles published in English until June 14, 2021. References in these articles were reviewed for additional studies.

**Study Selection:**

All case-control or cohort studies were included that involved 10 or more patients with malignant disease and SARS-CoV-2 infection with or without a control group (defined as patients without cancer but with SARS-CoV-2 infection). Studies were excluded if they involved fewer than 10 patients, were conference papers or abstracts, were preprint reports, had no full text, or had data that could not be obtained from the corresponding author.

**Data Extraction and Synthesis:**

Two investigators independently performed data extraction using the Preferred Reporting Items for Systematic Reviews and Meta-analyses (PRISMA) reporting guideline. Meta-analysis was performed using a random-effects model.

**Main Outcomes and Measures:**

The difference in mortality between patients with cancer and SARS-CoV-2 infection and control patients as well as the difference in outcomes for various tumor types and cancer treatments. Pooled case fatality rates, a random-effects model, and random-effects meta-regressions were used.

**Results:**

A total of 81 studies were included, involving 61 532 patients with cancer. Among 58 849 patients with available data, 30 557 male individuals (52%) were included and median age ranged from 35 to 74 years. The relative risk (RR) of mortality from COVID-19 among patients with vs without cancer when age and sex were matched was 1.69 (95% CI, 1.46-1.95; *P* < .001; *I*^2^ = 51.0%). The RR of mortality in patients with cancer vs control patients was associated with decreasing age (exp [b], 0.96; 95% CI, 0.92-0.99; *P* = .03). Compared with other cancers, lung cancer (RR, 1.68; 95% CI, 1.45-1.94; *P* < .001; *I*^2^ = 32.9%), and hematologic cancer (RR, 1.42; 95% CI, 1.31-1.54; *P* < .001; *I*^2^ = 6.8%) were associated with a higher risk of death. Although a higher point estimate was found for genitourinary cancer (RR, 1.11; 95% CI, 1.00-1.24; P = .06; I2 = 21.5%), the finding was not statistically significant. Breast cancer (RR, 0.51; 95% CI, 0.36-0.71; *P* < .001; *I*^2^ = 86.2%) and gynecological cancer (RR, 0.76; 95% CI, 0.62-0.93; *P* = .009; *I*^2^ = 0%) were associated with a lower risk of death. Chemotherapy was associated with the highest overall pooled case fatality rate of 30% (95% CI, 25%-36%; *I*^2^ = 86.97%; range, 10%-100%), and endocrine therapy was associated with the lowest at 11% (95% CI, 6%-16%; *I*^2^ = 70.68%; range, 0%-27%).

**Conclusions and Relevance:**

Results of this study suggest that patients with cancer and SARS-CoV-2 infection had a higher risk of death than patients without cancer. Younger age, lung cancer, and hematologic cancer were also risk factors associated with poor outcomes from COVID-19.

## Key Points

**Question:**

What are the clinical outcomes for patients with both cancer and SARS-CoV-2 infection?

**Findings:**

In this systematic review and meta-analysis of 81 studies involving 61 532 patients with cancer, patients who were younger, had lung cancer, or had hematologic cancer were at an increased risk of mortality from COVID-19. Among anticancer treatments, chemotherapy was associated with the highest mortality risk and endocrine therapy was associated with the lowest risk.

**Meaning:**

Findings of this study suggest that younger patients with cancer are a high-risk population for poor outcomes from COVID-19.

## Teaser

This systematic review and meta-analysis analyzed the association of age, sex, and cancer types and therapies with survival rates among patients with both cancer and COVID-19 diagnosis.

## Introduction

Individuals with cancer are prone to respiratory viruses because of immunosuppression from either the underlying disease or therapy. This susceptibility has been demonstrated with influenza, which is associated with an increased mortality rate in patients with solid and hematologic cancer.^1,2^ Furthermore, rhinovirus, if present before hematopoietic cell transplant, is associated with a substantial increase in mortality.^3^ With the emergence of the SARS-CoV-2 pandemic, there has been an intense global effort to understand the impact of infection with SARS-CoV-2 and the outcomes of COVID-19 for patients with cancer.

Understanding the possible risks, consequences, and complications of SARS-CoV-2 infection is important for patients, family, and health care systems. For patients and their families, such information enables informed decisions involving the risks of undergoing anticancer treatment during the pandemic and the degree to which they should limit social and familial interactions. For health care systems, these data are vital for informing decisions regarding treatment risk, protecting patients with cancer, prioritizing a heterogeneous population by cancer type and treatment, implementing preventive measures, and providing antiviral treatments. Such information is also vital in planning the response to future pandemics.

The first large population-level data on outcomes of patients with COVID-19 were released by the International Severe Acute Respiratory and Emerging Infections Consortium World Health Organization Clinical Characterization Protocol UK.^4^ The data revealed that 10% of the 20 133 patients who were hospitalized with COVID-19 had a history of malignant neoplasm.^4^ A significant increase in hospital mortality was reported in those patients (hazard ratio [HR], 1.13; 95% CI, 1.02-1.24; *P* = .02).^4^ Meanwhile, population-level data from the Intensive Care National Audit and Research Centre indicated that a lower proportion of patients with cancer were admitted to the intensive care unit compared with patients with other viral pneumonias (non–COVID-19) (2.5% vs 5.8%).^5^ If admitted to critical care, individuals with immunosuppressed systems had an increased likelihood of death.^6^

Numerous cancer-specific studies have been undertaken as part of the effort to understand the consequences of COVID-19 in patients with cancer. These studies found that patients with cancer and SARS-CoV-2 infection have a more severe disease course, with older patients with cancer^7,8,9,10,11,12,13,14,15,16,17,18,19,20,21,22,23,24,25,26,27^ and those with hematologic cancer reported to be at a particularly high risk.^7,8,11,23,28,29,30,31,32,33^ However, many of these studies reported disparate results, particularly regarding the association of outcomes with cancer type and recent cancer treatment.^10,15,23,34,35^ These studies varied in size and nature and were limited by a lack of or small comparator groups of patients without cancer^12,14,16,18,19,21,23,26,29,36,37,38,39,40,41,42,43,44,45^ as well as by selectivity in which tumor types or cancer therapies were included in their analysis.^9,11,18,23,25,29,32,40,43,46,47,48,49,50,51,52,53,54,55,56,57,58,59^ The lack of a contemporaneous age- and sex-matched population without cancer, variability in data collection and reporting, and variation in follow-up times also limited the published cohort studies. In some studies that examined a cohort without cancer, historical patients or registry data were used.^36^ Given all of these factors, confounding biases may be present because of unmeasured confounders.

In the current systematic review and meta-analysis of the available published data, we aimed to assess the differences in clinical outcomes between patients with cancer and SARS-CoV-2 infection and patients without cancer but with SARS-CoV-2 infection, and to identify patients with cancer at particularly high risk for a poor outcome. Such research and information, we believe, will further the understanding of the implications of the SARS-CoV-2 pandemic and the possible novel risk factors for poor outcome in this patient population that have not been identified in previous cohort studies.

## Methods

### Search Strategy and Literature Search

We conducted a systematic review and meta-analysis of the published literature and followed the Preferred Reporting Items for Systematic Reviews and Meta-analyses (PRISMA) reporting guideline.^60^ Repeated searches of PubMed, Web of Science, and Scopus databases were performed for articles that were published until June 14, 2021. References in these articles were also reviewed to check for other relevant studies, with duplicate publications identified and removed. Search strategies and results from the literature search are shown in eTables 1 to 3 in the Supplement.

### Study Selection

Two of us (E.K. and C.P.) independently screened all titles and abstracts after the initial de-duplication. The inclusion criteria were (1) any case-control or cohort study with or without a control group (defined as patients without cancer but with SARS-CoV-2 infection) that (2) was published in English as a full-text article and (3) involved patients with cancer and confirmed or suspected SARS-CoV-2 infection or COVID-19 and (4) described 1 or more of their incidences, presentations, management, or outcomes.

We excluded research with fewer than 10 patients, conference papers or abstracts, preprint reports, articles with full text that could not be extracted, studies with data that could not be obtained from the corresponding author, and animal studies. Among studies that reported overlapping data sets, we selected those with the largest and most up-to-date cohorts. Discrepancies were resolved by consensus.

### Data Extraction and Quality Assessment

Two of us (E.K. and C.P.) independently extracted the following data: first author, study type, period of data collection, country of data collection, number of male and female patients, median or mean age, cancer treatment intervals before COVID-19 diagnosis or hospitalization, unadjusted and adjusted odds ratios or HRs for severe disease and death for each cancer type and for each cancer treatment type, and the number of patients with cancer and SARS-CoV-2 infection and their cancer type. Study-level data on race and ethnicity are provided in eResults in the Supplement. Insufficient data on race and ethnicity were available and thus were not incorporated into this meta-analysis.

The quality of the included studies was assessed using the Newcastle-Ottawa scale for case-control and cohort studies (eFigure 1 in the Supplement).^61^ Publication bias across studies was assessed through visual inspection of a funnel plot for asymmetry (eFigure 2 in the Supplement).

### Outcomes and Statistical Analysis

The main outcome of interest was the difference in mortality. We performed a meta-analysis to compare mortality in patients with cancer and SARS-CoV-2 infection vs control patients and in patients with cancer and a tumor type vs patients with cancer without that tumor type. Results of this meta-analysis were presented as pooled risk ratios (RRs) with 95% CIs.

We also performed meta-analyses to pool case fatality rates by tumor type and by type of cancer treatment among patients with cancer. Results of these meta-analyses were presented as pooled proportions and 95% CIs.

Clinical heterogeneity was assessed by examining study design, patient characteristics, outcome definitions, and study quality in all included studies. Any important differences between studies with regard to design, patient population, outcome definitions, and quality were described. Between-study statistical heterogeneity was quantified according to random-effects heterogeneity parameter tau, and *I*^2^ statistics (defined as the percentage of the variability in effect estimates owing to statistical heterogeneity rather than sampling error) were calculated for all meta-analyses.

Given that statistical heterogeneity was anticipated owing to the expected variability in study design and participant characteristics, all meta-analyses were conducted using a random-effects model, with restricted maximum likelihood to estimate between-studies heterogeneity. Statistical heterogeneity was quantified using the *I*^2^ statistic. To examine the implications of age and sex for mortality among patients with cancer and control patients, random-effects meta-regressions were conducted.

Meta-analyses and meta-regressions were performed with the admetan,^62^ metaprop,^63^ and metareg^64^ commands in Stata, version 14.1 (StataCorp LLC). Additional figures of study characteristics were produced in R, version 4.0.4 (R Foundation for Statistical Computing) (eFigures 4 to 7, 10, 12, 14, and 16 in the Supplement). A *z* test was used to compare 2 independent groups. A 2-sided *P* < .05 indicated statistical significance.

## Results

The initial search retrieved 1150 articles for review (eFigure 3 in the Supplement). After the inclusion of records that were identified through additional sources and the removal of duplicate articles, 1004 records were screened. We obtained 215 articles to assess for eligibility. Of these, 134 were excluded (eFigure 3 in the Supplement). A total of 81 studies were included in this systematic review and meta-analysis.

### Global Distribution of Studies

The 81 studies involved 61 532 patients with cancer and SARS-CoV-2 infection (eFigure 4 and eTable 4 in the Supplement) and consisted of 61 retrospective^7,8,10,11,12,13,14,16,17,18,19,20,21,22,23,24,25,29,30,31,32,34,36,37,38,39,40,41,42,43,44,45,48,49,53,54,55,57,58,59,65,66,67,68,69,70,71,72,73,74,75,76,77,78,79,80,81,82,83,84,85^; 17 prospective^4,26,27,28,33,35,46,50,51,52,56,86,87,88,89,90,91^; and 3 retroprospective^9,15,47^ (wherein data were collected on both patients who were eligible before study commencement and those who entered after study commencement) studies. The studies originated from 28 countries and 5 continents (eFigure 5, eFigure 6, and eTable 5 in the Supplement). Eighty studies provided recruitment information by country, and the following 5 countries had the highest numbers of recruited patients: US, UK, Italy, France, and China.

### Patient Characteristics and Cancer Types

Ten of 81 studies (12%) had cohorts that included both patients with cancer and SARS-CoV-2 infection and control patients^4,65,72,75,79,80,84,86,89,91^; however, most studies (n = 71 [88%]) reported on cohorts of patients with cancer only. The number of patients with cancer in the 81 articles ranged from 11 to 38 614. Where data were available, the cohorts comprised 52% male (n = 30 557 of 58 849) and 48% female (n = 28 269 of 58 849) patients, with a median age ranging from 35 to 74 years. The most frequently reported comorbid conditions were hypertension, diabetes, cardiovascular disease, and pulmonary disease (eFigures 7 to 10 in the Supplement). Most patients (34 117 [55%]) were hospitalized, and the rest of the patients were outpatients or unknown (eFigure 11 and eTable 6 in the Supplement).

Most studies (55 of 81 [68%]) included patients with both solid and hematologic cancer (n = 55 668),^4,7,8,10,12,13,14,15,16,19,20,21,22,24,26,27,28,30,31,33,34,35,36,37,38,39,41,42,44,45,65,66,68,69,70,71,72,73,74,75,76,78,79,80,81,82,83,84,85,86,87,88,89,90,91^ and 18 studies (22%) focused on patients with hematologic cancer alone (n = 2526).^11,17,18,23,25,29,32,40,43,50,51,53,54,56,58,59,67,77^ Of these 18 studies, 4 reported exclusively on multiple myeloma^18,50,53,59^ and 2 on chronic lymphocytic leukemia.^17,67^ Eight studies (10%) focused on patients with solid malignant neoplasms (n = 3338),^9,46,47,48,49,52,55,57^ of which 2 reported on thoracic cancers,^46,49^ 2 on gynecological cancers,^48,57^ and 2 on breast cancer.^52,55^

Tumor type was reported in 68 of 81 studies (84%) involving 43 676 patients (eTable 7 in the Supplement).^7,8,9,10,11,12,13,14,15,16,17,18,19,20,21,22,23,24,25,26,27,28,29,30,31,32,33,34,35,36,37,38,39,40,42,43,44,45,46,47,48,49,50,51,52,53,54,55,56,57,58,59,66,67,68,69,70,73,74,76,77,78,81,82,83,85,87,90^ The most frequent cancer types were hematologic cancer, representing 9672 patients, followed by breast (8322 patients), genitourinary (7624 patients), skin and melanoma (6613 patients), gastrointestinal (4124 patients), and thoracic (2104 patients) cancers. For 1139 patients, the tumor type was documented as other or unknown (eFigures 10 and 12 in the Supplement).

### Presenting Symptoms of Infection and Radiological Findings

Fifty-three of 81 studies (65%) reported presenting symptoms in patients with cancer and SARS-CoV-2 infection, with fever and cough being the most commonly reported symptoms (eFigure 13 in the Supplement).^9,10,11,13,14,15,17,19,20,21,23,24,25,27,28,31,32,33,34,35,37,41,42,43,45,46,48,49,50,52,53,54,55,56,57,58,66,67,68,69,70,74,76,77,78,81,82,83,84,85,87,88,90^ In addition, 27 studies (33%) reported on radiological findings in patients with cancer and SARS-CoV-2 infection (eTable 8 in the Supplement).^10,13,14,17,25,31,32,33,35,39,42,43,45,50,52,54,55,56,57,58,66,69,78,81,84,85,87^ Both symptoms and radiological findings are summarized in the eResults in the Supplement.

### Mortality of Patients With Cancer vs Control Patients

Nineteen of 81 studies (24%) compared patients with cancer (n = 3926) and SARS-CoV-2 infection with control patients (n = 38 847).^12,14,16,18,19,21,23,26,29,36,37,38,39,40,41,42,43,44,45^ The details of these 19 studies, including the matching of the 2 cohorts and the nature of the control patients, are described in the Table. No obvious asymmetry across these studies was observed when assessing for publication bias (eFigure 2 in the Supplement).

**Table.** Outcome of Patients With or Without Hematologic Cancer in 19 Studies

Source	Comparison^a^	No.				Features
		Patients with cancer and SARS-CoV-2 infection (n = 3926)	Deaths (n = 774)	Control patients (n = 38 847)^a^	Deaths (n = 2594)	
Yigenoglu et al,^29^ 2021^b^	Control patients matched by age, sex, and comorbidities	740	102	740	50	Patient characteristics and outcomes
Johannesen et al,^36^ 2021	Control patients	547	56	7841	158	Patient characteristics and outcomes
Rüthrich et al,^37^ 2021	Control patients matched by age	435	97	2636	367	Patient characteristics and outcomes
Montopoli et al,^38^ 2020	Control patients	430	75	4532	313	Patient characteristics and outcomes
Miyashita et al,^12^ 2020	Control patients matched by age	334	37	5354	518	Patient outcomes
Lunski et al,^44^ 2021	Control patients (Ochsner Health System)	157	56	1460	372	Patient characteristics, laboratory markers, and outcomes
Tian et al,^14^ 2020	Control patients matched 1:2 by propensity score	232	46	519	56	Patient characteristics, laboratory markers, and outcomes
Mehta et al,^16^ 2020	Control patients matched 1:5 by propensity score, age, and sex	218	61	1090	149	Patient characteristics and outcomes
Martínez-López et al,^18^ 2020^b^	Control patients matched by age and sex	167	56	167	38	Patient characteristics, laboratory markers, and outcomes
Brar et al,^19^ 2020	Control patients matched 1:4 by age, sex, and comorbidities	117	29	468	100	Patient characteristics and outcomes
Meng et al,^39^ 2020	Control patients matched 1:3 by propensity score	109	32	327	40	Patient characteristics, laboratory markers, and outcomes
Dai et al,^21^ 2020	Control patients matched by age	105	12	536	21	Patient characteristics and outcomes
Cattaneo et al,^40^ 2020^b^	Control patients matched by age, sex, comorbidities, and respiratory failure	102	40	102	24	Patient characteristics and outcomes
Shah et al,^23^ 2020^b^	Control patients matched by age and sex	80	31	1115	223	Patient characteristics and outcomes
Sun et al,^41^ 2021	Control patients	67	9	356	4	Patient characteristics and outcomes
Joharatnam-Hogan et al,^45^ 2020	Control patients matched by age, sex, and comorbidities	30	11	90	32	Patient characteristics, laboratory markers, and outcomes
Stroppa et al,^42^ 2020	Control patients matched by age, sex, pneumonia, and antiviral treatment	25	9	31	5	Patient characteristics and outcomes
Liang et al,^26^ 2020	Control patients	18	7	1572	124	Patient characteristics and outcomes
He et al,^43^ 2020^b^	Health care workers without cancer but with COVID-19	13	8	11	0	Patient characteristics, laboratory markers, and outcomes

^a^ Control patients were defined as patients without cancer but with SARS-CoV-2 infection.

^b^ Study that examined patients with hematologic cancer.

**Figure 1.**
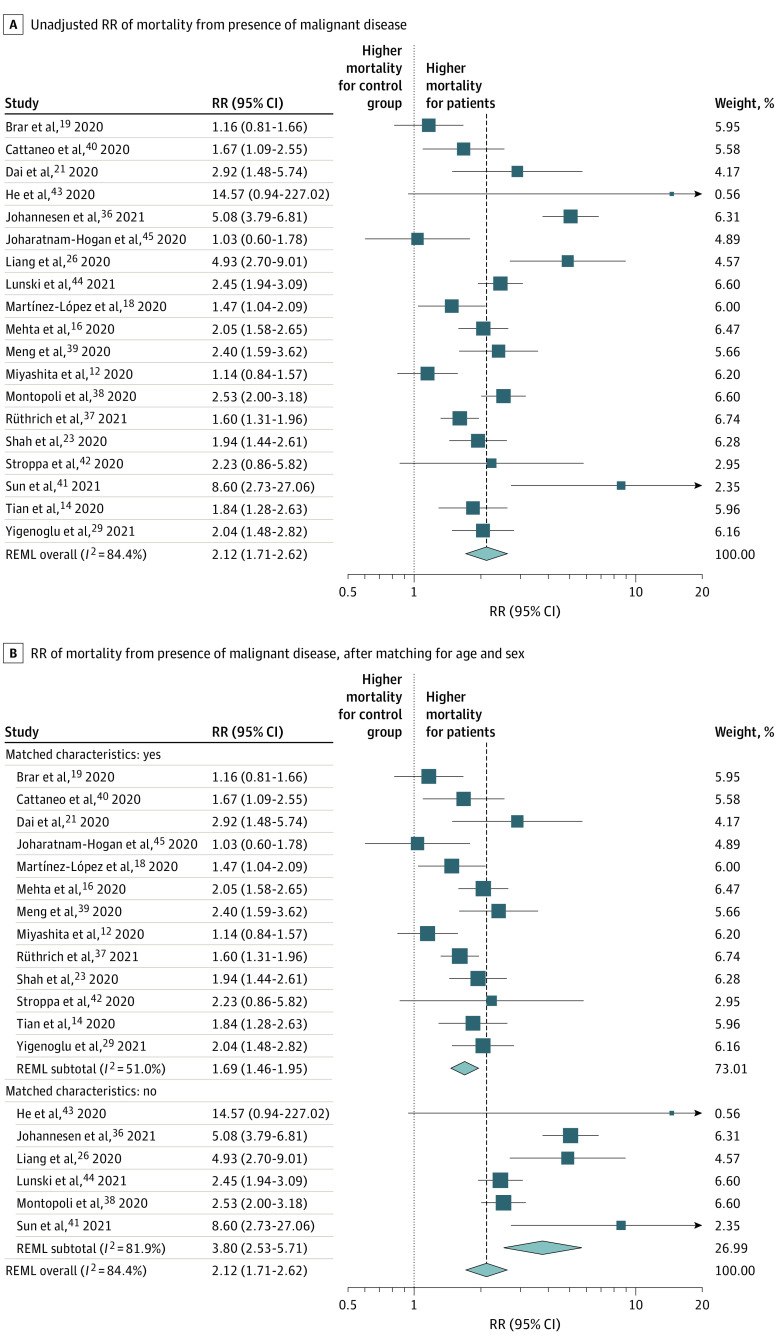
Forest Plot of Relative Risk (RR) of Mortality Weights were calculated using random-effects analysis. REML indicates restricted maximum likelihood.

We conducted a meta-analysis of these 19 studies. The pooled relative risk (RR) of mortality in patients with cancer and SARS-CoV-2 infection compared with control patients was 2.12 (95% CI, 1.71-2.62; *P* < .001; *I*^2^ = 84.4%) (Figure 1A). When pooling the results, the RR for mortality in 13 studies that matched for age decreased to 1.69 (95% CI, 1.46-1.95; *P* < .001; *I*^2^ = 51.0%) compared with 3.80 (95% CI, 2.53-5.71; *P* < .001; *I*^2^ = 81.9%) from 6 studies without matching (Figure 1B). There was little difference in the pooled RR of mortality between patients with any type of malignant neoplasm, solid or hematologic (RR, 2.23; 95% CI, 1.68-2.95; *I*^2^ = 88.7%), and hematologic cancer (RR, 1.81; 95% CI, 1.53-2.15; *I*^2^ = 0.0%) alone vs control patients (eFigure 14 in the Supplement).

**Figure 2.**
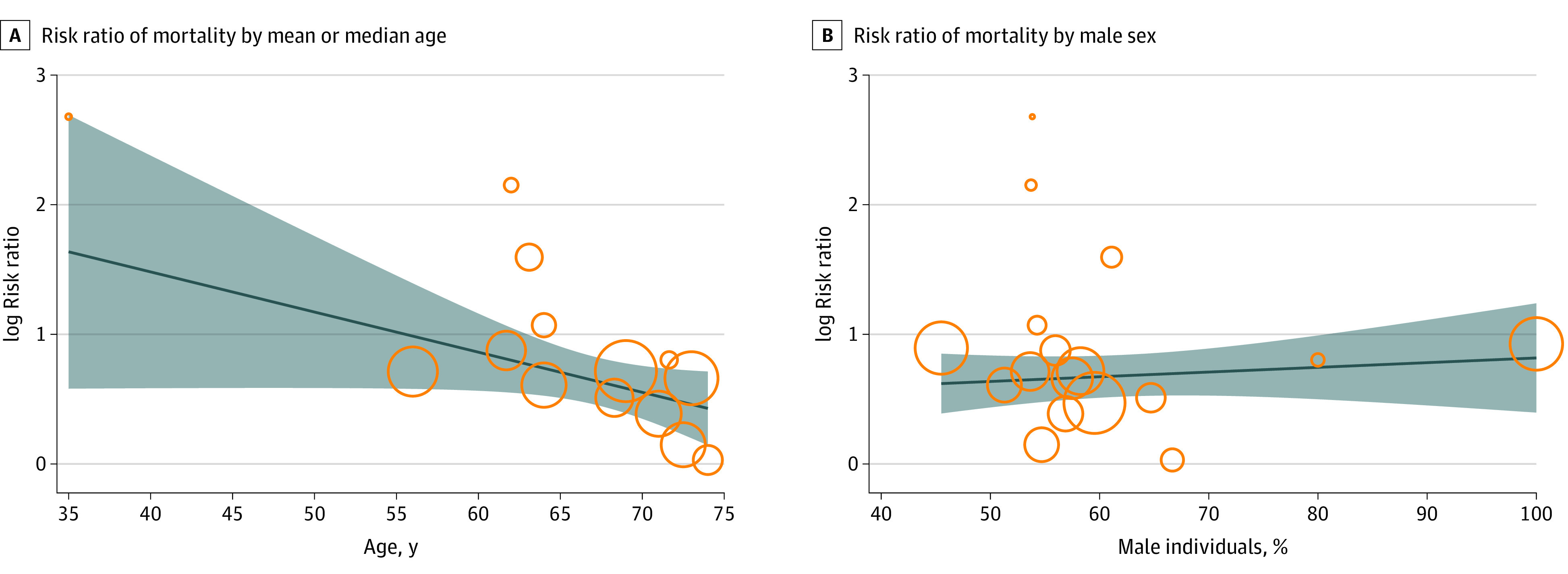
Meta-regression Bubble Plot of Association of Mortality With Age and Sex of Patients vs Control Group

Fourteen studies provided data on the median or mean age of patients with cancer and SARS-CoV-2 infection and control patients.^14,16,18,19,21,23,26,29,39,40,41,42,43,45^ On univariate regression, when assessing the association of age with mortality in patients with cancer vs control patients, the RR of mortality statistically significantly decreased as age increased (exp [b], 0.96; 95% CI, 0.92-0.99; *P* = .03), showing a greater difference in the RR of mortality between patients with cancer and control patients of younger age (Figure 2A). Seventeen studies reported the sex (as proportion of male patients) of both patients with cancer and control patients.^14,16,18,19,21,23,26,29,37,38,39,40,41,42,43,44,45^ A small increase in RR of mortality between patients with cancer and control patients was shown as the proportion of male patients in the study increased, but this finding was not statistically significant (exp [b], 1.19; 95% CI, 0.22-6.37; *P* = .83) (Figure 2B). When the combined impact of age and proportion of male patients was explored by multivariable regression, age remained significant (exp [b], 0.95; 95% CI, 0.91-0.99; *P* = .03), but male sex was not significant (exp [b], 3.5; 95% CI, 0.04-300.71; *P* = .55) (eTable 9 in the Supplement).

### Clinical Outcomes

Outcome data were available for 56 932 patients with cancer and SARS-CoV-2 infection from 68 of the 81 studies (84%).^7,8,9,10,11,12,13,14,15,16,17,18,19,20,21,22,23,24,25,26,27,28,29,31,32,33,34,35,36,37,38,39,40,41,42,43,44,45,46,47,48,49,50,51,52,53,54,55,56,57,58,59,66,67,68,69,70,74,75,77,78,81,82,83,84,87,89,90^ Where reported, 7% of patients (1170 of 16 409) were admitted to the intensive care unit, and 5% of patients (2817 of 54 298) required invasive mechanical ventilation (eTable 10 in the Supplement). At the time of reporting, 11% of patients (473 of 4403) remained hospitalized, and 65% of patients (2841 of 4403) were discharged. Median duration of hospital stay is shown in eTable 11 in the Supplement. Of the 56 932 patients with cancer and SARS-CoV-2 infection, 12% died (6813). Median follow-up times varied, ranging from 5 to 69 days. Mortality was reported in all studies and ranged from 4% to 61% (eFigure 15 and eTable 12 in the Supplement). One study defined mortality as either transfer to hospice or death.^22^

Fourteen different definitions of severe events were used across the studies (eFigure 16 in the Supplement). In 12 studies, information on ethnicity and outcomes was reported^7,8,13,23,48,53,54,56,59,70,84,89^ (eResults in the Supplement). In unadjusted analyses, several factors were found to be associated with severe events or death. These factors included increasing age (reported in 36 studies^9,10,11,13,14,15,16,17,18,19,20,21,24,25,27,30,31,32,33,37,40,42,43,44,46,47,48,49,52,54,58,68,69,77,87,88^) as well as increased levels of proinflammatory markers (reported in 14 studies^14,16,23,35,43,53,58,66,69,74,76,77,85,87^) and infection-related markers (reported in 14 studies^14,23,25,31,42,47,58,68,69,74,77,85,87,88^) (eFigure 17 in the Supplement). Factors that were found in adjusted analysis to be associated with worsening severity or mortality are shown in eFigure 17 in the Supplement; 22 studies found increasing age to be associated with worsening severity or death.^7,8,9,10,11,12,13,14,15,16,17,18,19,20,21,22,23,24,25,26,27,47^ Factors included in adjusted analysis are listed in eFigure 18 in the Supplement.

### Mortality and Case Fatality Rate by Cancer Type

Forest plots for pooled case fatality rate for each cancer type are shown in eFigure 19 in the Supplement. The association of the stage of malignant neoplasm with clinical outcomes is summarized in the eResults in the Supplement. There was a pooled case fatality rate for patients with breast cancer and SARS-CoV-2 infection of 9% (95% CI, 7%-12%; *I*^2^ = 89.8%; range, 0%-100%), with an RR of mortality of 0.51 (95% CI, 0.36-0.71; *P* < .001; *I*^2^ = 86.2%) compared with control patients (eFigure 20 in the Supplement). The pooled case fatality rate for patients with gynecological cancers and SARS-CoV-2 infection was 12% (95% CI, 8%-16%; *I*^2^ = 38.47%; range, 0%-38%) and an associated RR of 0.76 (95% CI, 0.62-0.93; *P* = .009; *I*^2^ = 0%) compared with control patients (eFigure 20 in the Supplement).

Gastrointestinal cancers in patients with SARS-CoV-2 infection were associated with a pooled case fatality rate of 16% (95% CI, 12%-20%; *I*^2^ = 78.66%; range, 0%-38%) and an RR of 1.13 (95% CI, 0.93-1.37; *P* = .21; *I*^2^ = 54.8%) compared with control patients (eFigure 20 in the Supplement). Skin cancer in patients with SARS-CoV-2 infection was associated with a pooled case fatality rate of 10% (95% CI, 5%-15%; *I*^2^ = 62.57%; range, 5%-50%) and an RR of mortality of 0.85 (95% CI, 0.60-1.20; *P* = .35; *I*^2^ = 51.4%) (eFigure 20 in the Supplement).

The pooled case fatality rate for patients with lung cancer and SARS-CoV-2 infection was 30% (95% CI, 24%-37%; *I*^2^ = 83.71%; range, 0%-60%). The RR of mortality in those with lung cancer compared with other cancer types was significantly higher at 1.68 (95% CI, 1.45-1.94; *P* < .001; *I*^2^ = 32.9%) (eFigure 20 in the Supplement). The pooled case fatality rate for patients with genitourinary cancers and SARS-CoV-2 infection was 22% (95% CI, 16%-27%; *I*^2^ = 92.61%; range, 8%-50%), with an RR of mortality of 1.11 (95% CI, 1.00-1.24; *P* = .06; *I*^2^ = 21.5%) compared with control patients (eFigure 20 in the Supplement). The pooled case fatality rate for patients with hematologic cancer and SARS-CoV-2 infection was 32% (95% CI, 28%-37%; *I*^2^ = 93.10%; range, 11%-100%). The overall RR of mortality in patients with hematologic cancer and SARS-CoV-2 infection compared with those with solid malignant neoplasms was 1.42 (95% CI, 1.31-1.54; *P* < .001; *I*^2^ = 6.8%) (eFigure 20 in the Supplement).

### Cancer Treatment and Course of COVID-19 

Data on timing of cancer treatment and outcome were available in 64 of 81 studies (79%).^7,8,9,10,11,13,14,15,16,17,18,19,20,21,22,24,25,26,27,28,30,31,32,33,34,35,36,37,38,39,40,42,43,44,45,46,47,48,49,50,51,52,53,54,55,56,58,59,66,67,68,69,70,73,74,76,77,78,81,82,83,85,87,88^ These studies comprised 54 335 patients, of whom 7567 (14%) had undergone or received some form of treatment for their malignant disease. Where data were available, the most common treatment modality was chemotherapy (3792 patients), followed by targeted therapy (1700 patients) and immunotherapy (817 patients). A total of 3896 patients had not received any form of treatment. The pooled case fatality rates for various cancer treatments is shown in eTable 13 in the Supplement.

**Figure 3.**
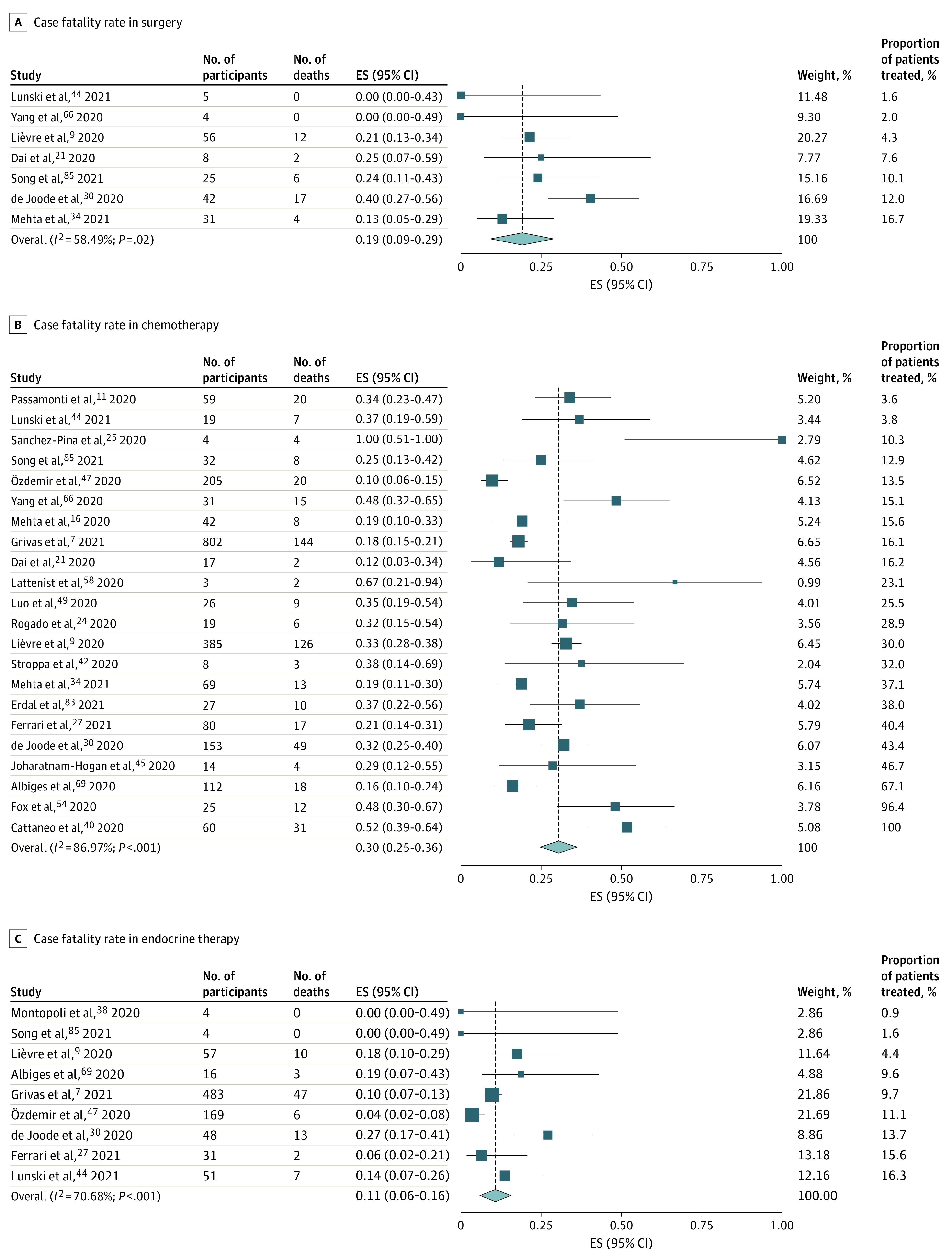
Forest Plot of Overall Case Fatality Rate for Surgery, Chemotherapy, and Endocrine Therapy ES indicates effect size.

Surgery within 3 months of a COVID-19 diagnosis in patients with cancer was associated with a pooled case fatality rate of 19% (95% CI, 9%-29%; *I*^2^ = 58.49%; range, 0%-40%) (Figure 3A). In general, these studies examined all patients, aside from 1 study that was focused on patients who underwent surgery for gynecological malignant neoplasms.^48^ Patients with cancer who had received chemotherapy and had a COVID-19 diagnosis had an overall pooled case fatality rate of 30% (95% CI, 25%-36%; *I*^2^ = 86.97%; range, 10%-100%) (Figure 3B), whereas patients who had endocrine therapy had a pooled case fatality rate of 11% (95% CI, 6%-16%; *I*^2^ = 70.68%; range, 0%-27%) (Figure 3C). None of the 9 studies specified which cancer type was treated with endocrine therapy.^7,9,27,30,38,44,47,69,85^

**Figure 4.**
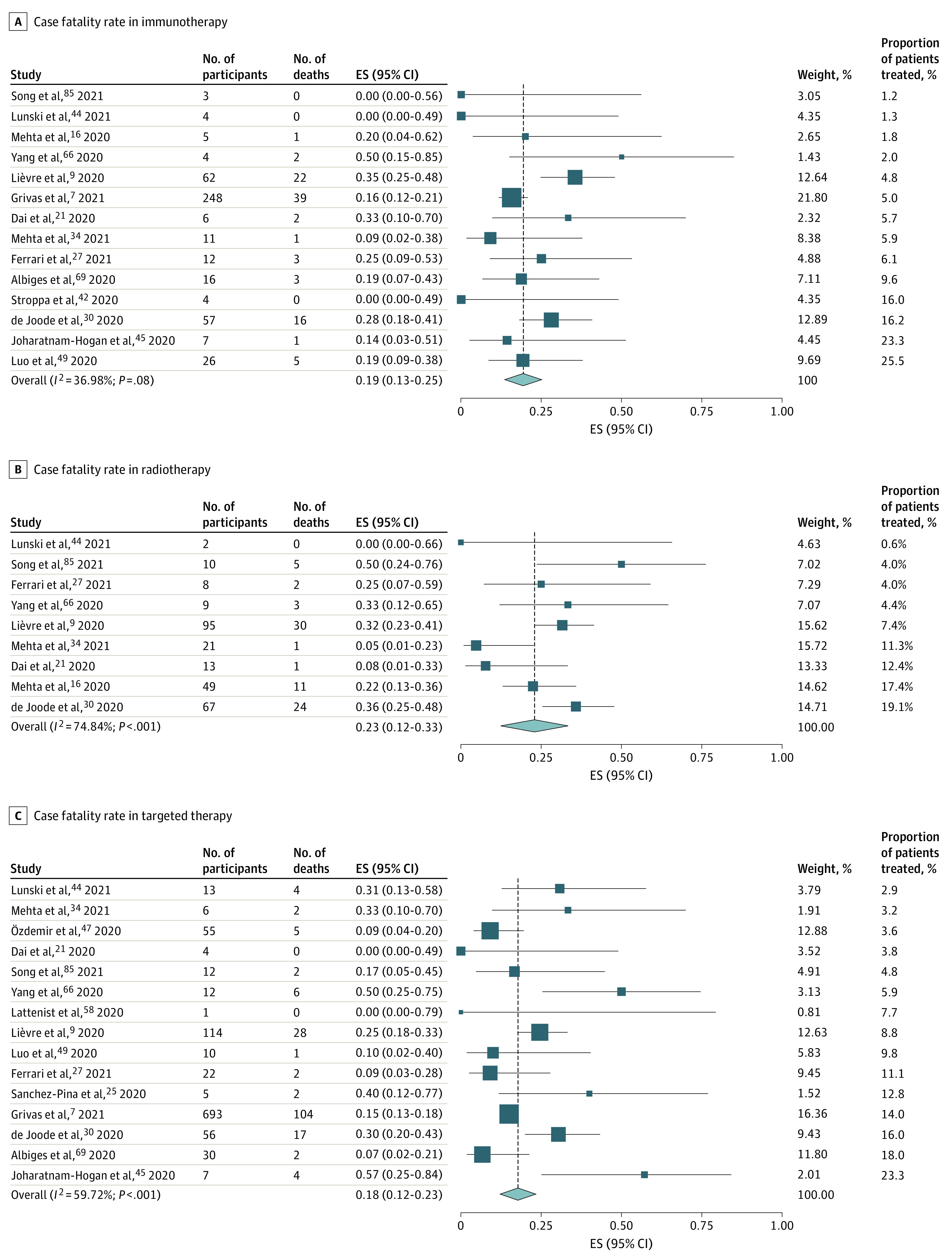
Forest Plot of Overall Case Fatality Rate for Immunonotherapy, Radiotherapy, and Targeted Therapy ES indicates effect size.

Immunotherapy was associated with a pooled case fatality rate of 19% (95% CI, 13%-25%; *I*^2^ = 36.98%; range, 0%-50%) (Figure 4A). One of the immunotherapy studies specified the tumor type involved (lung cancer^49^), but the remaining immunotherapy studies did not specify the tumor type.^7,9,16,21,27,30,34,42,44,45,66,69,85^ Data on mortality after radiotherapy were provided in 9 studies,^9,16,21,27,30,34,44,66,85^ and radiotherapy was associated with a pooled case fatality rate of 23% (95% CI, 12%-33%; *I*^2^ = 74.84%; range, 0%-50%) (Figure 4B). The tumor type treated (gynecological cancer) was specified in only 1 study.^48^ Targeted therapy was associated with an overall risk of mortality of 18% (95% CI, 12%-23%; *I*^2^ = 59.72%; range, 0%-57%) (Figure 4C). The nature of the targeted therapy was not specified in most cases, and the specific cancer type was stated in only 4 of 19 studies (21%), of which 2 involved patients with hematologic cancer,^25,58^ 1 involved patients with lung cancer,^49^ and 1 involved patients with gynecological cancer and SARS-CoV-2 infection.^48^

## Discussion

Population data at the start of the SARS-CoV-2 pandemic rapidly identified patients with cancer as a group with poor outcomes.^4^ Subsequent reports on SARS-CoV-2 infection in patients with cancer have ranged from small series^26,43,57,58,81,82,90^ to larger registry-based collaborative studies^4,7,37,46,47,92,93^ and have varied in geographical location and tumor type focus (eTable 4 in the Supplement). In this systematic review and meta-analysis, we analyzed the important global effort by the cancer research community by reviewing all of the available and published data as of June 14, 2021. Our objective was to assess the clinical outcomes among patients with cancer and SARS-CoV-2 infection vs the outcomes among control patients; we also aimed to identify patients with cancer who are at high risk for a poor outcome.

This systematic review found a number of potential limitations with the available data and literature, including (1) lack of contemporaneous populations without cancer for comparative analysis^12,19,23,36,38,40^; (2) heterogeneity of definitions between studies, such as severity of COVID-19 as demonstrated by the 14 definitions of severity used across studies (eFigure 16 in the Supplement); (3) predominantly retrospective nature of the studies (61 of 81) (eTable 4 in the Supplement); (4) variable follow-up times; (5) heterogeneity or poor description of the control cohorts, such as inclusion of patients who were not hospitalized^12,21,29,36,37,38,44^ or health care workers with COVID-19^43^; and (6) lack of detail on the systemic cancer treatment used.^4,11,12,22,29,33,37,39,41,42,50,52,55,56,57,65,70,71,72,75,76,79,80,81,84,86,87,88,89,90,91^ In studies with a control cohort, the data were generally historical or based on registry data^36,37^ or were not contemporaneous with the cancer cohort.^43^ Only 3 of the 19 studies that compared patients with cancer and SARS-CoV-2 infection with control patients used propensity score matching.^14,16,39^ Therefore, within the current literature, biases from unmeasured confounders may be present.

Analyzing 19 studies that involved 3926 patients with cancer and SARS-CoV-2 infection and 38 847 control patients, we found that malignant disease was associated with an increased risk of severe COVID-19 or death compared with the risk in control patients (RR, 2.12; 95% CI, 1.76-2.62; *P* < .001; *I*^2^ = 84.4%). However, when patients were matched for age and sex, the risk decreased to 1.69 (95% CI, 1.46-1.95; *P* < .001; *I*^2^ = 51.0%). This finding demonstrates a potential overestimation of the true risk to patients with cancer in studies that did not adjust for age and sex. Furthermore, it highlights the importance of a comparator control cohort in understanding the true implications of SARS-CoV-2 infection within a population with cancer. No significant sex-based differences in outcome were seen when patients with cancer and control patients were compared, in contrast to a number of studies that have reported male sex as a risk factor.^7,10,18,22,23,28,31,66^ However, the proportion of male patients included in each study varied, which may introduce uncertainty to the interpretation of the results of the meta-regression.

In the regression analysis, we found that younger age in patients with cancer and SARS-CoV-2 infection was associated with a worse clinical outcome than in age-matched control cohorts. To date, all of the cohort studies, which by their nature lacked an age-matched control group, have consistently reported increasing age as a risk factor for poor clinical outcome.^7,8,9,10,11,12,13,14,15,16,17,18,19,20,21,22,23,24,25,26,27^ Although it is true that older patients have worse absolute outcomes than younger patients, the RR data we found were highest for younger patients. This observation has been reported within the International Severe Acute Respiratory and Emerging Infections Consortium World Health Organization Clinical Characterization Protocol UK.^92,93^ A recent analysis of more than 20 000 patients with cancer vs 155 000 patients without cancer found that patients younger than 50 years, particularly those receiving active cancer treatment, were 5 times more likely to die than patients without cancer of a similar age (HR, 5.22; 95% CI, 4.19-6.52; *P* < .001).^93^ Compared with patients without cancer, the RR of death (the cancer attributable risk) decreased with age.^93^ The reasons for this finding were likely associated with the type of cancer, the intensity of treatments, or behavioral factors such as increased social mixing vs that of an older population.

We found that patients with lung cancer, followed by those with hematologic cancer, were at greatest risk of mortality from COVID-19, compared with patients with other cancers. Hematologic cancers have been consistently reported as a risk factor for poor clinical outcomes.^7,8,11,23,28,31,32^ The increased susceptibility to poor outcomes among patients with hematologic cancer is consistent with the more profound immune suppression that affects this patient group, whereas the increase in mortality among patients with lung cancer is likely associated with age, reduced lung reserve, comorbidities, and cancer treatment. The reason for the lower mortality from COVID-19 that we found in patients with breast and gynecological cancers is not clear. It could be associated with the protective feature of the female sex, although we found no sex-based difference in outcomes within the meta-analysis we conducted. An alternative explanation could be low circulating estradiol levels often seen in patients with breast and gynecological cancers. Use of androgen deprivation therapy in prostate cancer has been associated with protection from SARS-CoV-2 infection.^38^ There is a need to understand if a similar outcome is seen in female patients with lower estradiol levels.

In the pooled case fatality analysis, we found that endocrine therapy had the lowest pooled case fatality rate at 11% and chemotherapy had the highest at 30%. The higher mortality seen with chemotherapy compared with other treatments was likely associated with the immunosuppression after chemotherapy. Cohort studies have reported disparate results regarding the risk of chemotherapy.^8,9,13,14,15,16,20,24,25,26,27,28,30,31,34,35,36,40,46,48,58,66,68,74,85^ Given the lack of patient-level data, we were unable to define the risk of mortality for patients who were receiving anticancer therapy and contracted COVID-19. Similarly, we could not compare the implications of cancer treatment for the risk of mortality between these patients and control patients. A comparison of risk by different treatment modalities and by individual drugs also was not possible given that it was not clear if patients had received more than 1 treatment modality and the individual drugs were not named. More granular data, as well as use of a population without cancer controlled by age and sex, are needed to ascertain the risk of different anticancer therapies in the context of COVID-19.

Ongoing studies such as the International Severe Acute Respiratory and Emerging Infections Consortium World Health Organization Clinical Characterization Protocol UK^93^ will enable a more comprehensive comparison of patients with cancer vs control patients, with adjustments for age, sex, and other comorbidities, and the identification of the true risk of different tumor types and treatments while controlling for patient-level factors. In addition, studies are needed that assess the outcome of mitigation and treatment measures over the course of the SARS-CoV-2 pandemic between patients with cancer and control patients given that many of the treatment studies did not recruit patients with cancer. eTable 14 in the Supplement lists completed and current studies relevant to this topic.

The global effort to understand the implications of SARS-CoV-2 infection for patients with cancer has resulted in a rich data resource that should be used for an individual patient-level meta-analysis. Such data will maximize learning and knowledge and may be used to prepare the cancer research community for subsequent pandemics, which will inevitably occur.

### Limitations

This study has several limitations. First, we assessed outcomes in 7 tumor types as outcome data because these were most frequently reported in the studies we analyzed. Second, it was not possible to compare patients with solid cancers with a control cohort because we found no suitable studies that identified these cancer types. Third, we were unable to explore the potential outcomes of different SARS-CoV-2 variants because this information was not available. Furthermore, interpretation of the funnel plots may be difficult because of the heterogeneous evidence base and the presence of observational studies. Fourth, the data included in this meta-analysis were from the prevaccination and antiviral medication phases of the SARS-CoV-2 pandemic; therefore, vaccinations and active treatments may have affected our observations.

## Conclusions

This large, comprehensive systematic review and meta-analysis found a higher risk of death from COVID-19 in patients with cancer than in patients without cancer, although the risk was lower than that reported in individual cohort studies. Younger patients were at a particularly increased risk for poor clinical outcomes compared with age-matched control patients. Patients with lung cancer had the highest risk of mortality, followed by those with hematologic cancer. Given these data, younger patients may be considered in certain settings to be a high-risk population for poor outcomes from COVID-19.

## Notes

Supplement. **eTable 1.** Search Strategy Used for the Systematic Review in PubMed **eTable 2.** Search Strategy Used for the Systematic Review in Web of Science **eTable 3.** Search Strategy Used for the Systematic Review in Scopus **eFigure 1.** Newcastle-Ottawa Scale for Quality Assessment of the Included Studies **eFigure 2.** Funnel Plot of the Main Analysis of Mortality in Cancer Patients With COVID-19 to Control Patients **eFigure 3.** PRISMA Chart for Study Selection **eFigure 4.** Number of Cancer Patients Across 81 Studies, a Total of 61,532 Patients **eTable 4.** Overview of Studies Included in Review **eFigure 5.** Distribution of Cancer Patients Across 80 Studies **eFigure 6.** Countries Included Across All Studies **eTable 5.** Distribution of Cancer Patients by County **eFigure 7.** Co-morbidities Experienced by Cancer Patients, Representing 21,697 Patients Across 56 Studies **eFigure 8.** Percentage of Cancer Patients Without Co-morbidities, or With One or More Co-morbidities, Where Reported **eFigure 9.** Summary of Reported Co-morbidities **eFigure 10.** Chest Radiograph Chest X Ray Imaging on Admission With Radiological Changes Documented, Where Reported **eFigure 11.** Setting of Care Across 80 Studies, Representing 22,918 Cancer Patients **eTable 6.** Inpatient vs Outpatient Care Across 73 Studies as Well as Possible or Probable Nosocomial Infection **eTable 7.** Tumour Prevalence Across Studies Included **eFigure 12.** Tumour Type Breakdown Across 68 Studies, Where Reported **eResults.** Presenting Symptoms of SARS-CoV-2 Infection, Radiological Findings, Ethnicity and Stage of Malignancy and Outcome
eReferences
**eFigure 13.** Presenting Symptoms, Includes 9,196 Patients **eTable 8.** Chest Radiograph ± Chest X Ray Imaging on Admission With Radiological Changes Documented, Where Reported *Nodules/Interstitial Thickening/Erratic Paving **eFigure 14.** Forest Plot of Relative Risk of Mortality in Subgroup Analysis **eTable 9.** Meta-Regression Results on the Impact of (A) Age, (B) Male Gender, and (C) Both Age and Male Gender **eTable 10.** Patient Outcomes in 68 Studies **eTable 11.** Median Duration of Hospital Stay (Days) **eFigure 15.** Mortality Reported Across 81 Studies **eTable 12.** Mortality of Cancer Patients Reported Across Studies **eFigure 16.** Definition of Severe Event **eFigure 17.** Significant Variables in Unadjusted (A) and Adjusted (B) Analyses Across Studies **eFigure 18.** Variables Included in Adjusted Analysis Across Studies **eFigure 19.** Forest Plot of Overall Pooled Case Fatality in Subgroup Analysis **eTable 13.** Pooled Case Fatality Rates for Various Cancer Treatments **eTable 14.** Table of Completed and Ongoing Cancer Observational Studies Related to the SARS-CoV-2/COVID-19 Pandemic in Patients With Malignant Disease **eFigure 20.** Forest Plot of Relative Risk of Mortality in Different Cancer Types in the Subgroup Analysis
