# Supplementary material for: Differences in Outcomes and Factors Associated With Mortality Among Patients With SARS-CoV-2 Infection and Cancer Compared With Those Without Cancer: A Systematic Review and Meta-analysis
Source: JAMA Netw Open. 2022 May 9;5(5):e2210880. doi: 10.1001/jamanetworkopen.2022.10880 (PMC9086843; doi:10.1001/jamanetworkopen.2022.10880)
Supplement: Supplement. — eTable 1. Search Strategy Used for the Systematic Review in PubMed eTable 2. Search Strategy Used for the Systematic Review in Web of Science eTable 3. Search Strategy Used for the Systematic Review in Scopus eFigure 1. Newcastle-Ottawa Scale for Quality Assessment of the Included Studies eFigure 2. Funnel Plot of the Main Analysis of Mortality in Cancer Patients With COVID-19 to Control Patients eFigure 3. PRISMA Chart for Study Selection eFigure 4. Number of Cancer Patients Across 81 Studies, a Total of 61,532 Patients eTable 4. Overview of Studies Included in Review eFigure 5. Distribution of Cancer Patients Across 80 Studies eFigure 6. Countries Included Across All Studies eTable 5. Distribution of Cancer Patients by County eFigure 7. Co-morbidities Experienced by Cancer Patients, Representing 21,697 Patients Across 56 Studies eFigure 8. Percentage of Cancer Patients Without Co-morbidities, or With One or More Co-morbidities, Where Reported eFigure 9. Summary of Reported Co-morbidities eFigure 10. Chest Radiograph Chest X Ray Imaging on Admission With Radiological Changes Documented, Where Reported eFigure 11. Setting of Care Across 80 Studies, Representing 22,918 Cancer Patients eTable 6. Inpatient vs Outpatient Care Across 73 Studies as Well as Possible or Probable Nosocomial Infection eTable 7. Tumour Prevalence Across Studies Included eFigure 12. Tumour Type Breakdown Across 68 Studies, Where Reported eResults. Presenting Symptoms of SARS-CoV-2 Infection, Radiological Findings, Ethnicity and Stage of Malignancy and Outcome eReferences eFigure 13. Presenting Symptoms, Includes 9,196 Patients eTable 8. Chest Radiograph ± Chest X Ray Imaging on Admission With Radiological Changes Documented, Where Reported *Nodules/Interstitial Thickening/Erratic Paving eFigure 14. Forest Plot of Relative Risk of Mortality in Subgroup Analysis eTable 9. Meta-Regression Results on the Impact of (A) Age, (B) Male Gender, and (C) Both Age and Male Gender eTable 10. Patient Outcome [file jamanetwopen-e2210880-s001.pdf]

Supplementary Online Content

Khoury E, Nevitt S, Madsen WR, Turtle L, Davies G, Palmieri C. Differences in outcomes and factors associated with mortality among patients with SARS-CoV-2 infection and cancer compared with those without cancer: a systematic review and meta-analysis. *JAMA Netw Open*. 2022;5(5):e2210880. doi:10.1001/jamanetworkopen.2022.10880

eTable 1. Search Strategy Used for the Systematic Review in PubMed

eTable 2. Search Strategy Used for the Systematic Review in Web of Science

eTable 3. Search Strategy Used for the Systematic Review in Scopus

eFigure 1. Newcastle-Ottawa Scale for Quality Assessment of the Included Studies

eFigure 2. Funnel Plot of the Main Analysis of Mortality in Cancer Patients With COVID-19 to Control Patients

eFigure 3. PRISMA Chart for Study Selection

eFigure 4. Number of Cancer Patients Across 81 Studies, a Total of 61,532 Patients

eTable 4. Overview of Studies Included in Review

eFigure 5. Distribution of Cancer Patients Across 80 Studies

eFigure 6. Countries Included Across All Studies

eTable 5. Distribution of Cancer Patients by County

eFigure 7. Co-morbidities Experienced by Cancer Patients, Representing 21,697 Patients Across 56 Studies

eFigure 8. Percentage of Cancer Patients Without Co-morbidities, or With One or More Co-morbidities, Where Reported

eFigure 9. Summary of Reported Co-morbidities

eFigure 10. Chest Radiograph Chest X Ray Imaging on Admission With Radiological Changes Documented, Where Reported

eFigure 11. Setting of Care Across 80 Studies, Representing 22,918 Cancer Patients

eTable 6. Inpatient vs. Outpatient Care Across 73 Studies as Well as Possible or Probable Nosocomial Infection

eTable 7. Tumour Prevalence Across Studies Included

eFigure 12. Tumour Type Breakdown Across 68 Studies, Where Reported

eResults. Presenting Symptoms of SARS-CoV-2 Infection, Radiological Findings, Ethnicity and Stage of Malignancy and Outcome

eReferences

eFigure 13. Presenting Symptoms, Includes 9,196 Patients

eTable 8. Chest Radiograph ± Chest X Ray Imaging on Admission With Radiological Changes Documented, Where Reported *Nodules/Interstitial Thickening/Erratic Paving

eFigure 14. Forest Plot of Relative Risk of Mortality in Subgroup Analysis

eTable 9. Meta-Regression Results on the Impact of (A) Age, (B) Male Gender, and (C) Both Age and Male Gender

eTable 10. Patient Outcomes in 68 Studies

eTable 11. Median Duration of Hospital Stay (Days)

eFigure 15. Mortality Reported Across 81 Studies

eTable 12. Mortality of Cancer Patients Reported Across Studies

eFigure 16. Definition of Severe Event

eFigure 17. Significant Variables in Unadjusted (A) and Adjusted (B) Analyses Across Studies

eFigure 18. Variables Included in Adjusted Analysis Across Studies

eFigure 19. Forest Plot of Overall Pooled Case Fatality in Subgroup Analysis

eTable 13. Pooled Case Fatality Rates for Various Cancer Treatments

eTable 14. Table of Completed and Ongoing Cancer Observational Studies Related to the SARS-CoV-2/COVID-19 Pandemic in Patients With Malignant Disease

eFigure 20. Forest Plot of Relative Risk of Mortality in Different Cancer Types in the Subgroup Analysis

© 2022 Khoury E et al. *JAMA Network Open.*

This supplementary material has been provided by the authors to give readers additional information about their work.

© 2022 Khoury E et al. *JAMA Network Open.*

Search number	Query	Search Details	Results	Time	Date
**14**	(((((cancer) OR (malignan*)) OR (tumour)) OR (neoplasm)) AND ((((2019-nCoV pneumonia) OR (SARS-CoV-2)) OR (Coronavirus)) OR (COVID-19))) AND ((solid) OR (haematolog*))	("cancer s"[All Fields] OR "cancerated"[All Fields] OR "canceration"[All Fields] OR "cancerization"[All Fields] OR "cancerized"[All Fields] OR "cancerous"[All Fields] OR "neoplasms"[MeSH Terms] OR "neoplasms"[All Fields] OR "cancer"[All Fields] OR "cancers"[All Fields] OR "malignan*"[All Fields] OR ("cysts"[MeSH Terms] OR "cysts"[All Fields] OR "cyst"[All Fields] OR "neurofibroma"[MeSH Terms] OR "neurofibroma"[All Fields] OR "neurofibromas"[All Fields] OR "tumor s"[All Fields] OR "tumoral"[All Fields] OR "tumorous"[All Fields] OR "tumour"[All Fields] OR "neoplasms"[MeSH Terms] OR "neoplasms"[All Fields] OR "tumor"[All Fields] OR "tumour s"[All Fields] OR "tumoural"[All Fields] OR "tumourous"[All Fields] OR "tumours"[All Fields] OR "tumors"[All Fields]) OR ("neoplasm s"[All Fields] OR "neoplasms"[MeSH Terms] OR "neoplasms"[All Fields] OR "neoplasm"[All Fields])) AND ((("severe acute respiratory syndrome coronavirus 2"[Supplementary Concept] OR "severe acute respiratory syndrome coronavirus 2"[All Fields] OR "2019 ncov"[All Fields]) AND ("pneumonia"[MeSH Terms] OR "pneumonia"[All Fields] OR "pneumoniae"[All Fields] OR "pneumonias"[All Fields] OR "pneumoniae s"[All Fields])) OR ("severe acute respiratory syndrome coronavirus 2"[Supplementary Concept] OR "severe acute respiratory syndrome coronavirus 2"[All Fields] OR "sars cov 2"[All Fields]) OR ("coronavirus"[MeSH Terms] OR "coronavirus"[All Fields] OR "coronaviruses"[All Fields]) OR ("severe acute respiratory syndrome coronavirus 2"[Supplementary Concept] OR "severe acute respiratory syndrome coronavirus 2"[All Fields] OR "ncov"[All Fields] OR "2019 ncov"[All Fields] OR "covid 19"[All Fields] OR "sars cov 2"[All Fields] OR (("coronavirus"[All Fields] OR "cov"[All Fields]) AND 2019/11/01:3000/12/31[Date - Publication]))) AND ("solid"[All Fields] OR "solid s"[All Fields] OR "solids"[All Fields] OR "haematolog*"[All Fields])	512	09:21:54	14/06/2021
**13**	(solid) OR (haematolog*)	"solid"[All Fields] OR "solid s"[All Fields] OR "solids"[All Fields] OR "haematolog*"[All Fields]	490,032	09:21:21	14/06/2021
**12**	haematolog*	"haematolog*"[All Fields]	90,344	09:20:30	14/06/2021
**11**	solid	"solid"[All Fields] OR "solid s"[All Fields] OR "solids"[All Fields]	402,191	09:20:00	14/06/2021

© 2022 Khoury E et al. *JAMA Network Open.*

Search number	Query	Search Details	Results	Time	Date
**10**	(((2019-nCoV pneumonia) OR (SARS-CoV-2)) OR (Coronavirus)) OR (COVID-19)	(("severe acute respiratory syndrome coronavirus 2"[Supplementary Concept] OR "severe acute respiratory syndrome coronavirus 2"[All Fields] OR "2019 ncov"[All Fields]) AND ("pneumonia"[MeSH Terms] OR "pneumonia"[All Fields] OR "pneumoniae"[All Fields] OR "pneumonias"[All Fields] OR "pneumoniae s"[All Fields])) OR ("severe acute respiratory syndrome coronavirus 2"[Supplementary Concept] OR "severe acute respiratory syndrome coronavirus 2"[All Fields] OR "sars cov 2"[All Fields]) OR ("coronavirus"[MeSH Terms] OR "coronavirus"[All Fields] OR "coronaviruses"[All Fields]) OR ("severe acute respiratory syndrome coronavirus 2"[Supplementary Concept] OR "severe acute respiratory syndrome coronavirus 2"[All Fields] OR "ncov"[All Fields] OR "2019 ncov"[All Fields] OR "covid 19"[All Fields] OR "sars cov 2"[All Fields] OR (("coronavirus"[All Fields] OR "cov"[All Fields]) AND 2019/11/01:3000/12/31[Date - Publication]))	161,008	09:19:24	14/06/2021
**9**	COVID-19	"severe acute respiratory syndrome coronavirus 2"[Supplementary Concept] OR "severe acute respiratory syndrome coronavirus 2"[All Fields] OR "ncov"[All Fields] OR "2019 ncov"[All Fields] OR "covid 19"[All Fields] OR "sars cov 2"[All Fields] OR (("coronavirus"[All Fields] OR "cov"[All Fields]) AND 2019/11/01:3000/12/31[Date - Publication])	145,752	09:19:08	14/06/2021
**8**	coronavirus	"coronavirus"[MeSH Terms] OR "coronavirus"[All Fields] OR "coronaviruses"[All Fields]	116,456	09:19:00	14/06/2021
**7**	SARS-CoV-2	"severe acute respiratory syndrome coronavirus 2"[Supplementary Concept] OR "severe acute respiratory syndrome coronavirus 2"[All Fields] OR "sars cov 2"[All Fields]	89,189	09:18:53	14/06/2021
**6**	2019-nCoV pneumonia	("severe acute respiratory syndrome coronavirus 2"[Supplementary Concept] OR "severe acute respiratory syndrome coronavirus 2"[All Fields] OR "2019 ncov"[All Fields]) AND ("pneumonia"[MeSH Terms] OR "pneumonia"[All Fields] OR "pneumoniae"[All Fields] OR "pneumonias"[All Fields] OR "pneumoniae s"[All Fields])	71,085	09:18:43	14/06/2021
**5**	(((cancer) OR (malignan*)) OR (tumour)) OR (neoplasm)	"cancer s"[All Fields] OR "cancerated"[All Fields] OR "canceration"[All Fields] OR "cancerization"[All Fields] OR "cancerized"[All Fields] OR "cancerous"[All Fields] OR "neoplasms"[MeSH Terms] OR "neoplasms"[All Fields] OR "cancer"[All Fields] OR "cancers"[All Fields] OR "malignan*"[All Fields] OR "cysts"[MeSH Terms] OR "cysts"[All Fields] OR "cyst"[All Fields] OR "neurofibroma"[MeSH Terms] OR "neurofibroma"[All Fields] OR "neurofibromas"[All Fields] OR "tumor s"[All Fields] OR "tumoral"[All Fields] OR "tumorous"[All Fields] OR "tumour"[All Fields] OR "neoplasms"[MeSH Terms] OR "neoplasms"[All Fields] OR "tumor"[All Fields] OR "tumour s"[All Fields] OR "tumoural"[All Fields] OR "tumourous"[All Fields] OR "tumours"[All Fields] OR "tumors"[All Fields] OR "neoplasm s"[All Fields] OR "neoplasms"[MeSH Terms] OR "neoplasm"[All Fields] OR "neoplasms"[All Fields] OR "neoplasm"[All Fields]	4,997,027	09:18:33	14/06/2021

© 2022 Khoury E et al. *JAMA Network Open.*

Search number	Query	Search Details	Results	Time	Date
**4**	neoplasm	"neoplasm s"[All Fields] OR "neoplasms"[MeSH Terms] OR "neoplasms"[All Fields] OR "neoplasm"[All Fields]	3,558,546	09:18:19	14/06/2021
**3**	tumour	"cysts"[MeSH Terms] OR "cysts"[All Fields] OR "cyst"[All Fields] OR "neurofibroma"[MeSH Terms] OR "neurofibroma"[All Fields] OR "neurofibromas"[All Fields] OR "tumor s"[All Fields] OR "tumoral"[All Fields] OR "tumorous"[All Fields] OR "tumour"[All Fields] OR "neoplasms"[MeSH Terms] OR "neoplasms"[All Fields] OR "tumor"[All Fields] OR "tumour s"[All Fields] OR "tumoural"[All Fields] OR "tumourous"[All Fields] OR "tumours"[All Fields] OR "tumors"[All Fields]	4,274,667	09:18:14	14/06/2021
**2**	malignan*	"malignan*"[All Fields]	621,529	09:18:07	14/06/2021
**1**	cancer	"cancer s"[All Fields] OR "cancerated"[All Fields] OR "canceration"[All Fields] OR "cancerization"[All Fields] OR "cancerized"[All Fields] OR "cancerous"[All Fields] OR "neoplasms"[MeSH Terms] OR "neoplasms"[All Fields] OR "cancer"[All Fields] OR "cancers"[All Fields]	4,373,309	09:18:01	14/06/2021

eTable 1 Search strategy used for the systematic review in PubMed.

© 2022 Khoury E et al. *JAMA Network Open.*

Set	Results	Search History	Date
#14	232	#13 AND #10 AND #5<br>*Indexes=SCI-EXPANDED, SSCI, A&HCI, CPCI-S, CPCI-SSH, BKCI-S, BKCI-SSH, ESCI, CCR-EXPANDED, IC Timespan=All years*	14/06/2021
#13	1,402,806	#12 OR #11<br>*Indexes=SCI-EXPANDED, SSCI, A&HCI, CPCI-S, CPCI-SSH, BKCI-S, BKCI-SSH, ESCI, CCR-EXPANDED, IC Timespan=All years*	14/06/2021
#12	29,734	**TOPIC:** (haematolog*)<br>*Indexes=SCI-EXPANDED, SSCI, A&HCI, CPCI-S, CPCI-SSH, BKCI-S, BKCI-SSH, ESCI, CCR-EXPANDED, IC Timespan=All years*	14/06/2021
#11	1,374,645	**TOPIC:** (solid)<br>*Indexes=SCI-EXPANDED, SSCI, A&HCI, CPCI-S, CPCI-SSH, BKCI-S, BKCI-SSH, ESCI, CCR-EXPANDED, IC Timespan=All years*	14/06/2021
#10	156,260	#9 OR #8 OR #7 OR #6<br>*Indexes=SCI-EXPANDED, SSCI, A&HCI, CPCI-S, CPCI-SSH, BKCI-S, BKCI-SSH, ESCI, CCR-EXPANDED, IC Timespan=All years*	14/06/2021
#9	128,800	**TOPIC:** (COVID-19)<br>*Indexes=SCI-EXPANDED, SSCI, A&HCI, CPCI-S, CPCI-SSH, BKCI-S, BKCI-SSH, ESCI, CCR-EXPANDED, IC Timespan=All years*	14/06/2021
#8	69,653	**TOPIC:** (Coronavirus)<br>*Indexes=SCI-EXPANDED, SSCI, A&HCI, CPCI-S, CPCI-SSH, BKCI-S, BKCI-SSH, ESCI, CCR-EXPANDED, IC Timespan=All years*	14/06/2021
#7	39,067	**TOPIC:** (SARS-CoV-2)<br>*Indexes=SCI-EXPANDED, SSCI, A&HCI, CPCI-S, CPCI-SSH, BKCI-S, BKCI-SSH, ESCI, CCR-EXPANDED, IC Timespan=All years*	14/06/2021
#6	498	**TOPIC:** (2019-nCoV pneumonia)<br>*Indexes=SCI-EXPANDED, SSCI, A&HCI, CPCI-S, CPCI-SSH, BKCI-S, BKCI-SSH, ESCI, CCR-EXPANDED, IC Timespan=All years*	14/06/2021
#5	3,929,534	#4 OR #3 OR #2 OR #1<br>*Indexes=SCI-EXPANDED, SSCI, A&HCI, CPCI-S, CPCI-SSH, BKCI-S, BKCI-SSH, ESCI, CCR-EXPANDED, IC Timespan=All years*	14/06/2021
#4	201,475	**TOPIC:** (neoplasm)<br>*Indexes=SCI-EXPANDED, SSCI, A&HCI, CPCI-S, CPCI-SSH, BKCI-S, BKCI-SSH, ESCI, CCR-EXPANDED, IC Timespan=All years*	14/06/2021
#3	1,906,360	**TOPIC:** (tumour)<br>*Indexes=SCI-EXPANDED, SSCI, A&HCI, CPCI-S, CPCI-SSH, BKCI-S, BKCI-SSH, ESCI, CCR-EXPANDED, IC Timespan=All years*	14/06/2021
#2	604,111	**TOPIC:** (malignan*)<br>*Indexes=SCI-EXPANDED, SSCI, A&HCI, CPCI-S, CPCI-SSH, BKCI-S, BKCI-SSH, ESCI, CCR-EXPANDED, IC Timespan=All years*	14/06/2021
# 1	2,709,682	**TOPIC:** (cancer)<br>***Indexes=SCI-EXPANDED, SSCI, A&HCI, CPCI-S, CPCI-SSH, BKCI-S, BKCI-SSH, ESCI, CCR-EXPANDED, IC Timespan=All years***	14/06/2021

eTable 2. Search strategy used for the systematic review in Web of Science.

© 2022 Khoury E et al. *JAMA Network Open.*

Search Number	Search History	Document results	Date
**14**	((TITLE-ABS-KEY (cancer)) OR (TITLE-ABS-KEY (malignan*)) OR (TITLE-ABS-KEY (tumour)) OR (TITLE-ABS-KEY (neoplasm))) AND ((2019-ncov AND pneumonia) OR (sars-cov-2) OR (coronavirus) OR (covid-19)) AND ((solid) OR (haematolog*))	364	14/06/2021
**13**	(solid) OR (haematolog*)	1,797,587	14/06/2021
**12**	haematolog*	44,098	14/06/2021
**11**	solid	1,755,385	14/06/2021
**10**	(2019-ncov AND pneumonia) OR (sars-cov-2) OR (coronavirus) OR (covid-19)	192,773	14/06/2021
**9**	covid-19	155,108	14/06/2021
**8**	coronavirus	134,571	14/06/2021
**7**	sars-cov-2	63,082	14/06/2021
**6**	2019-ncov AND pneumonia	988	14/06/2021
**5**	(TITLE-ABS-KEY (cancer)) OR (TITLE-ABS-KEY (malignan*)) OR (TITLE-ABS-KEY (tumour)) OR (TITLE-ABS-KEY (neoplasm))	5,338,087	14/06/2021
**4**	neoplasm	2,712,836	14/06/2021
**3**	tumour	3,447,845	14/06/2021
**2**	malignan*	805,290	14/06/2021
**1**	Cancer	3,358,220	14/06/2021

eTable 3. Search strategy used for the systematic review in Scopus.

Study	Selection	Comparability	Outcome	Total
Sharafeldin et al	***	*	**	6
Grivas et al	***	*	***	7
Docherty et al	***	**	***	8
Özdemir et al	***	*	***	7
Lièvre et al	***	*	***	7
Lee et al	***	**	**	7
Fratino et al	**	-	*	3
Pinato et al	**	**	***	7
Yigenoglu et al	****	*	*	6
Johannesen et al	***	*	*	5
Passamonti et al	***	*	***	7
Rüthrich et al	****	*	*	6
Montopoli et al	***	-	*	4
Robilotti et al	***	-	***	6
de Joode et al	***	*	**	6
Miyashita et al	****	*	**	7
Graeselli et al	*	-	***	4
Lunski et al	****	**	***	9
Jee et al	***	-	***	6
Song et al	***	**	***	8
COVIDSurg Collaborative	***	**	***	8
Tian et al	***	**	*	6
Di Cosimo et al	***	*	***	7
Mehta V et al	***	**	**	7
Yang K et al	***	*	***	7
Ferrari et al	***	*	***	7
Garassino et al	**	**	**	6
Mato et al	***	*	***	7
Scarfo et al	***	-	***	6
Mehta A et al	***	**	***	8
de Melo et al	***	*	**	6
Albiges et al	***	**	***	8
Martinez-Lopez et al	****	*	*	6
Russell et al	***	-	***	6
Basse et al	***	*	**	6
Lara et al	***	*	*	5
Brar et al	****	**	*	7
Angelis et al	***	**	*	6
Gupta et al	**	*	***	6
Meng et al	****	*	**	7
Deng et al	**	-	*	3
Kabarriti et al	***	-	*	4
Zhang H et al	*	*	**	4
Dai et al	****	*	***	8
Luo et al	***	-	***	6
Cattaneo et al	***	-	***	6
Hultcrantz et al	***	*	***	7
Singh et al	***	**	***	8
Shah et al	****	*	*	6
Cook et al	***	-	*	4
Erdal et al	***	-	***	6
Sun et al	****	**	*	7
Booth et al	****	-	***	7
Yarza et al	****	-	***	7
Vuagnat et al	****	-	***	7
Wang B et al	***	-	**	5
Assaad et al	***	-	***	6
Fox et al	***	-	***	6
Yang F et al	***	-	*	4
Suleyman et al	***	**	*	6
Rogado et al	***	*	**	6
Sanchez-Pina et al	***	*	***	7
Ma et al	**	-	-	2
Aries et al	***	-	**	5
Martin-Moro et al	***	*	***	7
Joharatnam-Hogan et al	***	*	**	6
Zhang L et al	**	*	**	5
Kalinsky et al	***	-	***	6
Malard et al	***	-	*	4
Stroppa et al	*	*	**	4
Ciceri et al	**	*	***	6
Tomlins et al	**	-	***	5
Bogani et al	***	-	*	4
Liang et al	***	*	***	7
Guan et al	***	**	**	7
Tagliamento et al	***	-	**	5
Wang L et al	***	*	**	6
He et al	**	*	**	5
Lattenist et al	***	-	*	4
Yu et al	***	-	*	4
Wu et al	***	-	*	4

eFigure 1. Newcastle-Ottawa scale for quality assessment of the included studies.

© 2022 Khoury E et al. *JAMA Network Open.*

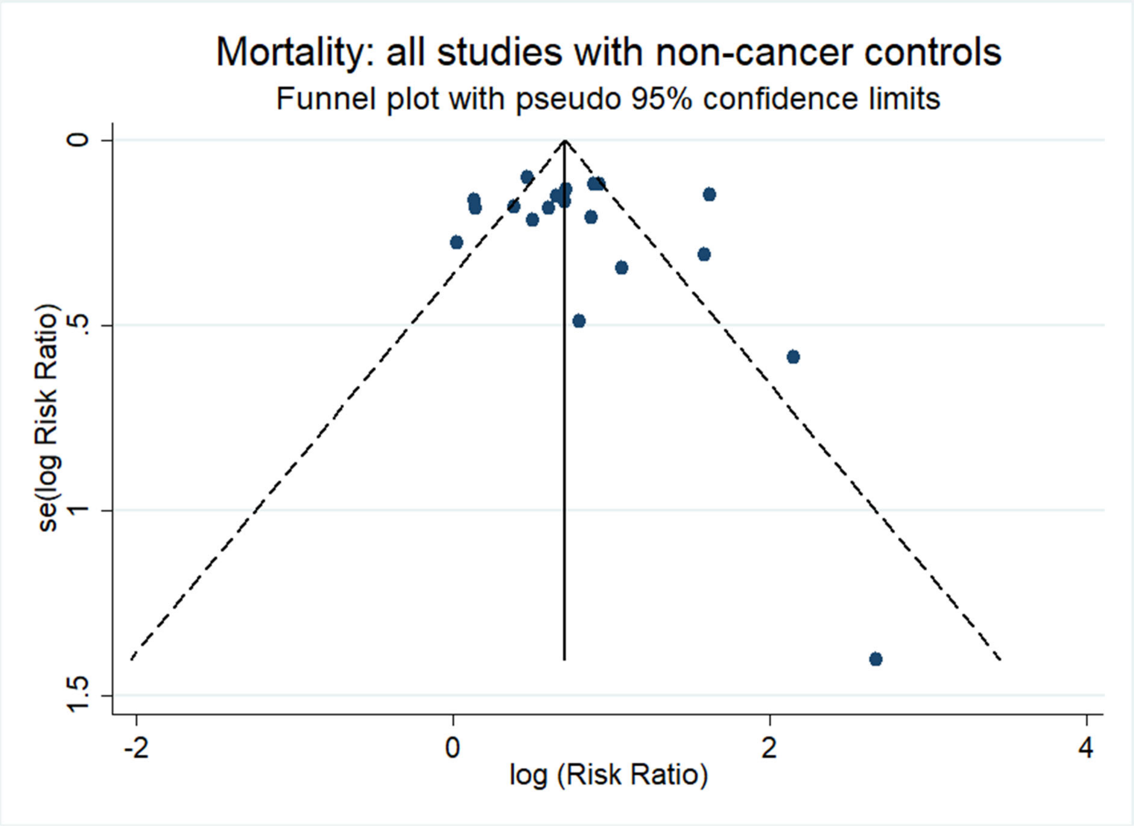

eFigure 2. Funnel plot of the main analysis of mortality in cancer patients with COVID-19 to control patients.

© 2022 Khoury E et al. *JAMA Network Open.*

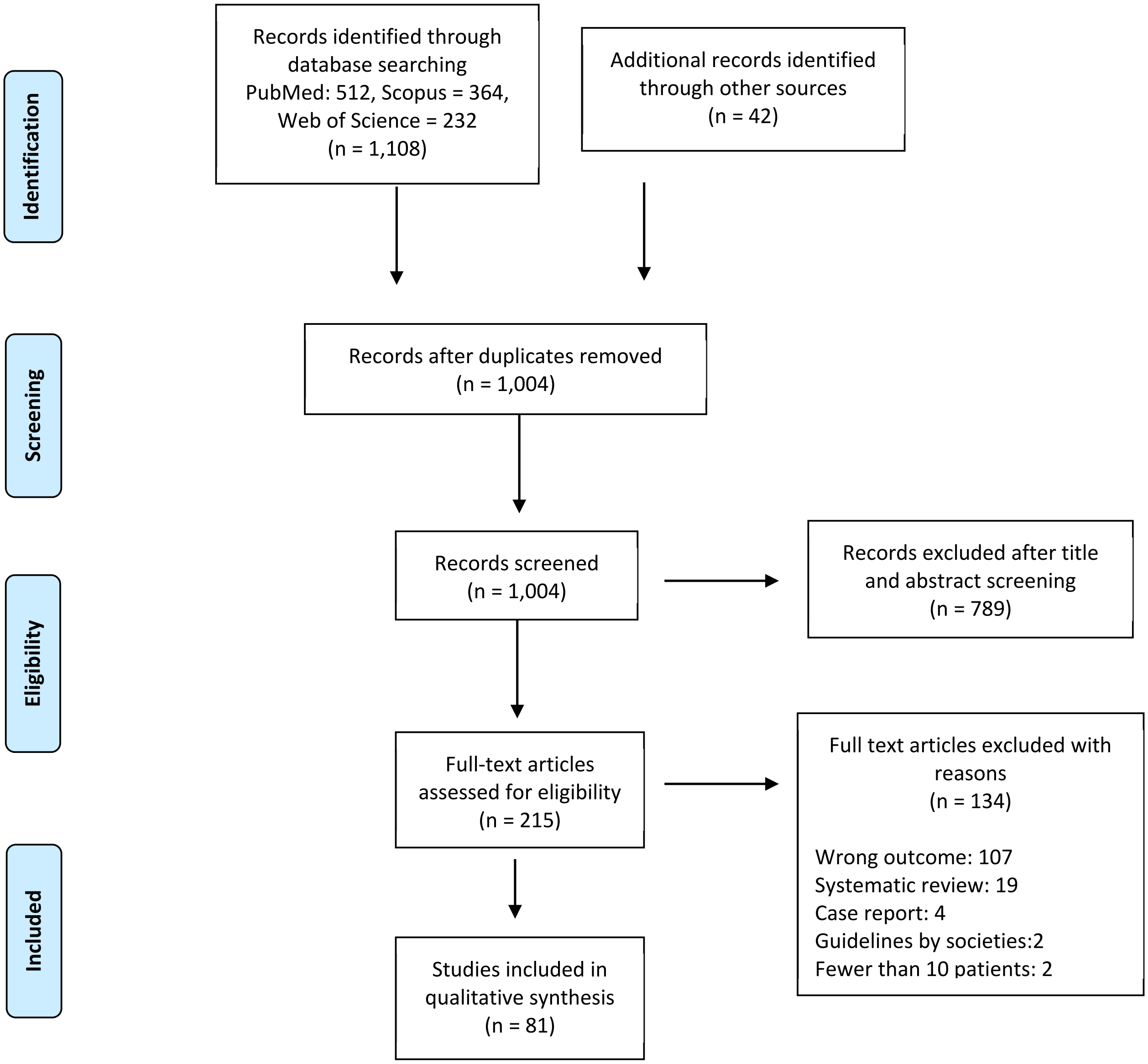

eFigure 3 PRISMA chart for study selection

© 2022 Khoury E et al. *JAMA Network Open.*

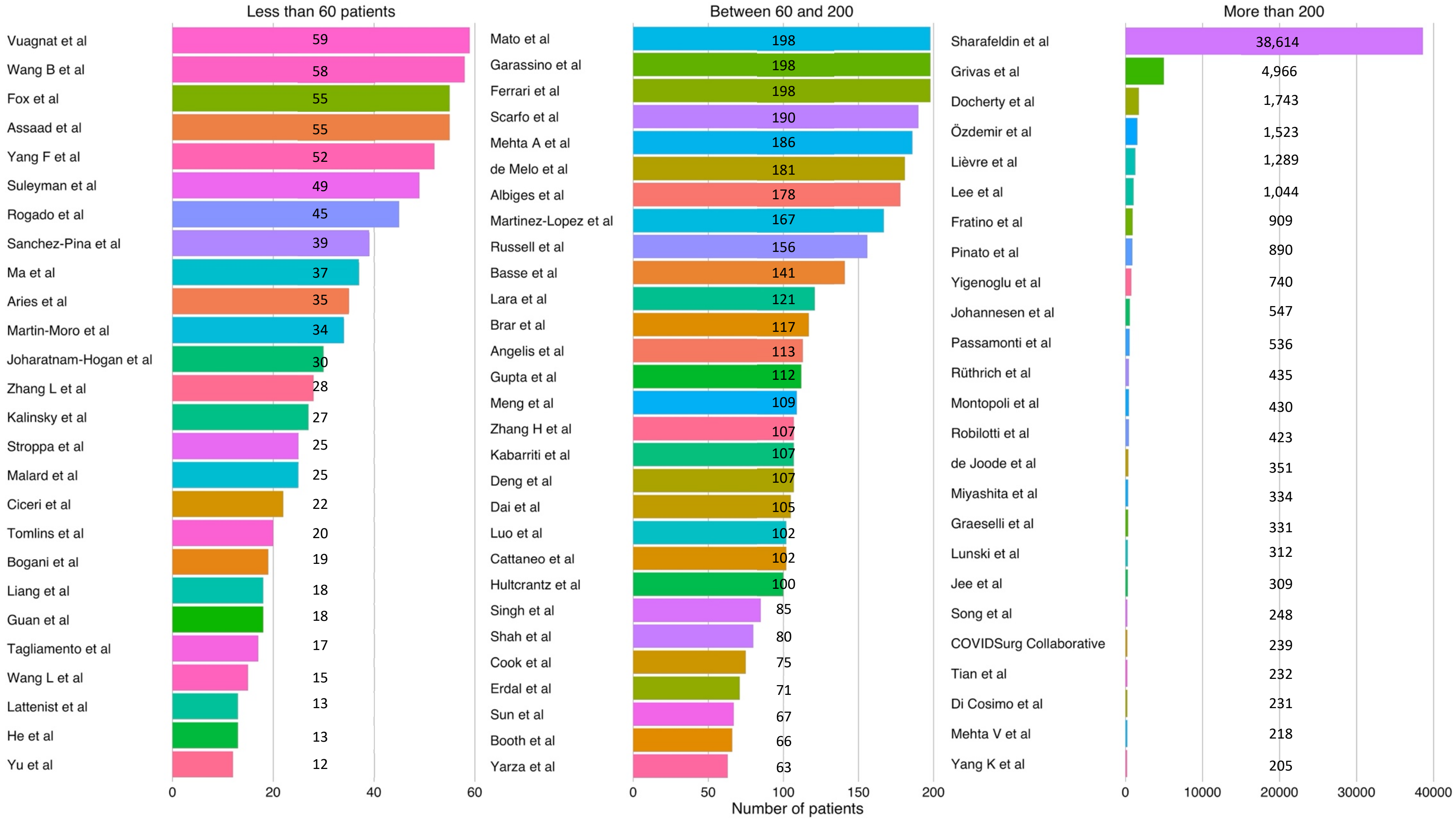

eFigure 4. Number of Cancer Patients Across 81 Studies, a Total of 61,532 Patients

© 2022 Khoury E et al. *JAMA Network Open*.

Publication	Country	Study type	Time period	Cancer patients	Cancer type	Cancer vs non cancer cohort	Presenting symptoms	Co-morbidities	Impact of cancer treatment	Severity	Outcome
Sharafeldin et al	USA	N3C Cohort, multicentre, retrospective	1st January 2020 to 25th March 2021	38,614	Any				■	■	■
Grivas et al *	Multicentre[1]	Covid-19 and Cancer Consortium registry database	17th March to 16th April 2020	4966	Any		■		■	■	■
Docherty et al	United Kingdom	Multi-centre, 208 centres, prospective	6th February to 19th April 2020	1743	Any						■
Özdemir et al	Turkey	Multi centre, retro-prospective	11th March to 20th May 2020	1523	Solid cancers			■	■	■	■
Lièvre et al	France	Multi-centre, retro-prospective	4th April to 11th June 2020	1289	Solid cancers		■	■	■	■	■
Lee et al *	United Kingdom	Multi-centre, prospective	18th March to 8th May 2020	1044	Any		■	■	■	■	■
Fratino et al	Italy	Multi centre, retrospective	Up to 30th March 2020	909	Any						■
Pinato et al	Multicentre[2]	Multi-centre, 19 centres, retrospective	26th February to 1st April 2020	890	Any		■	■	■		■
Yigenoglu et al	Turkey	Multi centre, retrospective	11th March to 22nd June 2020	740	Haematological malignancies	■		■		■	■
Johannesen et al	Norway	Multi-centre, retrospective	1st January to 31st May 2020	547	Any	■					■
Passamonti et al	Italy	Multi-centre, retrospective	25th February to 18th May 2020	536	Haematological malignancies		■	■		■	■

© 2022 Khoury E et al. *JAMA Network Open.*

Publication	Country	Study type	Time period	Cancer patients	Cancer type	Cancer vs non cancer cohort	Presenting symptoms	Co-morbidities	Impact of cancer treatment	Severity	Outcome
Rüthrich et al	Germany	Multi centre, retrospective	16th March to 31st August 2020	435	Any	■	■	■		■	■
Montopoli et al	Italy	Multi-centre, 68 centres, retrospective	Up to 1st April 2020	430	Any (male patients only)	■			■	■	■
Robilotti et al	USA	Single centre, retrospective	10th March to 7th May 2020	423	Any		■	■	■	■	■
de Joode et al	The Netherlands	Multi-centre, retrospective	27th March to 4th May 2020	351	Any			■	■	■	■
Miyashita et al	USA	Single centre, retrospective	1st March to 6th April 2020	334	Any	■					■
Graeselli et al	Italy	Multi-centre, retrospective	20th February to 22nd April 2020	331	Any						■
Lunski et al	USA	Multi centre, retrospective	1st March to 30th April 2020	312	Any	■		■	■		■
Jee et al	USA	Single centre, retrospective	8th March to 31st March 2020	309	Any		■	■	■	■	■
Song et al	China	Multi centre, 33 centres, retrospective	1st January to 25th March 2020	248	Any		■	■	■	■	■
COVIDSurg Collaborative	24 countries[3]	Multi centre, prospective	1st January to 31st March 2020	239	Any						■
Tian et al	China	Multi-centre, retrospective	13th January to 18th March 2020	232	Any	■	■	■	■	■	■
Di Cosimo et al	Italy	Multi centre, 26 centres, retro-prospective	15th May to 30th September 2020	231	Any		■	■	■	■	■

© 2022 Khoury E et al. *JAMA Network Open.*

Publication	Country	Study type	Time period	Cancer patients	Cancer type	Cancer vs non cancer cohort	Presenting symptoms	Co-morbidities	Impact of cancer treatment	Severity	Outcome
Mehta V et al	USA	Single centre, retrospective	18th March to 8th April 2020	218	Any	■		■	■	■	■
Yang K et al	China	Multi-centre, retrospective	13th January to 18th March 2020	205	Any		■	■	■	■	■
Ferrari et al	Brazil	Multi centre, prospective	29th March to 4th July 2020	198	Any		■	■	■	■	■
Garassino et al	Multicentre[4]	Multi-centre longitudinal, prospective	26th March to 12th April 2020	198	Thoracic cancer		■	■	■		■
Mato et al	Multicentre[5]	Multi-centre, 43 centres, retrospective	17th February to 30th April 2020	198	CLL		■	■	■		■
Scarfo et al	International centres[6]	Multi-centre, retrospective	28th March to 22nd May 2020	190	CLL		■	■	■	■	■
Mehta A et al	India	Single centre, retrospective	8th June to 20th August 2020	186	Any		■	■	■	■	■
de Melo et al	Brazil	Single centre, retrospective	30th April to 26th May 2020	181	Any		■	■	■	■	■
Albiges et al	France	Single centre, retrospective	24th March to 29th April 2020	178	Any		■	■	■	■	■
Martinez-Lopez et al	Spain	Multi-centre, 73 centres, retrospective	1st March to 30th April 2020	167	Myeloma	■		■	■	■	■
Russell et al	United Kingdom	Single centre, retrospective	29th February to 12th May 2020	156	Any		■	■		■	■
Basse et al	France	Single centre, prospective	13th March to 25th April 2020	141	Any		■	■		■	■

© 2022 Khoury E et al. *JAMA Network Open.*

Publication	Country	Study type	Time period	Cancer patients	Cancer type	Cancer vs non cancer cohort	Presenting symptoms	Co-morbidities	Impact of cancer treatment	Severity	Outcome
Lara et al	USA	Multi centre, retrospective	1st March to 22nd April 2020	121	Gynaecological cancer		■	■	■	■	■
Brar et al	USA	Single centre, retrospective	3rd March to 15th May 2020	117	Any	■	■	■	■	■	■
Angelis et al	United Kingdom	Single centre, prospective	1st March to 30th April 2020	113	Any		■	■			■
Gupta et al	USA	Multi centre, 65 centres, prospective	4th March to 4th April 2002	112	Any						■
Meng et al	China	Single centre, retrospective	18th January to 27th March 2020	109	Any	■		■			■
Deng et al	China	Multi-centre, retrospective	Up to 11th February 2020	107	Any						■
Kabarriti et al	USA	Single centre, retrospective	14th March to 15th April 2020	107	Any (all prior radiotherapy to lung)				■		■
Zhang H et al	China	Multi centre, 5 centres, retrospective	5th January to 18th March 2020	107	Any		■	■	■	■	■
Dai et al	China	Multi-centre, retrospective	1st January to 24th February 2020	105	Any	■	■	■	■	■	■
Luo et al	USA	Single centre, retrospective	12th March to 6th May 2020	102	Lung cancer		■	■	■	■	■
Cattaneo et al	Italy	Multi centre, retrospective	1st March to 31st March 2020	102	Haematological malignancies	■			■		■
Hultcrantz et al	USA	Multi-centre, 5 centres, retrospective	1st March to 30th April 2020	100	Multiple myeloma			■	■		■

© 2022 Khoury E et al. *JAMA Network Open.*

Publication	Country	Study type	Time period	Cancer patients	Cancer type	Cancer vs non cancer cohort	Presenting symptoms	Co-morbidities	Impact of cancer treatment	Severity	Outcome
Singh et al	USA	Single centre, retrospective	10th March to 17th April 2020	85	Any						■
Shah et al	United Kingdom	Multi-centre, retrospective	13th March to 5th May 2020	80	Haematological malignancies	■	■	■	■	■	■
Cook et al	United Kingdom	Multicentre, prospective	Up to 18th May 2020	75	Multiple myeloma			■			■
Erdal et al	Turkey	Single centre, retrospective	15th March to 15th May 2020	71	Any		■	■	■	■	■
Sun et al	USA	Multi-centre, retrospective	Up to June 2020	67	Any	■	■	■		■	■
Booth et al	United Kingdom	Multi centre, prospective	1st March to 6th May 2020	66	Haematological malignancies				■		■
Yarza et al	Spain	Single centre, prospective	9th March to 19th April 2020	63	Any		■	■	■	■	■
Vuagnat et al	France	Single centre, prospective	13th March to 25th April 2020	59	Breast cancer		■	■		■	■
Wang B et al	USA	Single centre, retrospective	1st March to 30th April 2020	58	Multiple myeloma		■	■	■		■
Assaad et al	France	Multi-centre, prospective	1st March to 25th April 2020	55	Any		■				■
Fox et al	United Kingdom	Single centre, retrospective	20th March to 20th April 2020	55	Haematological malignancies		■	■	■	■	■
Yang F et al	China	Retrospective study	1st January to 15th April 2020	52	Any		■	■	■	■	■
Suleyman et al	USA	Multi centre, retrospective	9th March t0 27th March 2020	49	Any					■	■

© 2022 Khoury E et al. *JAMA Network Open.*

Publication	Country	Study type	Time period	Cancer patients	Cancer type	Cancer vs non cancer cohort	Presenting symptoms	Co-morbidities	Impact of cancer treatment	Severity	Outcome
Rogado et al	Spain	Single centre, retrospective	1st February to 7th April 2020	45	Any		■	■	■	■	■
Sanchez-Pina et al	Spain	Single centre, retrospective	7th March to 7th April 2020	39	Haematological malignancies		■	■	■	■	■
Ma et al	China	Single centre, retrospective	1st January to 30th March 2020	37	Any		■			■	
Aries et al	United Kingdom	Single centre, retrospective	11th March to 11th May 2020	35	Haematological malignancies		■	■	■		■
Martin-Moro et al	Spain	Single centre, retrospective	9th March to 17th April 2020	34	Haematological malignancies		■		■	■	■
Joharatnam-Hogan et al	United Kingdom	Multi centre, 4 centres retrospective	12th March to 7th April 2020	30	Any	■	■	■	■	■	■
Zhang L et al	China	Multi-centre, retrospective	13th January to 26th February 2020	28	Any		■	■	■	■	■
Kalinsky et al	USA	Single centre, retrospective	10th March to 29th April 2020	27	Breast cancer		■	■			■
Malard et al	France	Single centre, prospective	9th March to 4th April 2020	25	Haematological malignancies		■	■		■	■
Stroppa et al	Italy	Single centre, retrospective	21st February to 18th March 2020	25	Any	■		■		■	■
Ciceri et al	Italy	Single centre, prospective	25th February to 24th March 2020	22	Any					■	■
Tomlins et al	United Kingdom	Single centre, retrospective	10th March to 30th March 2020	20	Any						■

© 2022 Khoury E et al. *JAMA Network Open.*

Publication	Country	Study type	Time period	Cancer patients	Cancer type	Cancer vs non cancer cohort	Presenting symptoms	Co-morbidities	Impact of cancer treatment	Severity	Outcome
Bogani et al	Italy	Single centre, retrospective	February to March 2020	19	Gynaecological cancer		■	■			■
Liang et al	China	Multi-centre, prospective	31st January 2020	18	Any	■		■	■		■
Guan et al	China	Multi centre, 575 hospitals, retrospective	11th December to 31st January 2020	18	Any					■	■
Tagliamento et al	Italy	Single centre, prospective	10th March to 6th April 2020	17	Any					■	■
Wang L et al	China	Single centre, retrospective	1st January to 6th February 2020	15	Any						■
He et al	China	Multi centre, retrospective	23rd January to 14th February 2020	13	Haematological malignancies	■	■	■	■	■	■
Lattenist et al	Belgium	Single centre, retrospective	13th March to 15th May 2020	13	Haematological malignancies		■	■	■		■
Yu et al	China	Single centre, retrospective	30th December to 17th February 2020	12	Any		■			■	■
Wu et al	China	Multi centre, 2 centres retrospective	9th January to 20th March 2020	11	Any		■		■	■	■

eTable 4 Overview of studies included in review.
CCC-19, COVID-19 and Cancer Consortium; CLL, chronic lymphocytic leukaemia. * Largest and most up to date study included in review. Shaded box indicates inclusion of factor. [1] USA (n=4,739), outside USA (n=227); [2] Italy (n=343), Spain (n=323), United Kingdom (n=218), Germany (n=6); [3] UK, Italy, Spain, USA, France, Ireland, Portugal, Egypt, Turkey, Belgium, Greece, Switzerland, Germany, The Netherlands, Libya, Croatia, Algeria, Azerbaijan, Denmark, Israel, Jordan, Mexico, Pakistan, Sudan; [4] Italy, Spain, France, Switzerland, The Netherlands, USA, United Kingdom, China; [5] USA, Spain, United Kingdom, Other European, Unknown.

© 2022 Khoury E et al. *JAMA Network Open.*

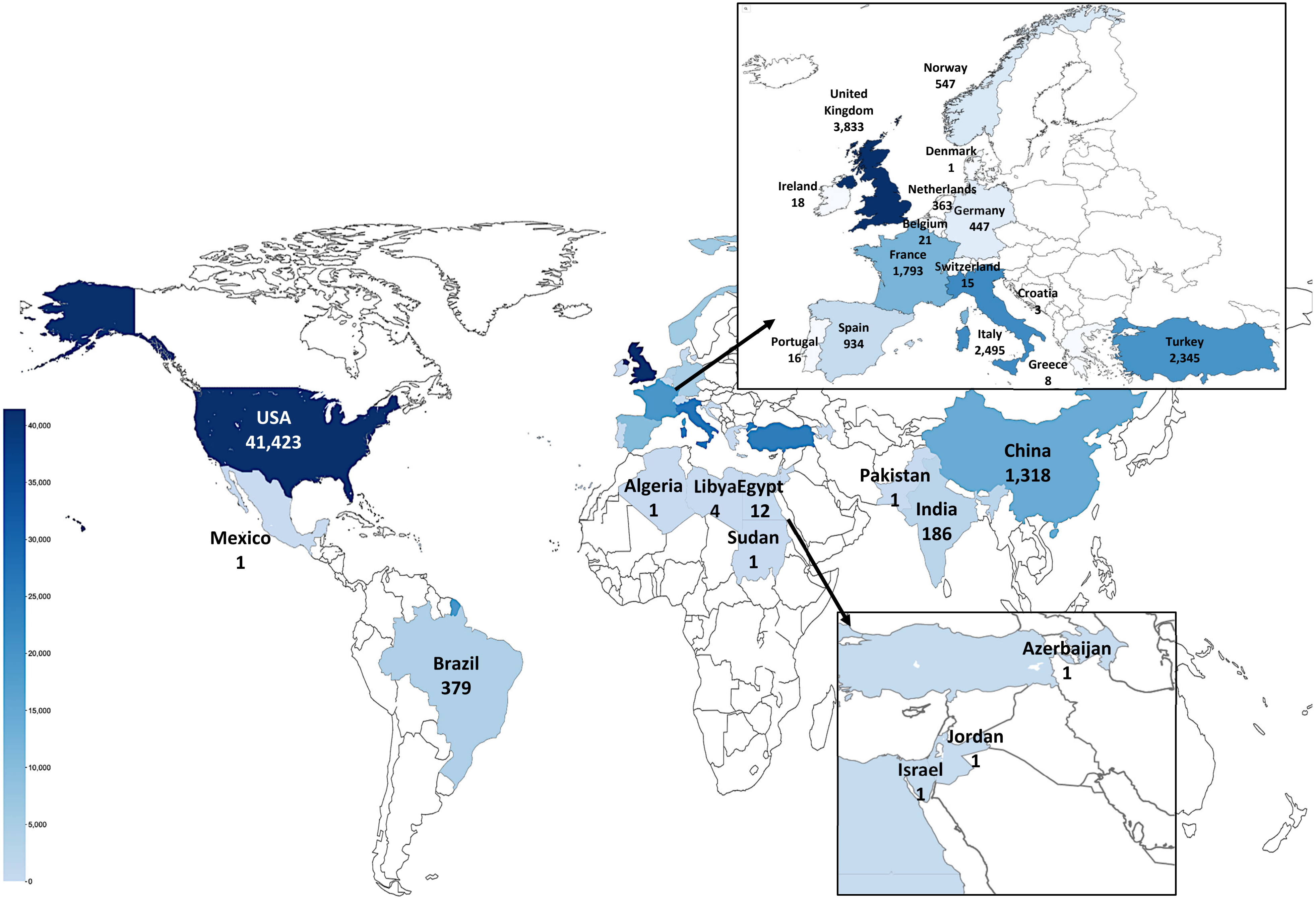

eFigure 5. Distribution of Cancer Patients Across 80 Studies

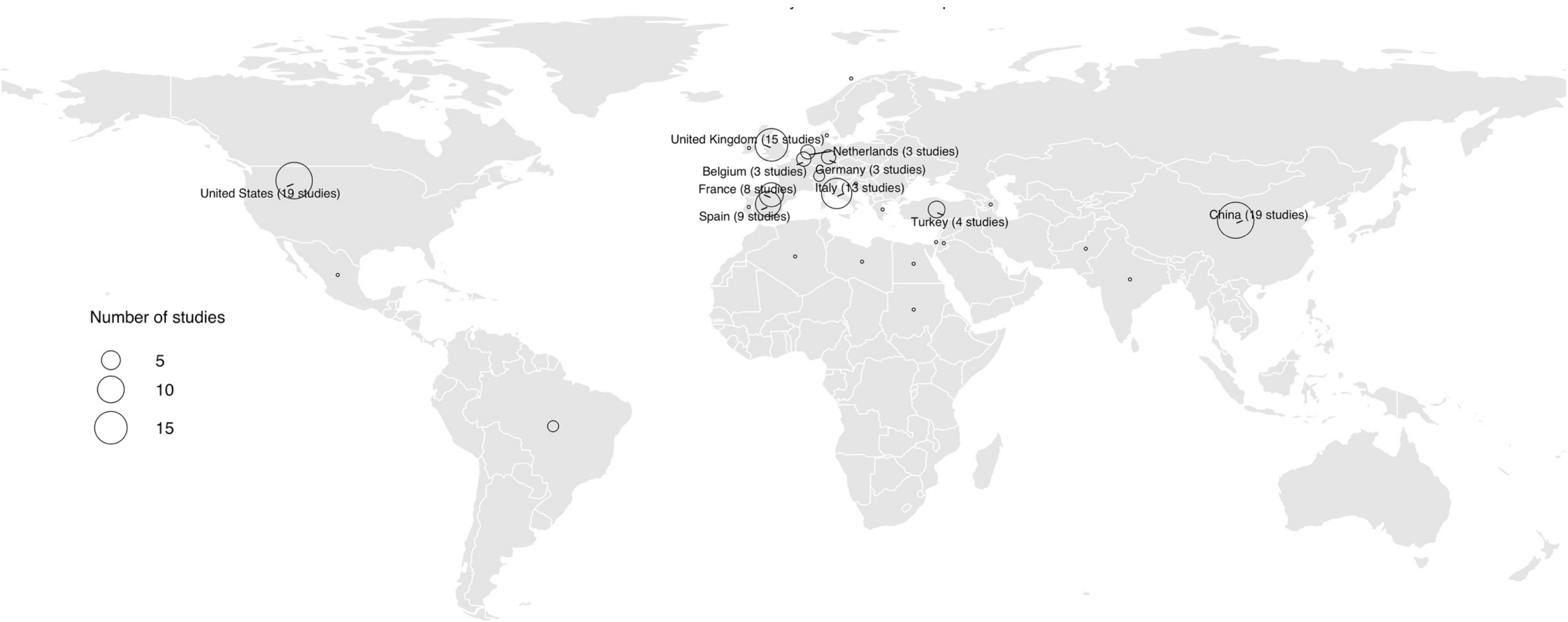

eFigure 6 Countries included across all studies.

© 2022 Khoury E et al. *JAMA Network Open*.

Country	Number of patients
**USA**	41,423
**United Kingdom**	3,833
**Italy**	2,495
**France**	1,793
**Turkey**	2.345
**China**	1,318
**Spain**	934
**Norway**	547
**Germany**	447
**Brazil**	379
**The Netherlands**	363
**India**	186
**Belgium**	21
**Ireland**	18
**Portugal**	16
**Switzerland**	15
**Egypt**	12
**Greece**	8
**Libya**	4
**Croatia**	3
**Algeria**	11
**Azerbaijan**	1
**Denmark**	1
**Israel**	1
**Jordan**	1
**Mexico**	1
**Pakistan**	1
**Sudan**	1
**Total**	**56,168**

eTable 5. Distribution of Cancer Patients by County

© 2022 Khoury E et al. *JAMA Network Open.*

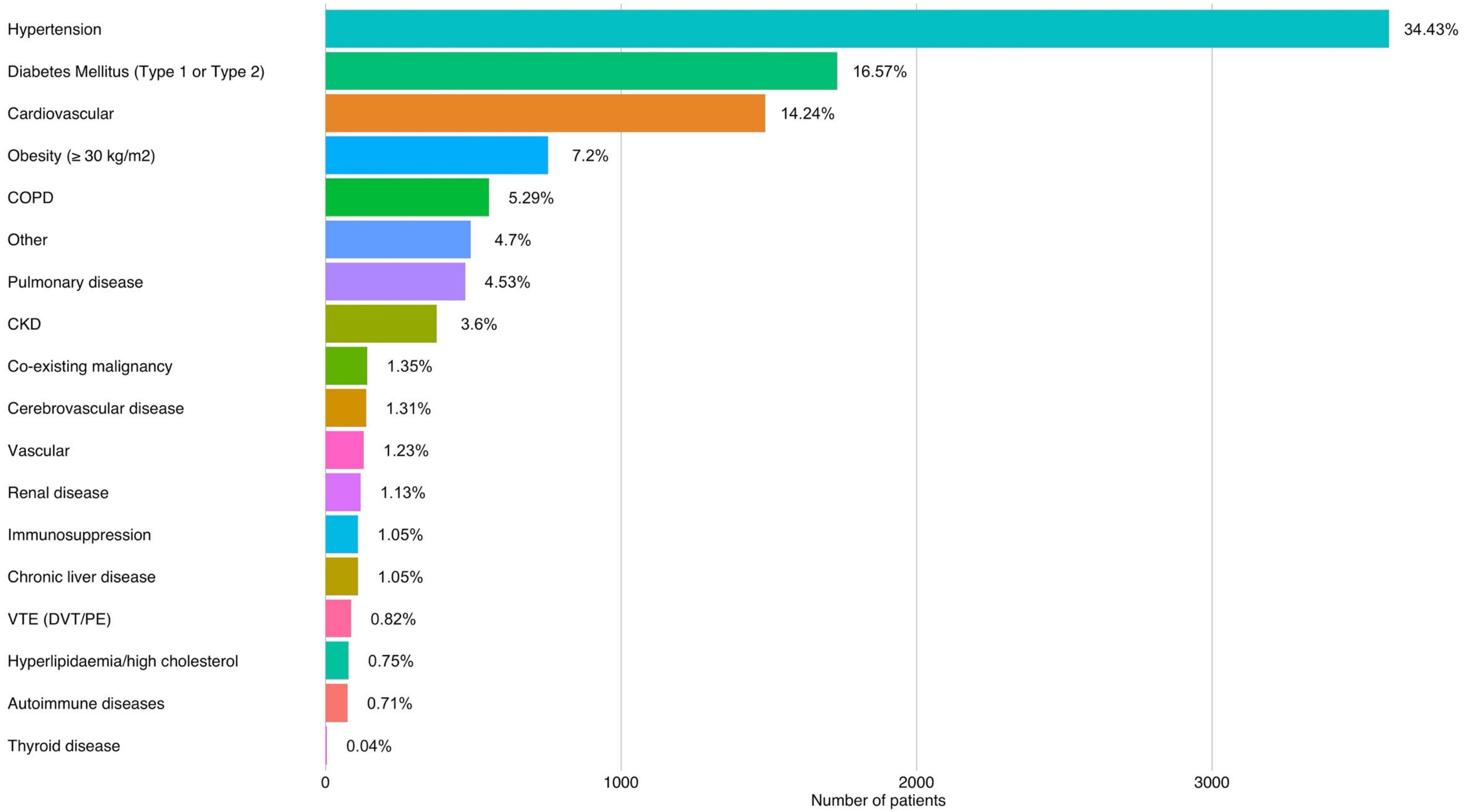

eFigure 7. Co-morbidities Experienced by Cancer Patients, Representing 21,697 Patients Across 56 Studies

© 2022 Khoury E et al. *JAMA Network Open*.

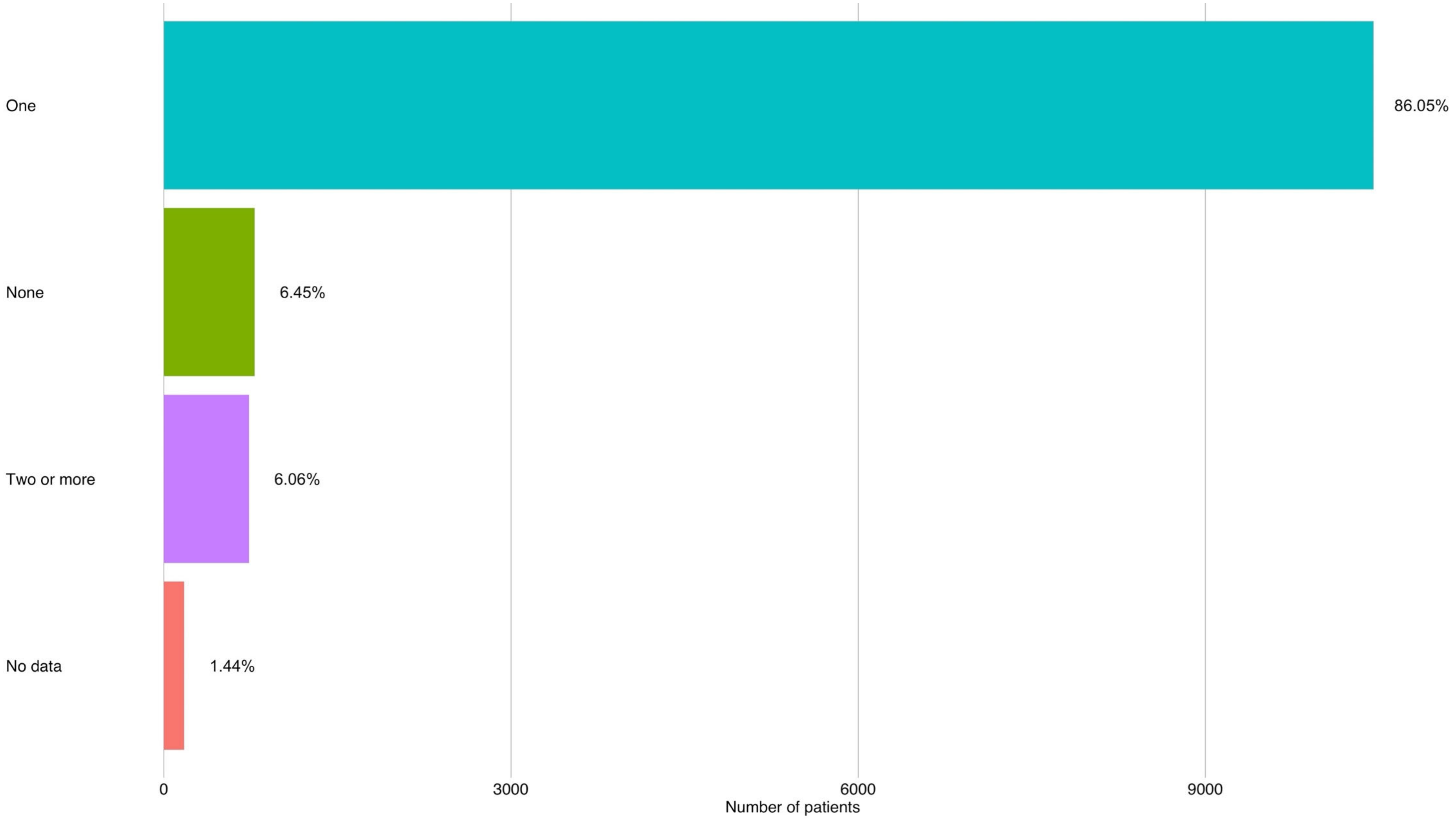

eFigure 8. Percentage of Cancer Patients Without Co-morbidities, or With One or More Co-morbidities, Where Reported

© 2022 Khoury E et al. *JAMA Network Open*.

	Hypertension	Diabetes Mellitus (Type 1 or Type 2)	Cardiovascular	COPD	Obesity (≥ 30 kg/m^2)	Pulmonary disease	CKD	Vascular	Cerebrovascular disease	Renal disease	Immunosuppression	Chronic liver disease [1]	VTE (DVT/PE)	Co-existing malignancy	Hyperlipidaemia/high cholesterol	Autoimmune diseases	Thyroid disease	Other [2]	None	No data	≥ 2 comorbidities	Total number of patients in study
**Grivas et al**		1385	1582			1091	831															**4,966**
**Özdemir et al**	391 (26)	194 (13)		106 (7)			72 (5)		69 (5)			32 (2)							528 (35)	297 (20)		**1,523**
**Lievre et al**	529 (46)	241 (21)	194 (16)	124 (12)	183 (16)																	**1,289**
**Lee et al**	343 (33)	178 (17)	145 (14)	80 (8)														336 (42)	205 (20)	175 (17)		**1,044**
**Pinato et al**	386 (43)	181 (20)	149 (17)			119 (13)	77 (9)	38 (4)	54 (6)		23 (3)	15 (2)						296 (33)			411 (46)	**890**
Yigenoglu et al	379 (51)	198 (27)	156 (21)			175 (24)																**740**
**Passamonti et al**	x	72 (13)	82 (15)			43 (8)		34 (6)	91 (17)	34 (6)		11 (2)		51 (10)				13 (2)				**536**
**Rüthrich et al**	259 (60)	104 (24)	169 (39)	39 (9)	69 (24)	42 (10)	87 (21)	34 (8)	59 (14)	25 (8)	5 (1)	11 (3)						37 (9)				**435**
**Robilotti et al**	214 (51)	84 (20)	84 (20)	36 (9)	129 (30)	43 (10)	29 (7)															**423**
**de Joode et al**		55 (16)	190 (54)	46 (13)	64 (18)									63 (18)		22 (6)						**351**
**Lunski et al**	246 (79)	116 (37)	34 (11)	35 (11)	143 (46)		80 (26)					10 (3)										**312**
**Jee et al**	120 (39)			16 (5)	92 (30)							36 (12)										**309**
**Song et al**	83 (33)	38 (15)	28 (11)	21 (8)					18 (7)													**248**
**Tian et al**	96 (41)	55 (24)	22 (9)	3 (1)			6 (3)			6 (3)			9 (4)									**232**
**Di Cosimo et al**	97 (42)	43 (19)	36 (16)			19 (8)												25 (11)	82 (36)	1 (0)	52 (23)	**231**
**Mehta V et al**	147 (67)	80 (37)	66 (30)			62 (28)	54 (25)															**218**
**Yang K et al**	67 (33)	22 (11)	16 (8)	5 (2)			4 (2)			13 (6)									99 (48)			**205**
**Ferrari et al**	90 (45)	44 (22)	23 (12)																			**198**
**Garassino et al**	93 (47)	29 (15)	40 (20)	51 (26)			15 (8)			16 (8)						6 (3)		96 (47)	32 (16)		106 (54)	**198**
**Mato et al**	101 (51)	40 (20)	39 (20)	22 (11)		12 (6)		34 (17)			78 (44)		25 (13)			20 (10)						**198**
**Scarfo et al**	78 (54)	35 (24)	42 (29)	9 (6)		21 (14)								18 (12)					45 (24)		102 (54)	**190**
**Mehta A et al**	45 (24)	34 (18)	11 (6)	2 (1)													30 (16)	5 (3)	100 (54)		39 (21)	**186**
**de Melo et al**	77 (43)	31 (17)				7 (4)				10 (6)												**181**
**Albiges et al**	65 (37)	35 (20)	21 (12)		25 (14)		10 (6)								16 (9)	9 (5)						**178**
**Martinez-Lopez et al**	67 (40)	28 (17)	35 (21)			23 (14)		32 (19)											41 (25)			**167**
**Russell et al**	74 (47)	35 (22)	29 (19)			25 (16)	30 (19)			3 (2)	4 (3)							10 (6)	43 (28)		65 (42)	**156**
**Basse et al**	48 (34)	24 (17)	21 (15)			7 (5)												6 (4)	88 (62)			**141**
**Lara et al**	69 (57)	38 (31)	8 (7)	4 (3)		10 (8)	9 (7)							8 (7)		12 (10)		8 (7)				**121**
**Brar et al**	62 (53)	38 (33)	35 (30)	9 (8)	28 (24)	6 (5)	3 (3)															**117**
**Angelis et al**	39 (35)	18 (16)	13 (12)	6 (5)														11 (10)	27 (24)			**113**
**Meng et al**	30 (28)	11 (10)	11 (10)															2 (2)				**109**
**Zhang H et al**	52 (49)	22 (21)	14 (13)	5 (5)												4 (4)			35 (33)			**107**
**Dai et al**	30 (29)	7 (7)	12 (11)				6 (6)			7 (7)			5 (5)									**105**
**Luo et al**	57 (56)	27 (27)	7 (7)	24 (24)	30 (29)	28 (27)						18 (18)										**102**
**Hultcrantz et al**	55 (55)	18 (18)	23 (23)	1 (1)		2 (2)	3 (3)						3 (3)		20 (20)			3 (3)	28 (28)		37 (37)	**100**
**Shah et al**	21 (50)	9 (21)	5 (12)	3 (7)		5 (12)																**80**

© 2022 Khoury E et al. *JAMA Network Open.*

**Cook et al**	31 (41)																				**75**
**Erdal et al**	34 (48)	25 (35)	6 (9)	20 (28)			7 (10)		6 (9)												**71**
**Sun et al**	38 (57)	22 (33)	11 (16)		12 (18)	14 (21)			3 (5)		5 (8)							8 (12)				**67**
**Yarza et al**	33 (52)	11 (17)	12 (19)			14 (22)	5 (8)					13 (21)										**63**
**Vuagnat et al**	21 (36)	10 (17)	8 (5)		10 (17)	2 (3)												3 (5)	39 (51)			**59**
**Wang B et al**	37 (64)	16 (28)	39 (67)		22 (37)	12 (21)	14 (24)								36 (62)			18 (31)				**58**
**Fox et al**	20 (37)	11 (20)	3 (6)	1 (2)			7 (13)															**55**
**Yang F et al**	17 (33)	7 (21)	5 (15)	4 (8)			1 (2)			3 (6)			4 (8)				1 (2)		19 (37)			**52**
**Rogado et al**	23 (51)	13 (29)	4 (9)	13 (29)	6 (13)		3 (7)															**45**
**Sanchez-Pina et al**	19 (49)	7 (18)	6 (15)	2 (5)								4 (10)							17 (44)			**39**
**Aries et al**	10 (29)	5 (14)	8 (23)	2 (6)		2 (6)	5 (14)	5 (14)		1 (3)		3 (9)		1 (3)				4 (11)				**35**
**Joharatname Hogan et al**	11 (42)	11 (42)	6 (20)			7 (23)	1 (3)					1 (3)			6 (20)	1 (3)		9 (30)	2 (7)		15 (58)	**30**
**Zhang L et al**	4 (14)	4 (14)	3 (11)	1 (4)						2 (7)									18 (64)			**28**
**Kalinsky et al**	15 (56)	6 (22)	3 (11)			6 (22)													9 (33)			**27**
**Malard et al**	17 (68)	6 (24)	1 (4)	2 (8)	8 (32)		5 (20)						6 (24)	5 (20)					2 (8)			**25**
**Stroppa et al**	16 (64)	8 (32)		7 (28)															6 (24)			**25**
**Bogani et al**			5 (26)				1 (5)										3 (16)		10 (53)			**19**
**Liang et al**	2 (11)	2 (11)		1 (6)			1 (6)						1 (6)						14 (78)			**18**
**He et al**			3 (23)										3 (23)									**13**
**Lattenist et al**	2 (15)	1 (8)			1 (8)	3 (23)		3 (23)										4 (31)	5 (39)			**13**
**Total**	**4,860**	**3,734**	**3,465**	**700**	**822**	**1800**	**1366**	**180**	**300**	**125**	**115**	**154**	**56**	**146**	**78**	**74**	**34**	**894**	**1,494**	**473**	**827**	**21,697**

eFigure 9 Summary of reported co-morbidities.
CDK, chronic kidney disease; DVT, deep venous thrombosis; PE, pulmonary embolism; VTE, venous thromboembolism.

© 2022 Khoury E et al. *JAMA Network Open*.

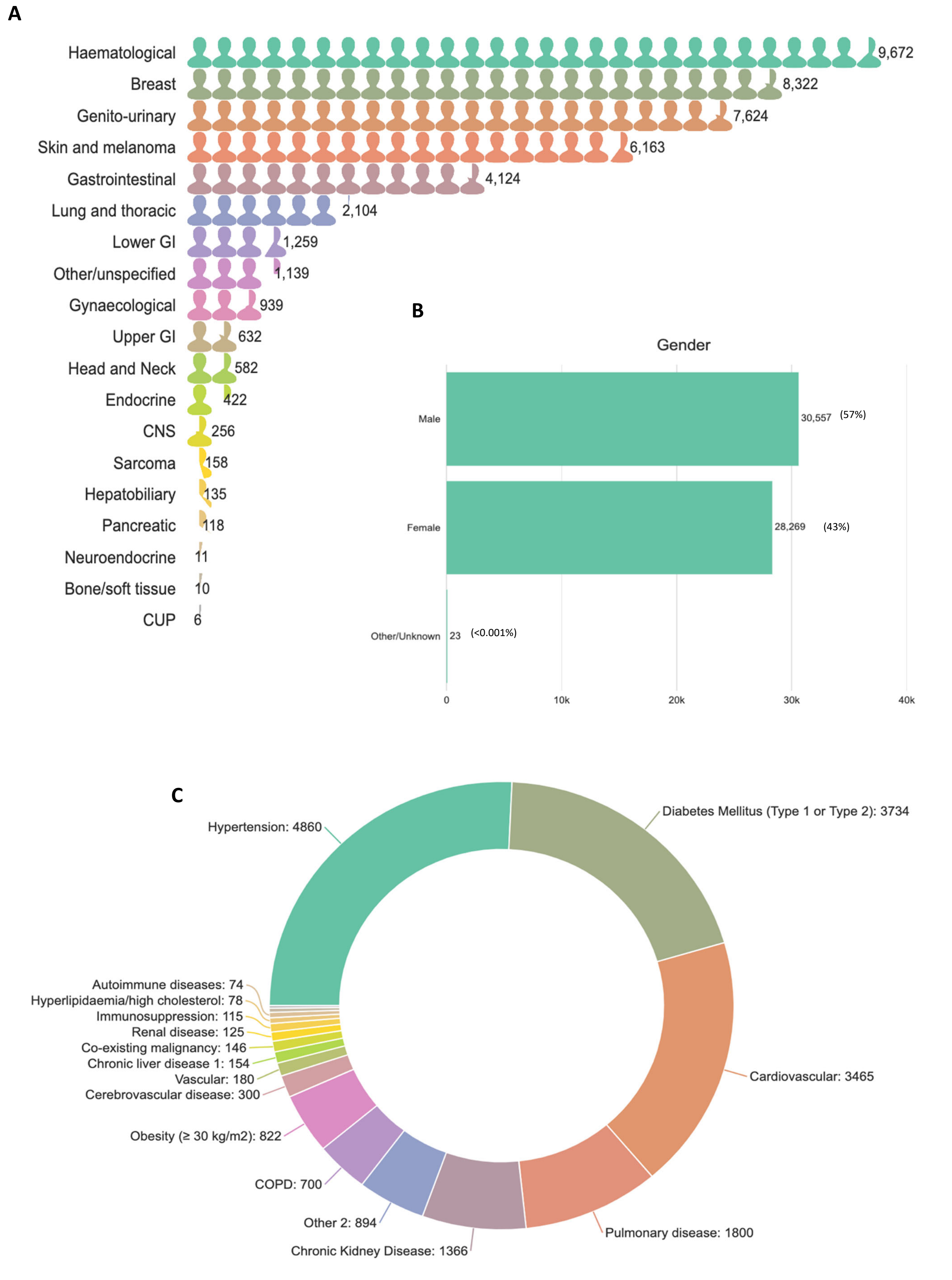

eFigure 10 Demographic and presenting symptoms.
(A) Tumour prevalence: includes 43,676 patients from 68 studies (B) Sex distribution across studies: figure includes 58,849 patients from 70 studies. (C) Co-morbidities: figure includes 21,697 patients from 56 studies.

© 2022 Khoury E et al. *JAMA Network Open.*

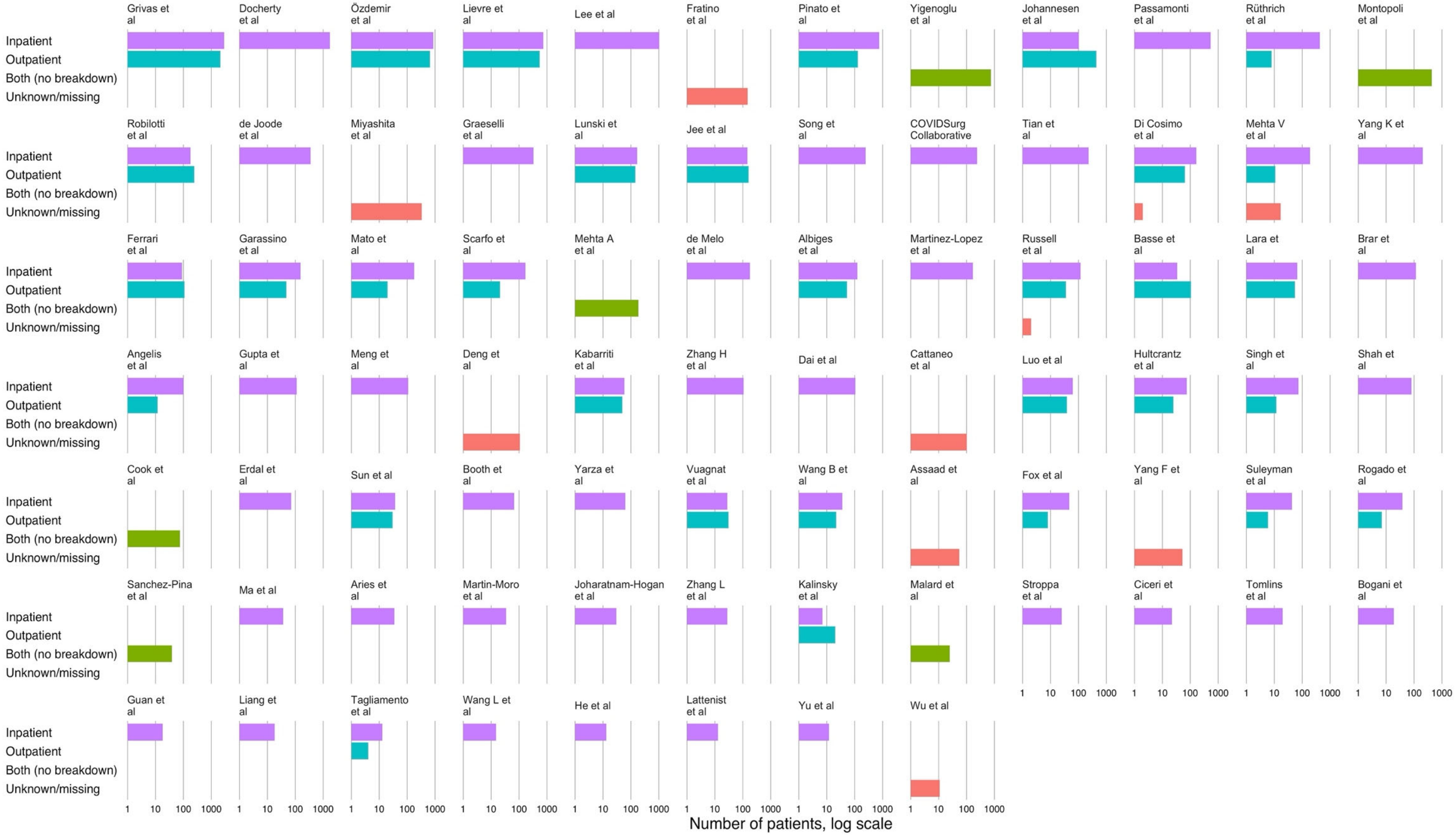

eFigure 11. Setting of Care Across 80 Studies, Representing 22,918 Cancer Patients

© 2022 Khoury E et al. *JAMA Network Open*.

Publication	Total number of patients	Inpatient	Outpatient	Both (no breakdown)	Unknown/missing data	Possible nosocomial infection	Hospitalised at time of infection
**Sharafeldin et al**	**38,614**	**19,515**	**19,099**				
**Grivas et al**	**4966**	2872	2094			N	
**Docherty et al**	**1743**	1743	-	-		N	
**Özdemir et al**	**1523**	862	661				
**Lievre et al**	**1289**	734	555			N	
**Lee et al**	**1044**	1044	-	-		N	
**Fratino et al**	**909**				150	N	
**Pinato et al**	**890**	760	130	-		155	
**Yigenoglu et al**	**740**			740			
**Johannesen et al**	**547**	104	443				
**Passamonti et al**	**536**	536	-	-		N	
**Rüthrich et al**	**435**	427	8				
**Montopoli et al**	**430**	-	-	430		N	
**Robilotti et al**	**423**	180	243	-		N	
**de Joode et al**	**351**	351	-	-		N	
**Miyashita et al**	**334**	-	-	-	334	N	
**Graeselli et al**	**331**	331		-		N	
**Lunski et al**	**312**	166	146	-		N	
**Jee et al**	**309**	147	162	-		N	
**Song et al**	**248**	248	-	-		N	
**COVIDSurg Collaborative**	**239**	239	-	-		N	
**Tian et al**	**232**	232	-	-		N	
**Di Cosimo et al**	**231**	165	64		2		
**Mehta V et al**	**218**	190	11	-	17	37/61	
**Yang K et al**	**205**	205	-	-		N	
**Ferrari et al**	**198**	88	110				
**Garassino et al**	**198**	152	48	-		13/24	
**Mato et al**	**198**	178	20	-		N	
**Scarfo et al**	**190**	169	21	-		N	
**Mehta A et al**	**186**			186			
**de Melo et al**	**181**	181	-	-		83	
**Albiges et al**	**178**	125	53	-		31	
**Martinez-Lopez et al**	**167**	167	-	-		N	
**Russell et al**	**156**	118	36	-	2	N	

© 2022 Khoury E et al. *JAMA Network Open.*

Publication	Total number of patients	Inpatient	Outpatient	Both (no breakdown)	Unknown/missing data	Possible nosocomial infection	Hospitalised at time of infection
**Basse et al**	**141**	34	107	-		N	34
**Lara et al**	**121**	66	55	-		N	
**Brar et al**	**117**	117	-	-			
**Angelis et al**	**113**	101	12	-		Not clear	14
**Gupta et al**	**112**	112	-	-		No	
**Meng et al**	**109**	109	-	-		N	
**Deng et al**	**107**	-	-	-	107	N	
**Kabarriti et al**	**107**	58	49	-		N	
**Zhang H et al**	**107**	107		-		N	
**Dai et al**	**105**	105		-		N	
**Luo et al**	**102**	63	39	-		N	
**Cattaneo et al**	**102**	-		-	102	N	
**Hultcrantz et al**	**100**	75	25	-		N	
**Singh et al**	**85**	73	12	-		N	
**Shah et al**	**80**	80	-	-			
**Cook et al**	**75**	-	-	75		N	
**Erdal et al**	**71**	71	-	-			
**Sun et al**	**67**	37	30				
**Booth et al**	**66**	66	-	-		N	
**Yarza et al**	**63**	63	-	-		N	
**Vuagnat et al**	**59**	28	31	-		9	
**Wang B et al**	**58**	36	22	-		N	
**Assaad et al**	**55**	-		-	55	N	
**Fox et al**	**55**	47	8	-		4	
**Yang F et al**	**52**	-		-	52	N	
**Suleyman et al**	**49**	43	6	-	-	N	
**Rogado et al**	**45**	38	7	-	-	P	
**Sanchez-Pina et al**	**39**	-	-	39	-	1	1
**Ma et al**	**37**	37	-	-	-	N	
**Aries et al**	**35**	35	-	-	-	N	
**Martin-Moro et al**	**34**	34	-	-	-	N	
**Joharatnam-Hogan et al**	**30**	30	-	-	-	1	
**Zhang L et al**	**28**	28	-	-	-	8	
**Kalinsky et al**	**27**	7	20	-	-	N	

© 2022 Khoury E et al. *JAMA Network Open.*

Publication	Total number of patients	Inpatient	Outpatient	Both (no breakdown)	Unknown/missing data	Possible nosocomial infection	Hospitalised at time of infection
**Malard et al**	**25**			25	-	10 possibly	6
**Stroppa et al**	**25**	25			-	N	
**Ciceri et al**	**22**	22			-	N	
**Tomlins et al**	**20**	20			-	N	
**Bogani et al**	**19**	19			-	N	
**Liang et al**	**18**	18			-	N	
**Guan et al**	**18**	18			-	P	
**Tagliamento et al**	**17**	13	4		-	N	
**Wang L et al**	**15**	15			-	N	
**He et al**	**13**	13			-	N	
**Lattenist et al**	**13**	13			-	1	
**Yu et al**	**12**	12			-	N	
**Wu et al**	**11**				11	N	

eTable 6. Inpatient vs. outpatient care across 73 studies as well as possible or probable nosocomial infection.

© 2022 Khoury E et al. *JAMA Network Open.*

Cancer type	Number of patients	% of total
**Haematological[1]**	9,672	22.14%
**Breast**	8,322	19.05%
**Genito-urinary[2]**	7,624	17.46%
**Skin and melanoma**	6,163	14.11%
**Gastrointestinal**	4,124	9.44%
**Lung and thoracic[3]**	2,104	4.82%
**Lower Gastrointestinal[4]**	1,259	2.88%
**Other/unspecified**	1,139	2.61%
**Gynaecological**	939	2.15%
**Upper Gastrointestinal**	632	1.45%
**Head and Neck**	582	1.33%
**Endocrine**	422	0.97%
**Central Nervous System**	256	059%
**Sarcoma**	158	0.36%
**Hepatobiliary**	135	0.31%
**Pancreatic**	118	0.27%
**Neuroendocrine**	11	0.03%
**Bone/soft tissue**	10	0.02%
**Cancer of Unknown Primary**	6	0.01%
	**43,676**	

eTable 7. Tumour prevalence across studies included.
[1] acute myeloid leukaemia, chronic lymphocytic leukaemia, multiple myeloma, lymphoma, myelodysplastic syndrome, histiocytosis. [2] NSCLC, SCLC, thymoma, carcinoid, malignant pleural mesothelioma, thymic. [3] Kidney, bladder, adrenal, urothelial, testis, prostate, penile. [4] Colorectal and anal.

© 2022 Khoury E et al. *JAMA Network Open.*

	Haematological	Breast	Genito-urinary	Lung and thoracic	Other/unspecified	Gynaecological	Gastrointestinal	Lower GI	Head and Neck	Upper GI	Hepatobiliary	Skin and melanoma	CNS	Sarcoma	Endocrine	Pancreatic	Bone/soft tissue	CUP	Neuroendocrine	Total
**Sharafeldin et al**	4,749	5,482	4,738				3,413					5,743								**24,125**
**Grivas et al [1]**	1,097	967	1,176	409	54	322	108	404	151	266		183	78	110	235					**4966**
**Özdemir et al**		302	313	157	340			165				45			140					**1523**
**Lievre et al**		173	65	311	6	79	73	185	104	92	34	14	65			86				**1287**
**Lee et al**	227	143	195	122		56	95	124	33				25	16	4		4			**1044**
**Pinato et al**	137	162	132	119	52	41	105		29	40	45	28								**890**
**Yigenoglu et al**	740																			**740**
**Johannesen et al**		85	140	13		33				73		98	20		20					**547**
**Passamonti et al**	536																			**536**
**Rüthrich et al**	124	21		36		39	60													**435**
**Montopoli et al**	47		193	13	112			65												**430**
**Robilotti et al**	102	86	26	35	137			37												**423**
**de Joode et al**	112	47	51	51	23	11		31		6		8				4			7	**351**
**Miyashita et al**		57	74	23	164			16												**334**
**Lunski et al**	61	70	74	26		13	32		8			2	2		23					**312**
**Jee et al**	76	55	33	30		21	4	28	13	5	6	1	5	14		16	1		1	**309**
**Song et al**		37	21	62		10		33	41	29	14					1				**248**
**Tian et al**	12	31	50	23		15		27	31	24	10	2	3	2		2				**232**
**Di Cosimo et al**	14	65	25	42	1	5		26	9	38		3	3							**231**
**Mehta V et al**	54	28	46	11		13		21	8	8	7	3	8			3	5		3	**218**
**Yang K et al**	22	40	14	24		21		28	23	19	8	2	2	2						**205**
**Ferrari et al**	31	58	29	16	24	11	29													**198**
**Garassino et al**				198																**198**
**Mato et al**	198																			**198**
**Scarfo et al**	190																			**190**
**Mehta A et al**	33	19		17	36		40		33			6	2							**186**
**de Melo et al**	35	40	17	7		22		14	11	6	2	4	15	4		2		2		**181**
**Albiges et al**	30	32	20	17	10	23	21		22			11								**178**
**Martinez-Lopez et al**	167																			**167**
**Russell et al**	28	24	34	17	10	11	21						11							**156**
**Basse et al**	19	57	2	18	6	12	11		8				2	6						**141**
**Lara et al**						121														**121**
**Brar et al**	42	15	22		12	6	19		1											**117**
**Angelis et al**	18	18	12	15		6	32		4			3	4	1						**113**
**Meng et al**	16			17	76															**109**
**Kabarriti et al 2**	4	28	27	14	23	7			6											**109**

© 2022 Khoury E et al. *JAMA Network Open.*

**Zhang H et al**	9	10	20	21			20		17		3		6			1				**107**
**Dai et al**	9	11		22	27	6	13		11	6										**105**
**Luo et al**				102																**102**
**Cattaneo et al**	102																			**102**
**Hultcrantz et al**	100																			**100**
**Singh et al**	22	12	23	9			9		2			4	1	2				1		**85**
**Shah et al**	80																			**80**
**Cook et al**	75																			**75**
**Erdal et al**	12	11	12	7	1		19		5			2	2							**71**
**Booth et al**	66																			**66**
**Yarza et al**		10	9	17		2		10	4	6			1	1				3		**63**
**Vuagnat et al**		59																		**59**
**Wang B et al**	58																			**58**
**Fox et al**	55																			**55**
**Yang F et al**		9	2	10	7	4		13	3	2	2									**52**
**Rogado et al**		6	5	17	11			6												**45**
**Sanchez-Pina et al**	39																			**39**
**Ma et al**		7		8	6	5		11												**37**
**Aries et al**	35																			**35**
**Martin-Moro et al**	34																			**34**
**Joharatnam-Hogan et al**	1	4	9	5				5	1	1	1	1				2				**30**
**Zhang L et al**		3	3	7		3		2	3	5	2									**28**
**Kalinsky et al**		27																		**27**
**Malard et al**	25																			**25**
**Stroppa et al**	2	2	6	8	1					6										**25**
**Bogani et al**						19														**19**
**Liang et al**	1	3	4	5				4	1											**18**
**Tagliamento et al**		5	1	9				1					1							**17**
**He et al**	13																			**13**
**Lattenist et al**	13																			**13**
**Yu et al**		1	1	7				2								1				**12**
**Wu et al**				7		2		1			1									**11**
**Total**	**9,672**	**8,322**	**7,624**	**2,104**	**1,139**	**939**	**4,124**	**1,259**	**582**	**632**	**135**	**6,163**	**256**	**158**	**422**	**118**	**10**	**6**	**11**	**43676**

eFigure 12. Tumour Type Breakdown Across 68 Studies, Where Reported
[1]107 patients reported as multi-cancers; [2]Patients had two primary tumours and so were counted twice. CNS, central nervous system; CUP, cancer of unknown primary; GI, gastrointestinal.

© 2022 Khoury E et al. *JAMA Network Open.*

eResults. Presenting Symptoms of SARS-CoV-2 Infection, Radiological Findings, Ethnicity and Stage of Malignancy and Outcome

Presenting symptoms of SARS-CoV-2 infection

Fifty-three (65%) of the 81 studies, involving 9,196 patients, reported presenting symptoms. The most commonly reported symptoms were fever, cough, dyspnoea, fatigue/malaise, diarrhoea, and myalgia (eFigure 11). The time from onset of symptoms to hospitalisation ranged between 1-40 days, where reported [1-7].

Radiological findings

Radiological findings of thoracic imaging based on chest radiograph, CT scanning or a combination of chest radiograph and CT scan in cancer patients with COVID-19 were available in 28 studies (n=3,650 patients) [2-4,7-31]. A total of 19 (26%) studies (n=3,086 patients) provided details of radiological changes (eTable 8) [2,3,7,8,10-12,14,15,17,20-24,26,27,29,30]. CT features of ground glass opacity, patchy shadows, fibrous stripes, pleural thickening, bilateral involvement, and tumour pulmonary involvement in cancer patients were associated with worsening severity of COVID-19 disease [3,7,8,21,26,29].

Ethnicity

12 studies looked at association of ethnicity in cancer patients and outcomes, of which 8 found that African-American patients had more severe COVID-19 and increased mortality compared to white patients on both unadjusted analysis [5,6,11,32,33] and multivariable anaylsis[20,34,35]. Two studies found non-white patients to be significantly associated with hospitalisation and severe illness compared to white race [14,40]; and Asian ethnicity was

associated with increased mortality [HR 3.73; 95% CI: 1.28-10.91] in an adjusted analysis [28] While 10 studies did not find a significant association between ethnicity and poor outcome[1,18,34,36-41]. One of these studies found that hospitalised black cancer patients had lower mortality rates in adjusted analysis (OR 0.72, 95% CI 0.53-0.98) compared to non-white Hispanic patients [42]. Hispanic patients did not have increased risk of mortality compared to non-Hispanic patients [41]. Non-white race was found to be significantly associated with hospitalisation and severe illness compared to white race [6,20]. Asian ethnicity was associated with increased mortality [HR 3.73; 95% CI: 1.28-10.91] in adjusted analysis [43].

Stage of malignancy and outcome

There was variability with regards to the reported effect of stage of malignancy on outcomes with COVID-19. In unadjusted analysis, 8 studies have reported stage IV disease (of solid cancers) to be associated with significantly higher risk of severe events and mortality compared to patients with stage I, II or III disease [2,3,8,25,29,44-46]. However, five studies found no association with metastatic primary disease late stage of disease and rates of hospitalisation or mortality [1,22,26,36,47]. A study of 181 patients in Brazil found that those with 2 or more metastatic sites had an overall increased risk of death [26.]

eReferences

1. Garassino MC, Whisenant JG, Huang L-C, et al. COVID-19 in patients with thoracic malignancies (TERAVOLT): first results of an international, registry-based, cohort study. *The Lancet Oncology* 2020; **21**(7): 914-22.
2. Pinato DJ, Zambelli A, Aguilar-Company J, et al. Clinical portrait of the SARS-CoV-2 epidemic in European cancer patients. *Cancer Discov* 2020; **10**(10): 1465-74.
3. Yang KY, Sheng YH, Huang CL, et al. Clinical characteristics, outcomes, and risk factors for mortality in patients with cancer and COVID-19 in Hubei, China: a multicentre, retrospective, cohort study. *Lancet Oncology* 2020; **21**(7): 904-13.

4. Albiges L, Foulon S, Bayle A, et al. Determinants of the outcomes of patients with cancer infected with SARS-CoV-2: results from the Gustave Roussy cohort. *Nature Cancer* 2020; **1**(10): 965-75.
5. Lara OD, O'Cearbhaill RE, Smith MJ, et al. COVID-19 outcomes of patients with gynecologic cancer in New York City. *Cancer* 2020; **126**(19): 4294-303.
6. Wang B, Van Oekelen O, Mouhieddine TH, et al. A tertiary center experience of multiple myeloma patients with COVID-19: Lessons learned and the path forward. *Journal of Hematology and Oncology* 2020; **13**(1).
7. Zhang L, Zhu F, Xie L, et al. Clinical characteristics of COVID-19-infected cancer patients: a retrospective case study in three hospitals within Wuhan, China. *Ann Oncol* 2020; **31**(7): 894-901.
8. Tian JB, Yuan XL, Xiao J, et al. Clinical characteristics and risk factors associated with COVID-19 disease severity in patients with cancer in Wuhan, China: a multicentre, retrospective, cohort study. *Lancet Oncology* 2020; **21**(7): 893-903.
9. Mato AR, Roeker LE, Lamanna N, et al. Outcomes of COVID-19 in patients with CLL: a multicenter international experience. *Blood* 2020; **136**(10): 1134-43.
10. Vuagnat P, Frelaut M, Ramtohul T, et al. COVID-19 in breast cancer patients: a cohort at the Institut Curie hospitals in the Paris area. *Breast Cancer Research* 2020; **22**(1): 55.
11. Fox TA, Troy-Barnes E, Kirkwood AA, et al. Clinical outcomes and risk factors for severe COVID-19 in patients with haematological disorders receiving chemo- or immunotherapy. *British Journal of Haematology* 2020; **191**(2): 194-206.
12. Sanchez-Pina JM, Rodríguez Rodriguez M, Castro Quismondo N, et al. Clinical course and risk factors for mortality from COVID-19 in patients with haematological malignancies. *European Journal of Haematology* 2020; **105**(5): 597-607.
13. Kalinsky K, Accordino MK, Hosi K, et al. Characteristics and outcomes of patients with breast cancer diagnosed with SARS-Cov-2 infection at an academic center in New York City. *Breast Cancer Res Treat* 2020; **182**(1): 239-42.
14. Malard F, Genthon A, Brissot E, et al. COVID-19 outcomes in patients with hematologic disease. *Bone Marrow Transplant* 2020; **55**(11): 2180-4.
15. Stroppa EM, Toscani I, Citterio C, et al. Coronavirus disease-2019 in cancer patients. A report of the first 25 cancer patients in a western country (Italy). *Future oncology (London, England)* 2020; **16**(20): 1425-32.
16. Bogani G, Ditto A, Bosio S, Brusadelli C, Raspagliesi F. Cancer patients affected by COVID-19: Experience from Milan, Lombardy. *Gynecologic Oncology* 2020; **158**(2): 262-5.
17. He WJ, Chen L, Chen L, et al. COVID-19 in persons with haematological cancers. *Leukemia* 2020; **34**(6): 1637-45.
18. Lattenist R, Yildiz H, De Greef J, Bailly S, Yombi JC. COVID-19 in Adult Patients with Hematological Disease: Analysis of Clinical Characteristics and Outcomes. *Indian journal of hematology & blood transfusion : an official journal of Indian Society of Hematology and Blood Transfusion* 2020: 1-5.
19. Yu J, Ouyang W, Chua MLK, Xie C. SARS-CoV-2 Transmission in Patients With Cancer at a Tertiary Care Hospital in Wuhan, China. *JAMA Oncology* 2020; **6**(7): 1108-10.
20. Robilotti EV, Babady NE, Mead PA, et al. Determinants of COVID-19 disease severity in patients with cancer. *Nature Medicine* 2020; **26**(8): 1218-23.
21. Angelis V, Tippu Z, Joshi K, et al. Defining the true impact of coronavirus disease 2019 in the at-risk population of patients with cancer. *Eur J Cancer* 2020; **136**: 99-106.

© 2022 Khoury E et al. *JAMA Network Open.*

22. Jee J, Foote MB, Lumish M, et al. Chemotherapy and COVID-19 Outcomes in Patients With Cancer. *Journal of Clinical Oncology* 2020; **38**(30): 3538-46.
23. Meng Y, Lu W, Guo E, et al. Cancer history is an independent risk factor for mortality in hospitalized COVID-19 patients: a propensity score-matched analysis. *J Hematol Oncol* 2020; **13**(1): 75.
24. Cook G, John Ashcroft A, Pratt G, et al. Real-world assessment of the clinical impact of symptomatic infection with severe acute respiratory syndrome coronavirus (COVID-19 disease) in patients with multiple myeloma receiving systemic anti-cancer therapy. *British Journal of Haematology* 2020; **190**(2): e83-e6.
25. Erdal GS, Polat O, Erdem GU, et al. The mortality rate of COVID-19 was high in cancer patients: a retrospective single-center study. *Int J Clin Oncol* 2021; **26**(5): 826-34.
26. Yarza R, Bover M, Paredes D, et al. SARS-CoV-2 infection in cancer patients undergoing active treatment: analysis of clinical features and predictive factors for severe respiratory failure and death. *European journal of cancer (Oxford, England : 1990)* 2020; **135**: 242-50.
27. Martín-Moro F, Marquet J, Piris M, et al. Survival study of hospitalised patients with concurrent COVID-19 and haematological malignancies. *British Journal of Haematology* 2020; **190**(1): e16-e20.
28. Guan W-j, Liang W-h, Zhao Y, et al. Comorbidity and its impact on 1590 patients with COVID-19 in China: a nationwide analysis. *European Respiratory Journal* 2020; **55**(5): 2000547.
29. Song K, Gong H, Xu B, et al. Association between recent oncologic treatment and mortality among patients with carcinoma who are hospitalized with COVID-19: A multicenter study. *Cancer* 2021; **127**(3): 437-48.
30. Basse C, Diakite S, Servois V, et al. Characteristics and Outcome of SARS-CoV-2 Infection in Cancer Patients. *JNCI Cancer Spectrum* 2021; **5**(1).
31. Joharatnam-Hogan N, Hochhauser D, Shiu K-K, et al. Outcomes of the 2019 novel coronavirus in patients with or without a history of cancer: a multi-centre North London experience. *Therapeutic Advances in Medical Oncology* 2020; **12**: 1758835920956803.
32. Shah V, Ko TK, Zuckerman M, et al. Poor outcome and prolonged persistence of SARS-CoV-2 RNA in COVID-19 patients with haematological malignancies; King's College Hospital experience. *British Journal of Haematology* 2020; **190**(5): E279-E82.
33. Hultcrantz M, Richter J, Rosenbaum C, et al. COVID-19 infections and outcomes in patients with multiple myeloma in New York City: a cohort study from five academic centers. *medRxiv : the preprint server for health sciences* 2020: 2020.06.09.20126516.
34. Grivas P, Khaki AR, Wise-Draper TM, et al. Association of clinical factors and recent anticancer therapy with COVID-19 severity among patients with cancer: a report from the COVID-19 and Cancer Consortium. *Ann Oncol* 2021; **32**(6): 787-800.
35. Sharafeldin N, Bates B, Song Q, et al. Outcomes of COVID-19 in Patients With Cancer: Report From the National COVID Cohort Collaborative (N3C). *Journal of Clinical Oncology*; **0**(0): JCO.21.01074.
36. Singh SRK, Thanikachalam K, Jabbour-Aida H, Poisson LM, Khan G. Covid-19 and cancer: Lessons learnt from a michigan hotspot. *Cancers* 2020; **12**(9): 1-13.
37. Sun L, Surya S, Le AN, et al. Rates of COVID-19-Related Outcomes in Cancer Compared With Noncancer Patients. *JNCI Cancer Spectr* 2021; **5**(1): pkaa120.
38. Brar G, Pinheiro LC, Shusterman M, et al. COVID-19 Severity and Outcomes in Patients With Cancer: A Matched Cohort Study. *Journal of Clinical Oncology* 2020; **38**(33): 3914-24.

39. Mehta V, Goel S, Kabarriti R, et al. Case Fatality Rate of Cancer Patients with COVID-19 in a New York Hospital System. *Cancer discovery* 2020; **10**(7): 935-41.
40. Ciceri F, Castagna A, Rovere-Querini P, et al. Early predictors of clinical outcomes of COVID-19 outbreak in Milan, Italy. *Clin Immunol* 2020; **217**: 108509.
41. Kabarriti R, Brodin NP, Maron MI, et al. Extent of Prior Lung Irradiation and Mortality in COVID-19 Patients With a Cancer History. *Advances in Radiation Oncology* 2020; **5**(4): 707-10.
42. Lunski MJ, Burton J, Tawagi K, et al. Multivariate mortality analyses in COVID-19: Comparing patients with cancer and patients without cancer in Louisiana. *Cancer*; **n/a**(n/a).
43. Russell B, Moss C, Papa S, et al. Factors Affecting COVID-19 Outcomes in Cancer Patients: A First Report From Guy's Cancer Center in London. *Frontiers in Oncology* 2020; **10**.
44. Dai M, Liu D, Liu M, et al. Patients with Cancer Appear More Vulnerable to SARS-CoV-2: A Multicenter Study during the COVID-19 Outbreak. *Cancer discovery* 2020; **10**(6): 783-91.
45. de Melo AC, Thuler LCS, da Silva JL, et al. Cancer inpatients with COVID-19: A report from the Brazilian National Cancer Institute. *PLoS One* 2020; **15**(10): e0241261.
46. Lièvre A, Turpin A, Ray-Coquard I, et al. Risk factors for Coronavirus Disease 2019 (COVID-19) severity and mortality among solid cancer patients and impact of the disease on anticancer treatment: A French nationwide cohort study (GCO-002 CACOVID-19). *European Journal of Cancer* 2020; **141**: 62-81.
47. de Joode K, Dumoulin DW, Tol J, et al. Dutch Oncology COVID-19 consortium: Outcome of COVID-19 in patients with cancer in a nationwide cohort study. *European Journal of Cancer* 2020; **141**: 171-84.

© 2022 Khoury E et al. *JAMA Network Open.*

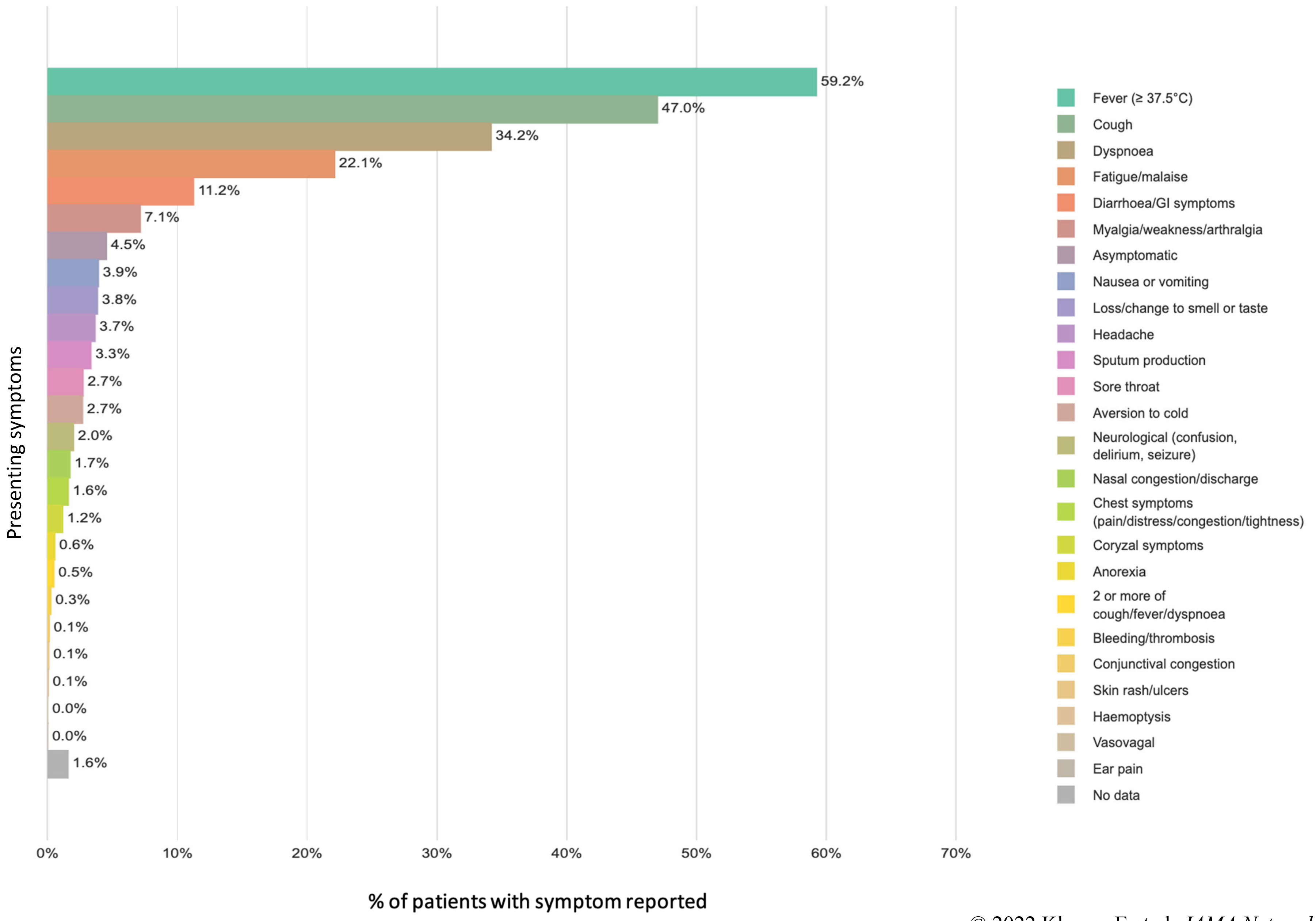

eFigure 13 Presenting symptoms, includes 9,196 patients.

© 2022 Khoury E et al. *JAMA Network Open*.

Publication	Imaging Modality	Bilateral involvement	Ground glass	Patchy shadows	Consolidation	Infiltrates	Other*
**Pinato et al**	CT or CXR: 811	427					
**Robilotti et al**	CT: 39, CXR: 207	23	30		13		32
**Jee et al**	-	76			34		
**Song et al**	-	188			53		185
**Tian et al**	CT: 232	150	148	126			165
**Yang K et al**	CT: 190	173	132				
**Mato et al**	CT or CXR: 183						
**Albiges et al**	CT: 133						
**Basse et al**	CT: 80		24		7		
**Angelis et al**	CT: 20, CXR: 86					47	
**Meng et al**	-	27	25				
**Cook et al**	-					56	
**Yarza et al**	-	33				60	
**Vuagnat et al**	CT: 39		14				
**Fox et al**	CT or CXR: 9		71				7
**Sanchez-Pina et al**	CT: 39	28					
**Martin-Moro et al**	-					30	
**Joharatnam-Hogan et al**	CXR: 29						
**Zhang L et al**	CT: 28		21		13		4
**Kalinsky et al**	CT: 39						
**Malard et al**	CT: 14, CXR: 7	21					
**Stroppa et al**	CT: 22, CXR: 3	22					25
**Bogani et al**	CT: 18						
**Guan et al**	CT: 14, CXR: 7						
**He et al**	CT: 22, CXR: 3		8	2	1		1
**Lattenist et al**	CT: 18						
**Yu et al**	CT: 11						
**Total:**	CT: 958, CXR: 525, either: 1,003	1,168	473	128	121	193	419

eTable 8. Chest radiograph ± chest x ray imaging on admission with radiological changes documented, where reported**.** *Nodules/interstitial thickening/erratic paving.

© 2022 Khoury E et al. *JAMA Network Open.*

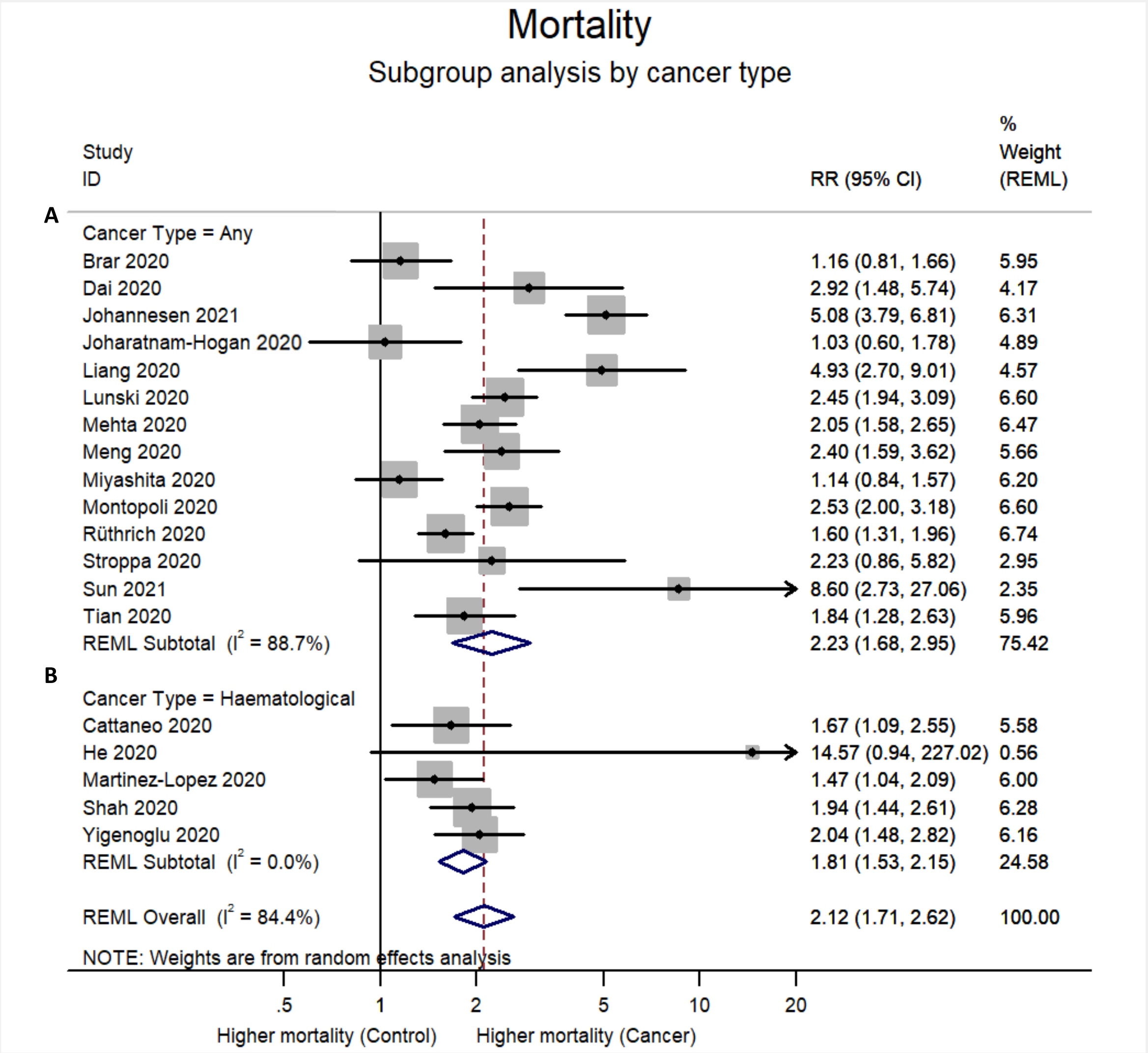

eFigure 14. (A) Overall risk of mortality in COVID-19 patients with any cancer type compared with those with haematological malignancies. (B) Overall risk of mortality in COVID-19 patients with haematological malignancies as compared to those with solid malignancies.

© 2022 Khoury E et al. *JAMA Network Open*.

A) Age (obs: 14)					tau2=0.06352, I^2=54.7%
Effect Size	*exp (b)*	*Standard Error*	*t*	*P >|t|*	*[95% Confidence Interval]*
Age	0.9572513	0.0166774	-2.51	0.028	0.9215953 – 0.9942868
**B) Male cancer patients (obs: 17)**					**tau2=0.08595, I^2=67.92%**
Effect Size	*exp (b)*	*Standard Error*	*t*	*P >|t|*	*[95% Confidence Interval]*
Male cancer patients	1.187946	0.935615	0.22	0.830	0.221688 – 6.365771
**C) Age and male cancer patients (obs: 14)**					**tau2=0.06999, I^2=57.07%**
Effect Size	*exp (b)*	*Standard Error*	*t*	*P >|t|*	*[95% Confidence Interval]*
Age	0.9517435	0.0188232	-2.50	0.029	0.9112026 – 0.994088
Male cancer patients	3.526408	7.123145	0.62	0.545	0.0413537 – 300.7112

eTable 9 Meta-regression results on the impact of (A) age, (B) male sex, and (C) both age and male sex.
Obs, observations.

© 2022 Khoury E et al. *JAMA Network Open.*

	Cancer covid patients	Not hospitalised/ unknown	Number hospitalised	ICU admission	IMV	CRRT	ECMO	Haemo-dialysis	IV Vasopre-ssors	Transition to DNR/DNI	Discharged	Remain Hospitalised	Death
**Sharafeldin et al, 2021**	**38,614**	19,099	19,515		1,600		27						2,888
**Grivas et al, 2020**	**4,966**	2,094	2,872	232	292								695
**Özdemir et al, 2020**	**1,523**	661	862	173	113								77
**Lievre et al, 2020**	**1289**	555	734	110	49								322
**Lee et al, 2020**	**1044**	1003	41	63	43							41	295
**Pinato et al, 2020**	**890**	120	760	110	35								299
**Yigenoglu et al, 2020**	**740**	452	288	140	102								102
**Johannesen et al, 2020**	**547**	427	120	17	17								56
**Passamonti et al, 2020**	**536**	85	451	82							242	11	198
**Rüthrich et al, 2020**	**435**	8	427	119	78						292	46	97
**Robilotti et al, 2020**	**423**	243	180	48	40								51
**Miyahsita et al, 2020**	**334**				37								37
**Lunski et al, 2020**	**312**	146	166	48									66
**Jee et al, 2020**	**309**	162	147	43									31
**Tian et al, 2020**	**232**	0	232		21								46
**Di Cosimo et al, 2020**	**231**	66	165	12	19								81
**Mehta V et al, 2020**	**218**	28	190	23	45							35	61
**Yang K et al, 2020**	**205**	0	205	30	21	5		5			165		40

© 2022 Khoury E et al. *JAMA Network Open.*

	Cancer covid patients	Not hospitalised/ unknown	Number hospitalised	ICU admission	IMV	CRRT	ECMO	Haemo-dialysis	IV Vasopre-ssors	Transition to DNR/DNI	Discharged	Remain Hospitalised	Death
**Ferrari et al, 2020**	**198**	110	88	37							149	16	33
**Garassino et al, 2020**	**198**	48	152	13	9							92	66
**Mato et al, 2020**	**198**	20	178	68	53				47		63	49	66
**Scarfo et al, 2020**	**190**	21	169	39							96	37	56
**Mehta A et al, 2020**	**186**				12								27
**de Melo et al, 2020**	**178**	53	125	80									60
**Albiges et al, 2020**	**167**	0	167		15		16				80	16	31
**Martinez-Lopez et al, 2020**	**167**	0	167		15						110	1	56
**Russell et al, 2020**	**156**	38	118	13									34
**Basse et al, 2020**	**141**	91	50	11							100	11	26
**Lara et al, 2020**	**121**	55	66	20	9						39	11	18
**Montopoli et al, 2020**	**118**	8	78	14									18
**Brar et al, 2020**	**117**	117	0		4						85	3	29
**Angelis et al, 2020**	**113**	12	101	12	8								29
**Meng et al, 2020**	**109**	0	109		9						77		32
**Zhang H et al, 2020**	**107**	0	107		18						84		23
**Dai et al, 2020**	**105**	0	105	20	11	4	3	4					12
**Luo et al, 2020**	**102**	39	63	21	18					18	53	24	25
**Cattaneo et al, 2020**	**102**			21	5								40

© 2022 Khoury E et al. *JAMA Network Open.*

	Cancer covid patients	Not hospitalised/ unknown	Number hospitalised	ICU admission	IMV	CRRT	ECMO	Haemo-dialysis	IV Vasopre-ssors	Transition to DNR/DNI	Discharged	Remain Hospitalised	Death
**Hultcrantz et al, 2020**	**100**	25	75	17	13								22
**Singh et al, 2020**	**85**	12	73	30	23	7	30		19	39	38	3	32
**Shah et al, 2020**	**80**	0	80										
**Cook et al, 2020**	**75**	3	72	9	6								41
**Erdal et al, 2020**	**71**	0	71	18	17								
**Sun et al, 2020**	**67**	30	37	17									9
**Booth et al, 2020**	**66**	0	66		3						28	4	34
**Yarza et al, 2020**	**63**	0	63	0	0								16
**Vuagnat et al, 2020**	**59**	31	28	4	1						45	10	4
**Wang B et al, 2020**	**58**	22	36	7	5						22	1	14
**Fox et al, 2020**	**55**	4	51		25	1			5		35	1	19
**Yang F et al, 2020**	**52**	0	52		0	1					41		11
**Suleyman et al, 2020**	**49**	6	43	23									19
**Rogado et al, 2020**	**45**	7	38									26	19
**Sanchez-Pina et al, 2020**	**39**	5	34	1									14
**Aries et al, 2020**	**35**										21	1	14
**Martin-Moro et al, 2020**	**34**	0	34	2	4							5	11

© 2022 Khoury E et al. *JAMA Network Open.*

	Cancer covid patients	Not hospitalised/ unknown	Number hospitalised	ICU admission	IMV	CRRT	ECMO	Haemo-dialysis	IV Vasopre-ssors	Transition to DNR/DNI	Discharged	Remain Hospitalised	Death
**Joharatnam-Hogan et al, 2020**	**30**	0	30										11
**Zhang L et al, 2020**	**28**	0	28	6	12						10		8
**Kalinsky et al, 2020**	**27**	20	7								26	0	1
**Malard et al, 2020**	**25**	0	25		6								9
**Stroppa et al, 2020**	**25**	0	25	1									9
**Ciceri et al, 2020**	**22**	0	22								11	0	11
**Bogani et al, 2020**	**19**	0	19	2							14	2	3
**Liang et al, 2020**	**18**	0	18	7									7
**Guan et al, 2020**	**18**	0	18	5	2								3
**Tagliamento et al, 2020**	**17**	4	13									13	4
**He et al, 2020**	**13**	0	13		1							0	8
**Lattenist et al, 2020**	**13**	0	13	2	1							0	6
**Yu et al, 2020**	**12**	0	12								9	3	3
**Wu et al, 2020**	**11**	4	7									1	4

eTable 10 Patient outcomes in 68 studies.
CRRT, continuous renal-replacement therapy; DNR/DNI, do not resuscitate/do not intubate; ECMO, extracorporeal membrane oxygenation; ICU, intensive care unit; IMV, invasive mechanical ventilation.

© 2022 Khoury E et al. *JAMA Network Open.*

Publication	Duration of hospital stay (days)
Pinato et al	10 (IQR 5-18)
Passamonti et al	16 (range 1-98)
Lunski et al	6 (IQR 3-15)
Song et al	22
Yang K et al	19 (IQR 12-33)
Garassino et al	12
Albiges et al	10 (range 1-40)
Lara et al	7 (IQR 4-10)
Dai et al*	27.01 (SD 9.52)
Singh et al	9.5 (range1-27)
Erdal et al	10 (range 1-39)
Yarza et al*	9.2 (95% CI 8.17-10.33)
Wang B et al	22
Fox et al	13 (range 0-135)
Zhang L et al	19 (IQR 16-28.5)

eTable 11. Median duration of hospital stay (days).
*Mean duration.

© 2022 Khoury E et al. *JAMA Network Open.*

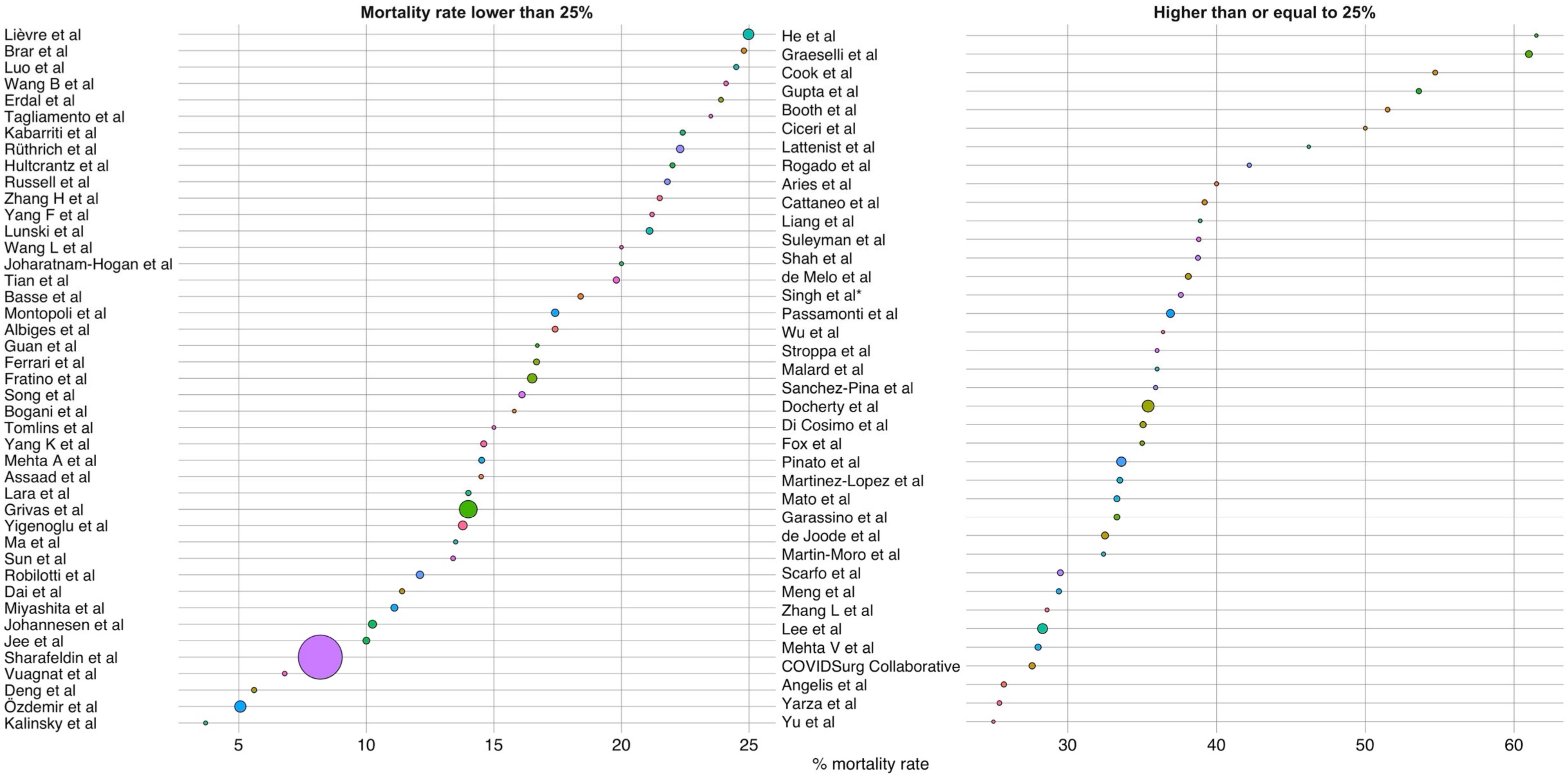

eFigure 15 Mortality reported across 81 studies.

© 2022 Khoury E et al. *JAMA Network Open.*

Publication	Mortality in those with cancer and COVID-19	Cancer COVID patients	%	Median follow up, days (range)	
				Median	Mean
Sharafeldin et al	3,164	38,614	8.19%	-	
Grivas et al	695	4966	14.0%	42 (22-90)	
Docherty et al	617	1743	35.4%	-	
Özdemir et al	77	1523	5.06%	50 (1-74)	
Lièvre et al	322	1289	24.98%	34 (32-36)	
Lee et al	295	1044	28.3%	6 (2-11)	
Fratino et al	150	909	16.5%	-	
Pinato et al	299	890	33.6%		19 (+/- 16.3)
Yigenoglu et al	102	740	13.78%		
Johannesen et al	56	547	10.24%		
Passamonti et al	198	536	36.9%	20 (10-34)	
Rüthrich et al	97	435	22.30%		
Montopoli et al	75	430	17.4%	-	
Robilotti et al	51	423	12.1%	-	
de Joode et al	114	351	32.5%	-	
Miyashita et al	37	334	11.1%	-	
Graeselli et al	202	331	61.0%	69 (60-78)	
Lunski et al	66	312	21.1%	-	
Jee et al	31	309	10.0%	-	
Song et al	40	248	16.1%	-	
COVIDSurg Collaborative	66	239	27.6%	-	
Tian et al	46	232	19.8%	29 (22-38)	
Di Cosimo et al	81	231	35.06%	138 (12-218)	
Mehta V et al	61	218	28.0%	-	
Yang K et al	30	205	14.6%	68 (59-78)	
Ferrari et al	33	198	16.67%	61	
Garassino et al	66	198	33.3%	15 (8-24)	
Mato et al	66	198	33.3%	16 (1-43)	
Scarfo et al	56	190	29.5%	23 (2-86)	
Mehta A et al	27	186	14.52%	63	
de Melo et al	69	181	38.1%	5 (2-10.3)	
Albiges et al	31	178	17.4%	23 (13-33)	
Martinez-Lopez et al	56	167	33.5%	-	
Russell et al	34	156	21.8%	37 (18-49)	
Basse et al	26	141	18.4%	-	
Lara et al	17	121	14.0%	-	
Brar et al	29	117	24.8%		
Angelis et al	29	113	25.7%	-	
Gupta et al	60	112	53.6%	16 (8-28)	
Meng et al	32	109	29.4%	-	
Deng et al	6	107	5.6%	-	
Kabarriti et al	24	107	22.4%	7 (0.5-39)	
Zhang H et al	23	107	21.5%	-	
Dai et al	12	105	11.4%	-	
Luo et al	25	102	24.5%	25 (10-36)	
Cattaneo et al	40	102	39.2%	-	
Hultcrantz et al	22	100	22.0%	-	
Singh et al*	32	85	37.6%	31 (20-36)	
Shah et al	31	80	38.8%	-	
Cook et al	41	75	54.7%	-	
Erdal et al	17	71	23.9%	-	
Sun et al	9	67	13.4%	-	
Booth et al	34	66	51.5%	32.5	
Yarza et al	16	63	25.4%	-	

© 2022 Khoury E et al. *JAMA Network Open.*

Publication	Mortality in those with cancer and COVID-19	Cancer COVID patients	%	Median follow (range)<br>Median	up, days<br><br>Mean
Vuagnat et al	4	59	6.8%	-	
Wang B et al	14	58	24.1%	-	
Assaad et al	8	55	14.5%	25	
Fox et al	19	55	35.0%	27 (17-43)	
Yang F et al	11	52	21.2%	-	
Suleyman et al	19	49	38.8%	-	
Rogado et al	19	45	42.2%	14 (1-28)	
Sanchez-Pina et al	14	39	35.9%	-	
Ma et al	5	37	13.5%	-	
Aries et al	14	35	40.0%	-	
Martin-Moro et al	11	34	32.4%	26	
Joharatnam-Hogan et al	6	30	20.0%	-	
Zhang L et al	8	28	28.6%	-	
Kalinsky et al	1	27	3.7%	26 (1-38)	
Malard et al	9	25	36.0%	29 (14-40)	
Stroppa et al	9	25	36.0%	-	
Ciceri et al	11	22	50.0%	14 (7-25)	
Tomlins et al	3	20	15.0%	-	
Bogani et al	3	19	15.8%	-	
Liang et al	7	18	38.9%	-	
Guan et al	3	18	16.7%	10 (8-14)	
Tagliamento et al	4	17	23.5%	15	
Wang L et al	3	15	20.0%	-	
He et al	8	13	61.5%	-	
Lattenist et al	6	13	46.2%	-	
Yu et al	3	12	25.0%	-	
Wu et al	4	11	36.4%	-	

eTable 12 Mortality of cancer patients reported across studies.
* Death defined mortality + transfer to hospice, not reported separately.

© 2022 Khoury E et al. *JAMA Network Open.*

	Admission to hospital	Supplemental O2	Bilateral pneumonia with CURB-65 score ≥ 2/FiO2 ≥ 35%	Admission to ICU	ICU/intubate/ do not intubate	Mechanical ventilation	Invasive ventilation	ARDS	Septic shock	≥ 1 complication from COVID-19 [1]	Death	WHO Severity Scale [2]	National Health Commission of China definition [3]	Ministry of Health and Family Welfare definition [4]
Grivas et al, 2020	■			■		■					■			
Docherty et al, 2020				■							■			
Lee et al, 2020												■		
Pinato et al, 2020										■				
Yigenoglu et al, 2020													■	
Passamonti et al, 2020													■	
Montopoli et al, 2020	■			■							■			
Robilotti et al, 2020		■				■								
Jee et al, 2020						■					■		■	
Tian et al, 2020												■	■	
Mehta V et al, 2020				■		■								
Yang K et al, 2020				■		■					■			
Scarfo et al, 2020	■	■		■										
Mehta A et al, 2020														■
Russell et al, 2020								■	■					
Basse et al, 2020				■							■			
Zhang H et al, 2020														
Dai et al, 2020				■		■					■		■	
Luo et al, 2020	■				■						■			
Hulcrantz et al, 2020				■		■					■			
Yarza et al, 2020								■			■			
Vuagnat et al, 2020				■							■			
Yang F et al, 2020													■	
Rogado et al, 2020			■	■										
Sanchez-Pina et al, 2020												■		
Ma et al, 2020													■	
Joharatnam-Hogan et al, 2020				■		■					■			
Zhang L et al, 2020				■		■					■			
Malard et al, 2020				■		■					■			
Stroppa et al, 2020													■	
Liang et al, 2020				■			■				■			
Guan et al, 2020				■		■					■			
Tagliamento et al, 2020				■		■					■			
Wang L et al, 2020													■	
He et al, 2020													■	
Wu et al, 2020													■	

eFigure 16 Definition of severe event.
[1]Complications include acute cardiac injury, ARDS, and septic shock. [2]Clinical signs of pneumonia (fever, cough, dyspnoea, fast breathing) plus one of the following: respiratory rate > 30 breaths/min, severe respiratory distress; or SpO2 < 90% on room air. [3]Reparatory distress (≥ 30 breaths/min), oxygen saturation ≤ 93% at rest, arterial partial pressure of oxygen (PaO2)/fraction of inspired oxygen (FiO2) ≤ 300 mmHg. ARDS: Acute Respiratory Distress Syndrome; ICU, intensive care unit.

© 2022 Khoury E et al. *JAMA Network Open.*

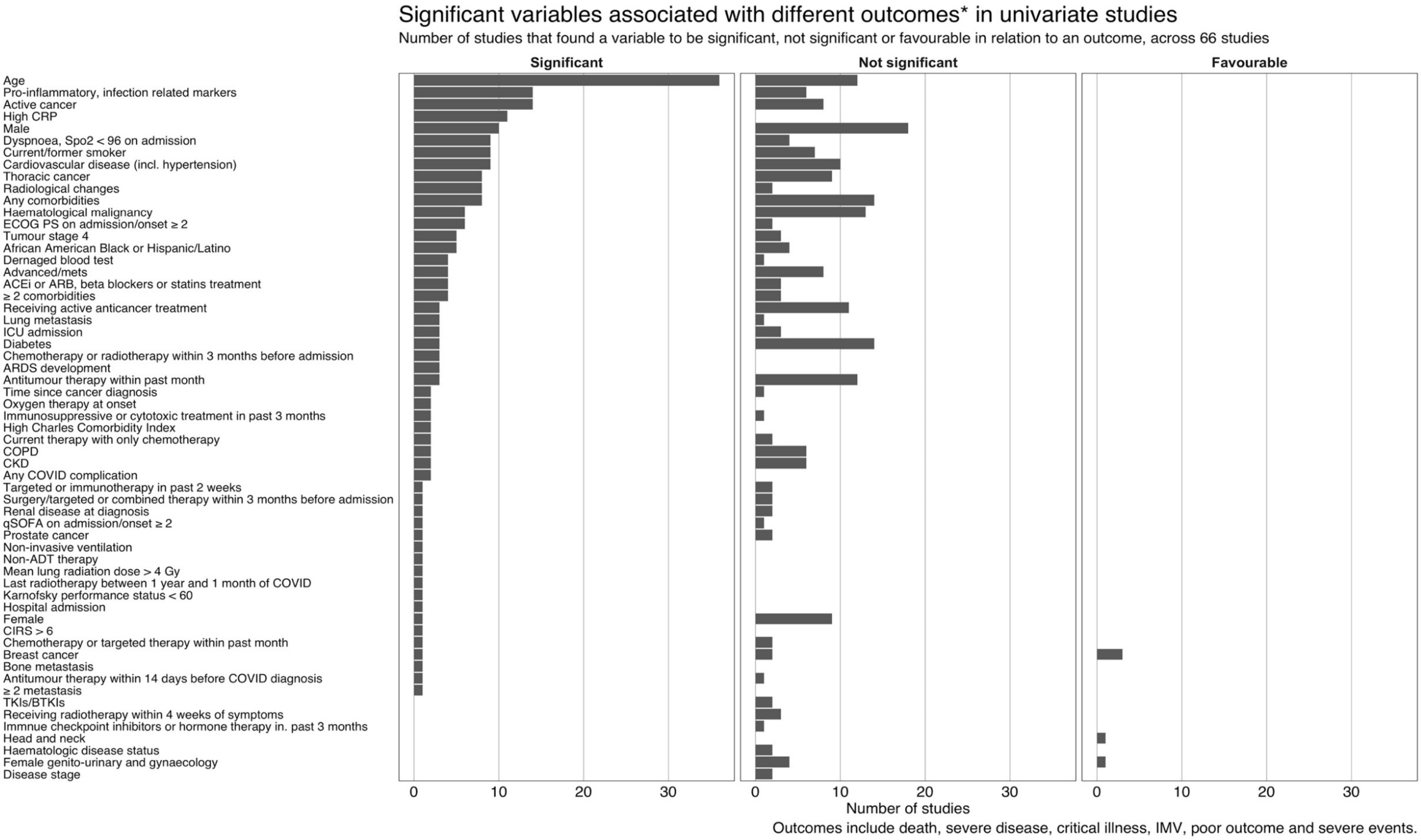

eFigure 17. Significant Variables in Unadjusted (A) and Adjusted (B) Analyses Across Studies
ACEi, angiotensin converting enzyme inhibitor; ARB, angiotensin II receptor blocker; ARDS, acute respiratory distress syndrome; CIRS, cumulative illness rating score; CT, computerised tomography; ECOG, Eastern Cooperative Oncology Group; ICU, intensive care unit; PS, performance status; VTED, venous thromboembolic disease.

© 2022 Khoury E et al. *JAMA Network Open.*

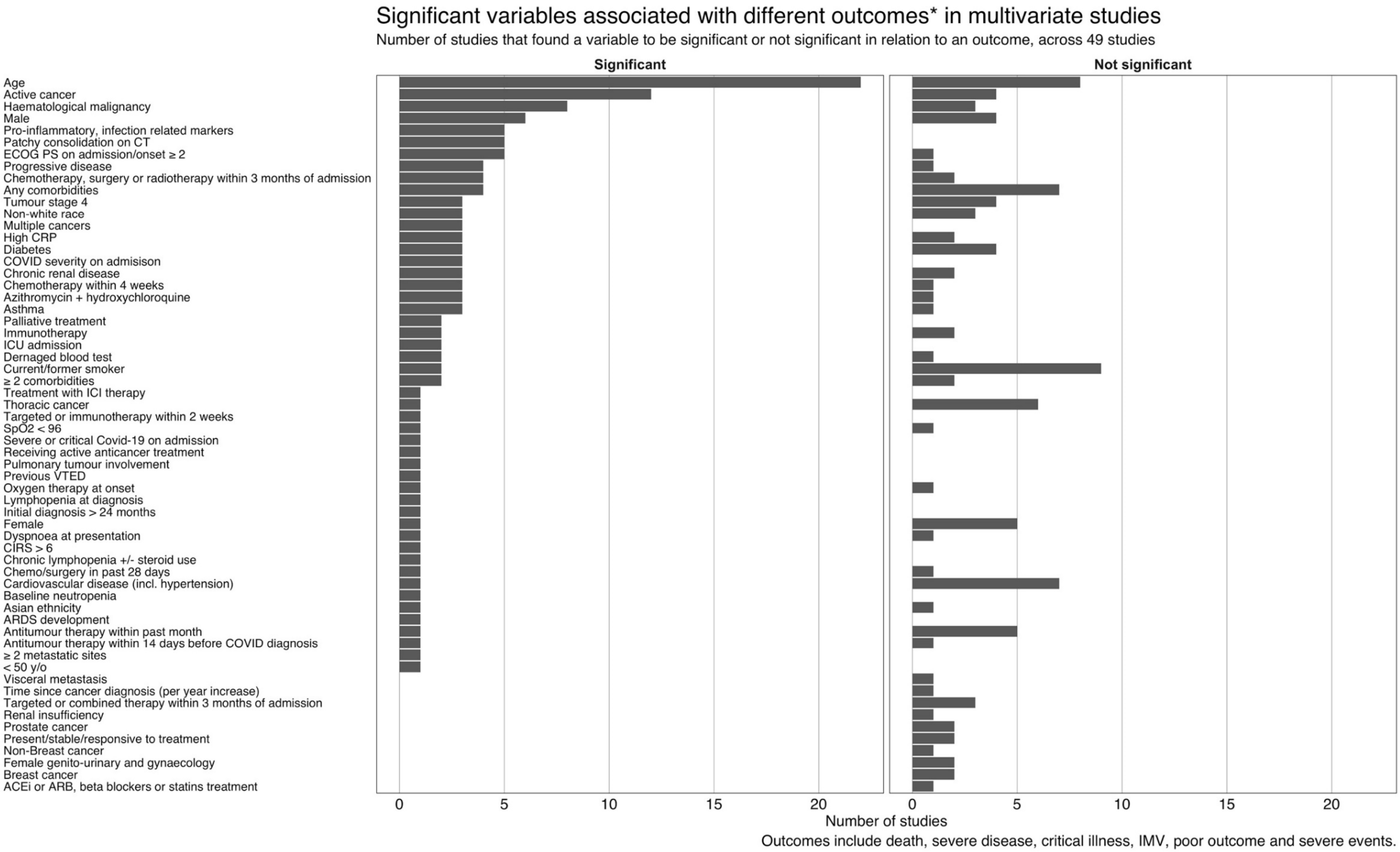

eFigure 17. Continued
ACEi, angiotensin converting enzyme inhibitor; ARB, angiotensin II receptor blocker; ARDS, acute respiratory distress syndrome; CIRS, cumulative illness rating score; CT, computerised tomography; ECOG, Eastern Cooperative Oncology Group; ICU, intensive care unit; PS, performance status; VTED, venous thromboembolic disease

© 2022 Khoury E et al. *JAMA Network Open*.

	Age	Gender	Race	BMI	Deprivation Index	Smoking status (former vs never smoked)	Co-morbidities	Chronic lymphopenia or corticosteroids	Medications *	ECOG performance	Cancer type	Tumour stage	Tumour status	Time since cancer diagnosis	Line of treatment	Therapeutic intent	Antitumour treatments	Time from malignancy to COVID-19 diagnosis	Radiological changes associated with COVID-19	Pro-inflammatory blood markers	Pulmonary involvement	Pre-admission characteristics	COVID-19 severity (including ARDS development)	Treatment with azithromycin or hydroxychloroquine	Immuno-therapy or surgery	Active chemo-therapy/ immuno-suppresive therapy
Sharafeldin et al	■	■	■			■	■				■						■									
Docherty et al	■	■					■																			
Lee et al	■	■																								
Grivas et al	■	■	■			■	■			■	■		■				■									
Pinato et al	■	■					■																			
Passamonti et al	■	■					■				■		■				■	■					■			
Robilotti et al	■		■			■	■	■			■						■									
Miyashita et al	■																									
Graeselli et al	■	■					■		■														■			
Lunski et al	■	■	■				■																			
Jee et al	■	■		■		■	■			■	■							■		■						
Song et al -																										
COVIDSurg Collaborative																			■			■				
Tian et al	■	■					■				■	■					■									
Mehta et al	■	■					■													■			■			
Yang K et al		■									■			■			■									
Garassino et al	■	■				■	■																			
Mato et al	■						■																			
Martinez-Lopez et al	■	■					■					■										■				
Russell et al	■	■	■		■	■	■			■	■			■			■			■			■			
Basse et al	■	■					■			■	■	■			■	■			■				■			
Lara et al	■		■			■	■			■															■	
Angelis et al	■														■					■						
Gupta et al	■	■	■	■		■	■						■							■						
Zhang H et al	■																■									
Dai et al	■	■				■	■																			
Cattaneo	■	■																					■			■
Singh et al	■	■	■		■		■																			
Yarza et al	■	■				■	■			■	■	■							■		■					
Suleyman et al	■	■	■				■												■	■			■			
Rogado et al	■						■				■	■					■									
Sanchez-Pina et al	■						■		■		■												■			
Martin-Moro et al	■									■	■												■	■		
Zhang L et al	■	■																								
Ciceri et al	■	■					■												■	■						
Liang et al	■					■	■																			
Guan et al	■					■																				
Wang L et al	■						■																■			

eFigure 18 Variables included in adjusted analysis across studies.

© 2022 Khoury E et al. *JAMA Network Open.*

A

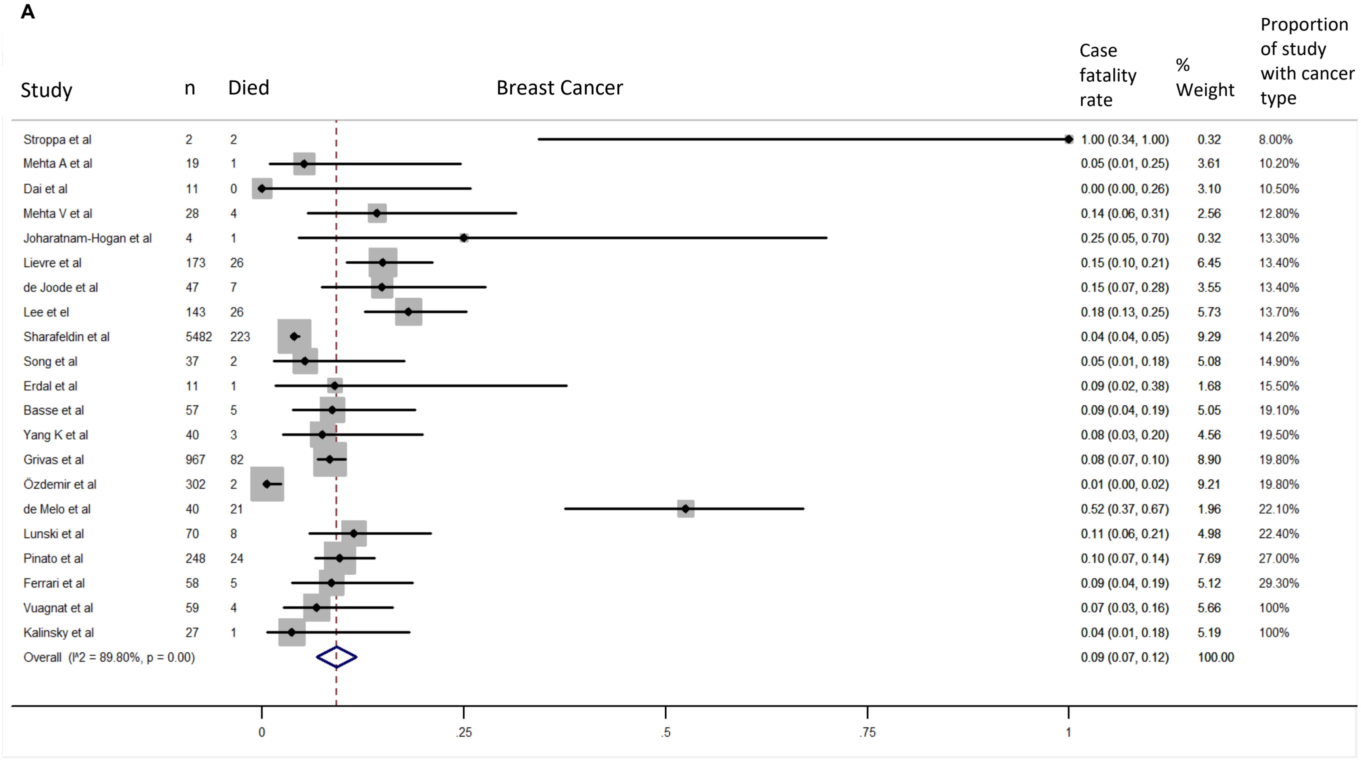

B

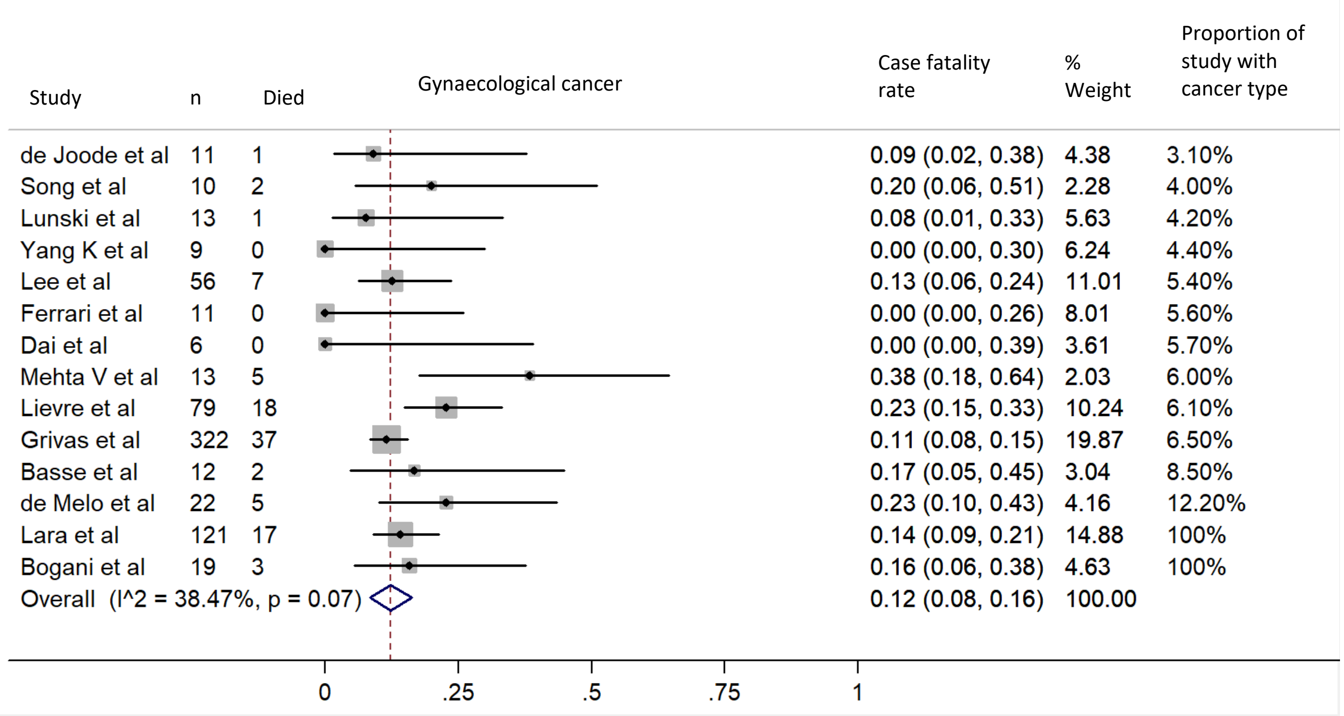

© 2022 Khoury E et al. *JAMA Network Open.*

C

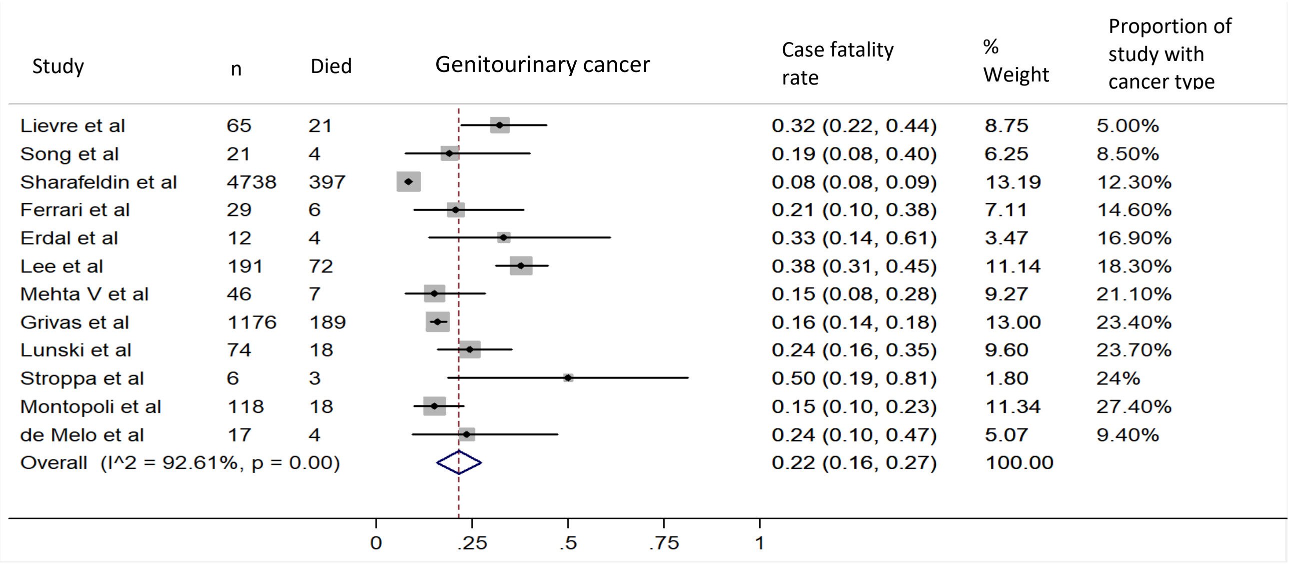

D

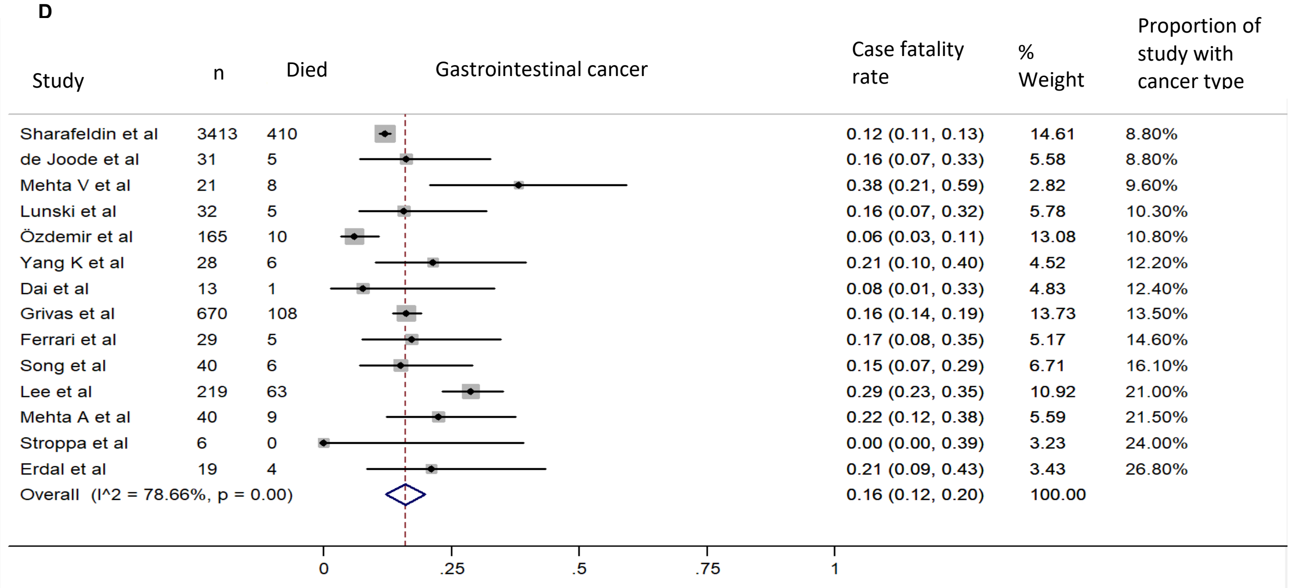

E

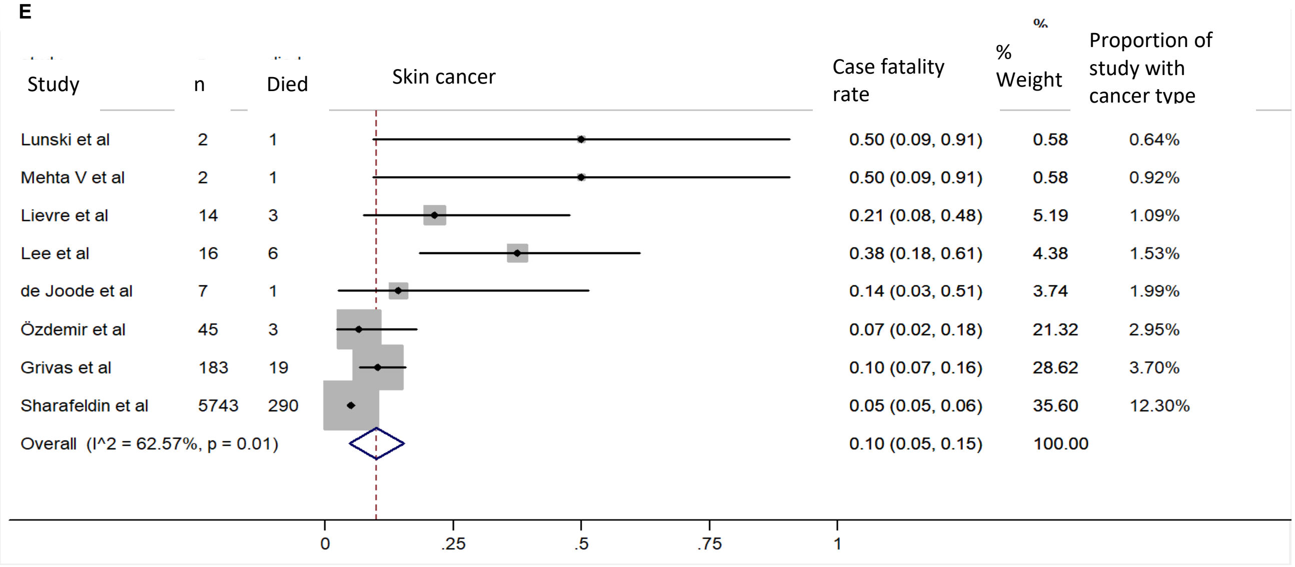

© 2022 Khoury E et al. *JAMA Network Open.*

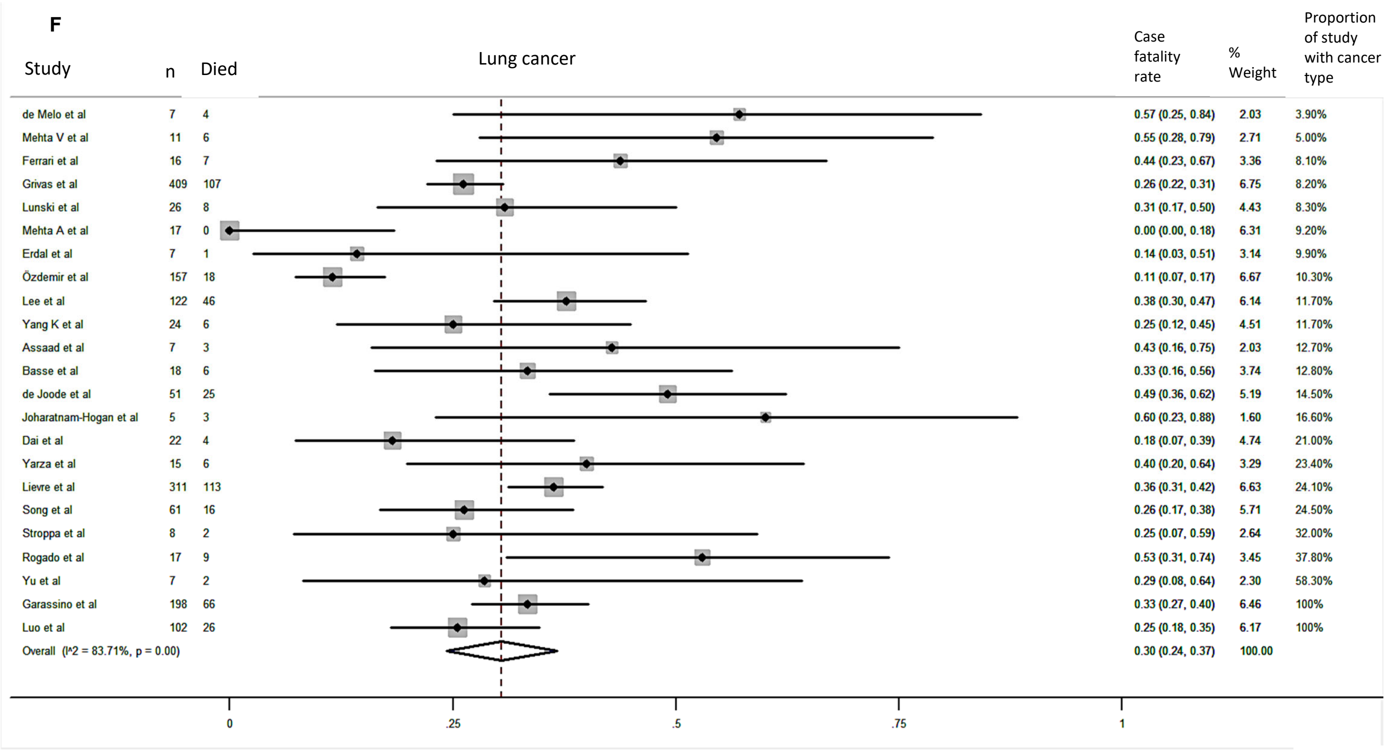

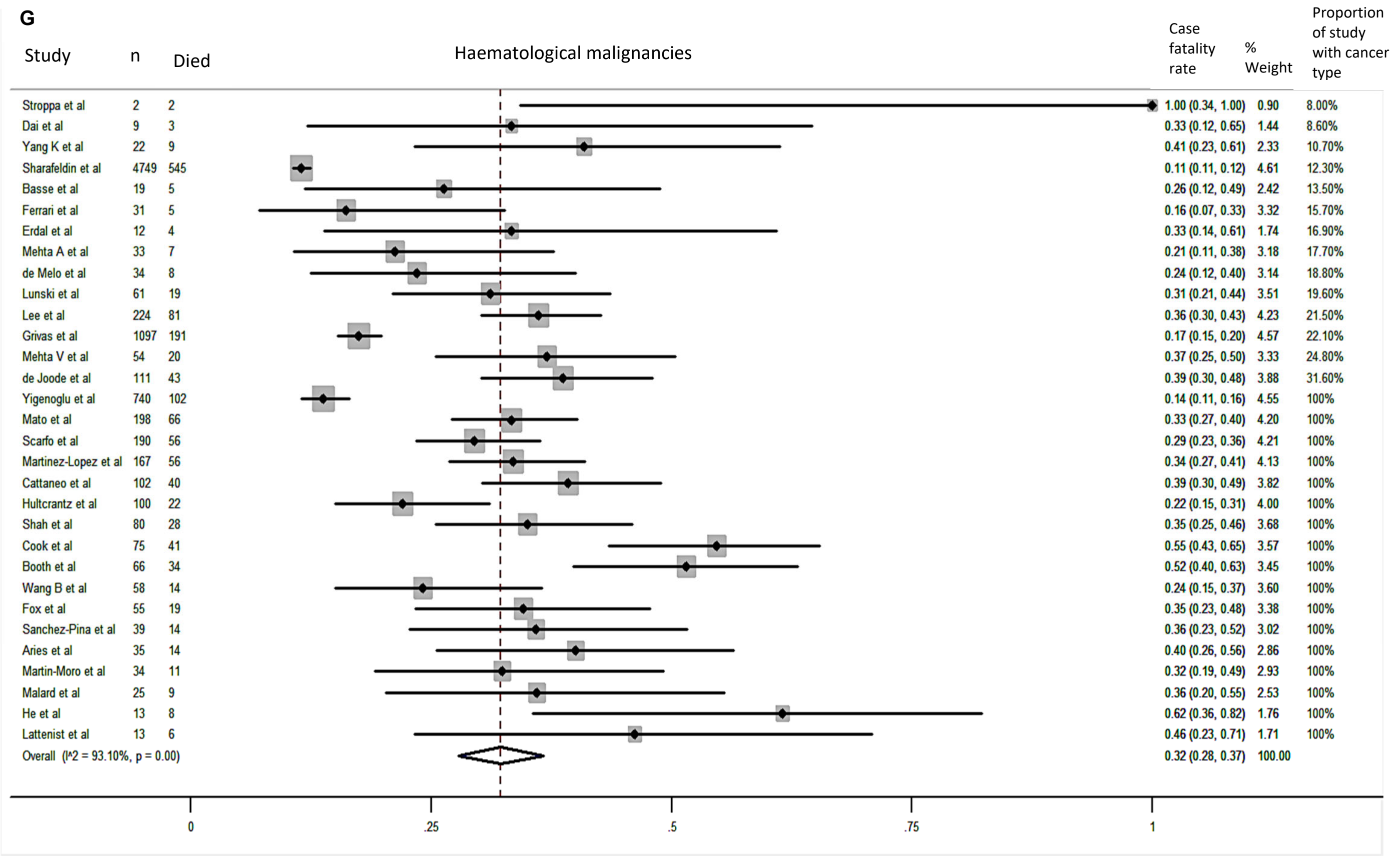

eFigure 19 Forest plot of overall pooled case fatality in subgroup analysis
Case fatality in different cancer types: A) Breast cancer, B) Gynaecological cancer, C) Genitourinary cancer, D) Gastrointestinal cancer, E) Skin cancer, F) Lung cancer, and G) Haematological malignancies.

© 2022 Khoury E et al. *JAMA Network Open.*

Type of cancer treatment	Number of studies included in analysis	Number of patients included across studies (range)	Pooled case fatality rate
Surgery	7	4 to 56	19%
Chemotherapy	22	3 to 802	31%
Endocrine	9	4 to 483	11%
Immunotherapy	14	3 to 248	22%
Radiotherapy	9	2 to 95	20%
Targeted therapy	15	1 to 693	18%

eTable 13 Pooled case fatality rates for various cancer treatments.

© 2022 Khoury E et al. *JAMA Network Open.*

Name of Study	Trial or Registration Number	Location	Number of patients and patient characteristics	Primary endpoint
CovidSurg – Cancer	NCT04384926	Global	1000 patients planned for cancer surgery	30-day postoperative COVID-19 infection rate. https://globalsurg.org/cancercovidsurg/
COVID-19 and Cancer Consortium Registry (CCC19)	NCT04354701	Global	10,000 patients	Collect data about cancer patients who have been infected with COVID-19 via web-based REDCap Survey. https://ccc19.org/
American Society of Haematology Research Collaborative (ASH RC) COVID-19 Registry for Hematologic Malignancy	-	Global	-	Captures data on patients with COVID-19 and have been or are currently being treated for hematologic malignancy. https://www.ashresearchcollaborative.org/covid-19-registry
ASCO Survey on COVID-19 in Oncology (ASCO) Registry	NCT04659135	USA	2000 patients	Changes to Cancer Treatments.
Thoracic cancers international COVID-19 collaboration (TERAVOLT)	-	Global	-	A global consortium designed to gather information on patients with thoracic cancer infected with COVID-19 regardless of therapies administered. http://www.etop-eu.org/index.php?option=com_content&view=article&id=115644&catid=13&Itemid=557
Clinical Characterisation Protocol-Cancer-UK	NCT04603105	UK	9000 patients	To determine the COVID-19 fatality rate overall in the cancer population using the most up to date dataset from the first wave as well as to determine the COVID-19 fatality rate in different tumour types. https://isaric.tghn.org/UK-CCP.
UK Coronavirus Cancer Monitoring Project	-	UK	-	The UK Coronavirus cancer monitoring scheme is a clinician-led reporting project recoding data related to cancer patients who have tested positive for COVID-19 across the UK. https://ukcoronaviruscancermonitoring.com/

© 2022 Khoury E et al. *JAMA Network Open.*

Name of Study	Trial or Registration Number	Location	Number of patients and patient characteristics	Primary endpoint
ONCOVID:	NCT04393974	UK	1000 patients	Describe presenting characteristics and severity or SARS-CoV-2 infection in patients with cancer. To assess what factors are involved in prognosis of cancer patients with COVID-19. https://www.oncovid.net/
Prospective Analysis of Morbi-mortality of Patients With Cancers in Active Phase of Treatment Suspected or Diagnosed of a SARS-CoV-2 Infection (ONCOVID-19)	NCT04363632	France	1231 patients	Mortality of cancer patients under active anticancer treatment.
UK Covid and gynaecological cancer study (UKCOGS-UK)	-	UK	-	Evaluate the MDT decision making for gynaecological cancer, patient outcomes across the UK in response to the COVID-19 pandemic. bb-ukcogs@qmul.ac.uk
PACE: Patients with AML and COVID-19 Epidemiology	282870 (IRAS ID)	UK	100 patients with AML	Record how many patients have had COVID-19 previously, have an active infection or go on to develop COVID-19 whilst receiving treatment for their AML.
Covid-19 in Hematological Malignancies (EPICOVIDEHA)	NCT04733729	Italy	3000 patients with haematological malignancies	Epidemiology of COVID-19 infection in patients with haematological malignancies
Screening and Identification of SARS-CoV-2 Infection and Progression in Cancer Patients Based on Bioinformatics Analysis	ChiCTR2000030807	China	100 patients.	Clinical characteristics and prognosis of cancer patients with novel coronavirus pneumonia (COVID-19) based on bioinformatics analysis. http://www.chictr.org.cn/showproj.aspx?proj=51019
COVID-19 Infection and Multiple Myeloma (EMN-COVID)	NCT04492371	Global	500 patients with multiple myeloma and COVID-19 infection	Nature of COVID19, costs related to COVID-19, systemic anti-cancer therapy subgroup, laboratory values collected at hospitalization, COVID-19 infection in myeloma patient subgroups, incidence of COVID-19 infection in frail patients and infection outcome in different countries.

© 2022 Khoury E et al. *JAMA Network Open.*

Name of Study	Trial or Registration Number	Location	Number of patients and patient characteristics	Primary endpoint
LunG and Melanoma canceR pAtients coVId19 Disease (GRAVID)	NCT04344002	Spain	200 patients with lung cancer and COVID-19	Clinical data of lung cancer patients with COVID-19 diagnoses, diagnosis data, treatments received and prognostic factors.
COVID-19 Infection in Cancer Patients (COICA)	NCT04569292	Italy	150 patients	Describe cancer patients with COVID-19 and their clinical course
Registry on NEN Patients and COVID-19	NCT04444401	Italy	50 patients with neuroendocrine tumours	Correlation between clinical parameters and SARS-CoV-2 infection as well as clinical outcome of SARS-CoV-2 Infection
Effects of COVID-19 Pandemic on the Diagnosis and Outcomes of Colorectal Cancer (COVID-CRC) (COVID-CRC)	NCT04712292	Italy	2000 patients	Oncologic stage according to TNM classification.
NCI COVID-19 in Cancer Patients, NCCAPS Study	NCT04387656	Global	2000 patients	Patient variables (factors) associated with severe acute respiratory syndrome (SARS) coronavirus 2 (COVID-19) severity, effects of COVID-19 on cancer therapy and association with clinical outcomes and physical health (patient-reported health-related quality of life).
Prospective Determination of COVID-19 Infection Rate in a Chemotherapy Unit in Mexico	NCT04567979	Mexico	149 patients with solid malignant disease and healthcare workers	SARS-CoV-2 infection rate in patients with solid tumours.
A Study of Risk Factors for the COVID-19 Virus Infection	NCT04697927	USA	10,000 patients	To develop a comprehensive registry database.
Registry of Patients With Hematologic Disease and COVID-19 in Russia (CHRONOS19)	NCT04422470	Russia	200 patients with malignant and non-malignant haematological disease	30-day all-cause mortality
Long-term Evolution of Pulmonary Involvement of Novel SARS-COV-2 Infection (COVID-19): Follow the Covid Study	NCT04605757	Italy	100 patients	Long term evolution of clinical involvement due to SARS-COV-2 pneumonia / symptoms, respiratory rate, blood gas exchange parameters, pulmonary function tests, ogical involvement.

© 2022 Khoury E et al. *JAMA Network Open.*

Name of Study	Trial or Registration Number	Location	Number of patients and patient characteristics	Primary endpoint
Outcome of cancer patients infected with COVID-19, including toxicity of cancer treatments	-	France	7,251 patients	Clinical deterioration, defined as the need for O2 supplementation of 6l/min or more, or death of any cause.
Incidence of thrombosis and hemorrhage in hospitalized cancer patients with COVID-19	-	USA	45 cancer patients and 353 non cancer patients	Evaluate cumulative incidences of thrombotic and hemorrhagic events in hospitalized COVID-19 patients with and without active cancer at 28 days.
Poor outcome and prolonged persistence of SARS-CoV-2 RNA in COVID-19 patients with haematological malignancies; King's College Hospital experience	-	UK	80 patients	Comparison of first 80 patients with haematological malignancy with all other patients admitted to our hospital with COVID-19 in the same time frame to define relative risk and identify factors that increase mortality within this subgroup.
Nosocomial outbreak of SARS-CoV-2 infection in a haematological unit – High mortality rate in infected patients with haematologic malignancies	-	Poland	19 patients with haematological malignancies and 20 health care workers	Compare morbidity and mortality in infected and non-infected patients after exposure to SARS-CoV-2.
COVID-19 infection in hematopoietic cell transplantation: age, time from transplant and steroids matter	-	USA	34 haematopoietic stem cell transplant recipients	To identify mortality risk to HCT recipients
COVID-19 in patients with hematological malignancies: A retrospective case series	-	Spain	41 patients with haematological malignancies	Characterize the real impact of COVID-19 in patients with hematological neoplasms, in order to optimize clinical decision-making.
Clinical characteristics and outcome of multiple myeloma patients with concomitant COVID-19 at Comprehensive Cancer Centers in Germany	-	Germany	21 patients	Characterize a population of MM patients registered from 10 institutions who developed COVID-19 at hotspot areas in Germany
COVID-19, impact on myeloma patients	-	Belgium	20 patients with multiple myeloma	Assess the impact of COVID-19 in the Belgian MM community,

© 2022 Khoury E et al. *JAMA Network Open.*

Name of Study	Trial or Registration Number	Location	Number of patients and patient characteristics	Primary endpoint
**Psychological Impact:**				
COVID-19 Pandemic Impact on Patients With Cancer - a Danish Survey (COPICADS)	NCT04389996	Denmark	5000 patients	Overall Quality of Life
Impact of the COVID-19 Pandemic and HRQOL in Cancer Patients and Survivors	NCT04447222	USA	1242 patients	Coronavirus disease-2019 (COVID19)-specific psychological distress
Impact of COVID-19 on Lung Cancer Patients	NCT04538456	UK	800 patients with lung cancer	Physical, social impact and psychological impact
Perception of the COVID-19 Pandemic in Patients With Haematological or Solid Neoplasias	NCT04649320	Austria	300 patients with malignant disease (solid or haematological)	Influence of the COVID-19 pandemic on cancer patient's daily life
The effects of prevention and control measures on treatment and psychological status of cancer patients during the novel coronavirus pneumonia (COVID-19) outbreak	ChiCTR2000030686	China	300 patients with malignant disease	The effects of prevention and control measures on treatment and psychological status of cancer patients during the novel coronavirus pneumonia (COVID-19) outbreak http://www.chictr.org.cn/showproj.aspx?proj=50714

eTable 14 Table of completed and ongoing cancer observational studies related to the SARS-CoV-2/COVID-19 pandemic in patients with malignant disease.
AML, acute myeloid leukaemia.

© 2022 Khoury E et al. *JAMA Network Open.*

eFigure 20. Forest Plot of Relative Risk of Mortality in Different Cancer Types in the Subgroup Analysis

A, Breast cancer and COVID-19, compared to patients with non-breast cancers and COVID-19. B, Gynaecological cancer and COVID-19, compared to patients with non-gynecological cancers and COVID-19. C, Patients with lung cancer and COVID-19 compared to patients with non-lung cancers and COVID-19. D, Patients with genitourinary cancer and COVID-19 compared to patients with non-genitourinary cancers and COVID-19. E, Patients with hematological malignancies and COVID-19 compared to patients with non-hematological malignancies and COVID-19.

A

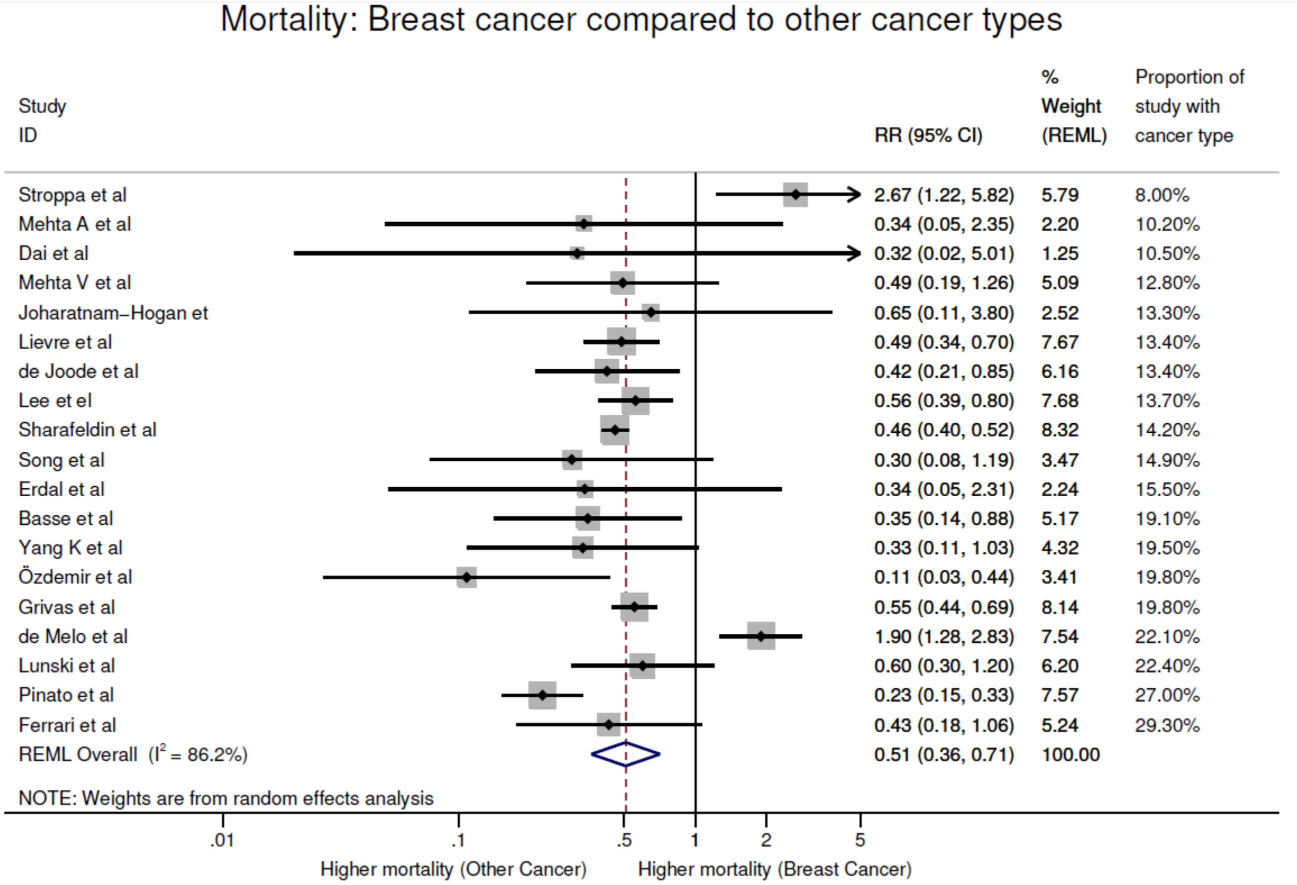

© 2022 Khoury E et al. *JAMA Network Open.*

B

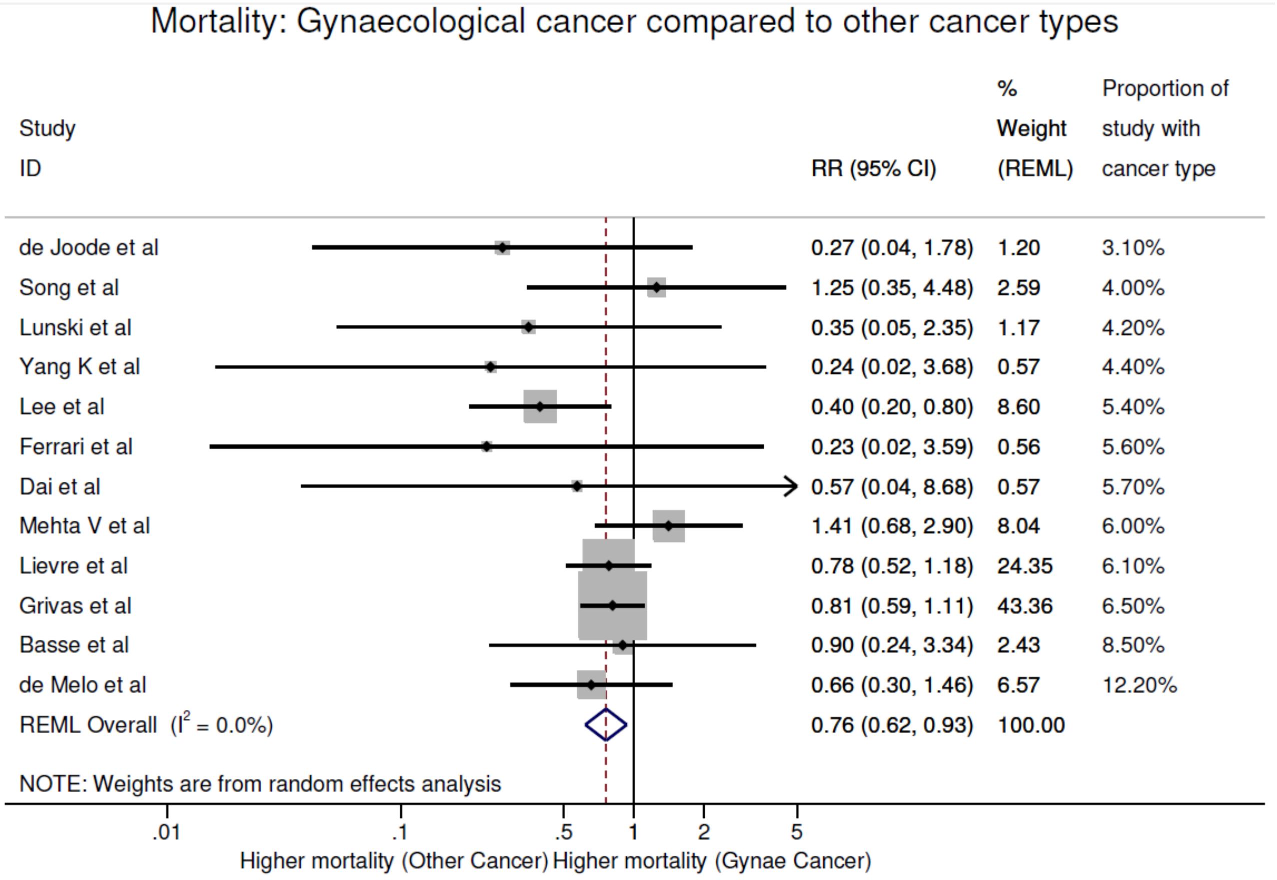

© 2022 Khoury E et al. *JAMA Network Open*.

C

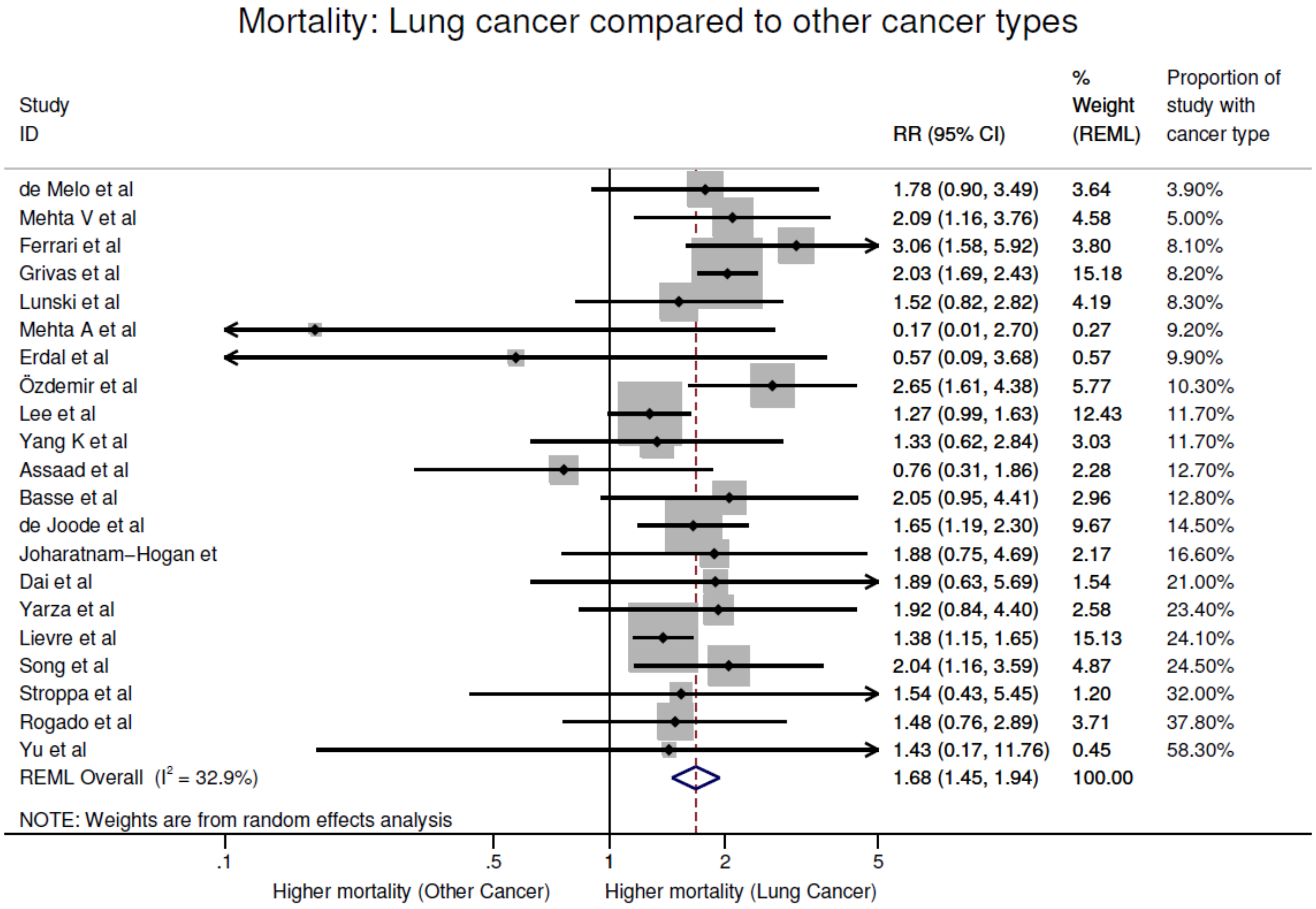

© 2022 Khoury E et al. *JAMA Network Open*.

D

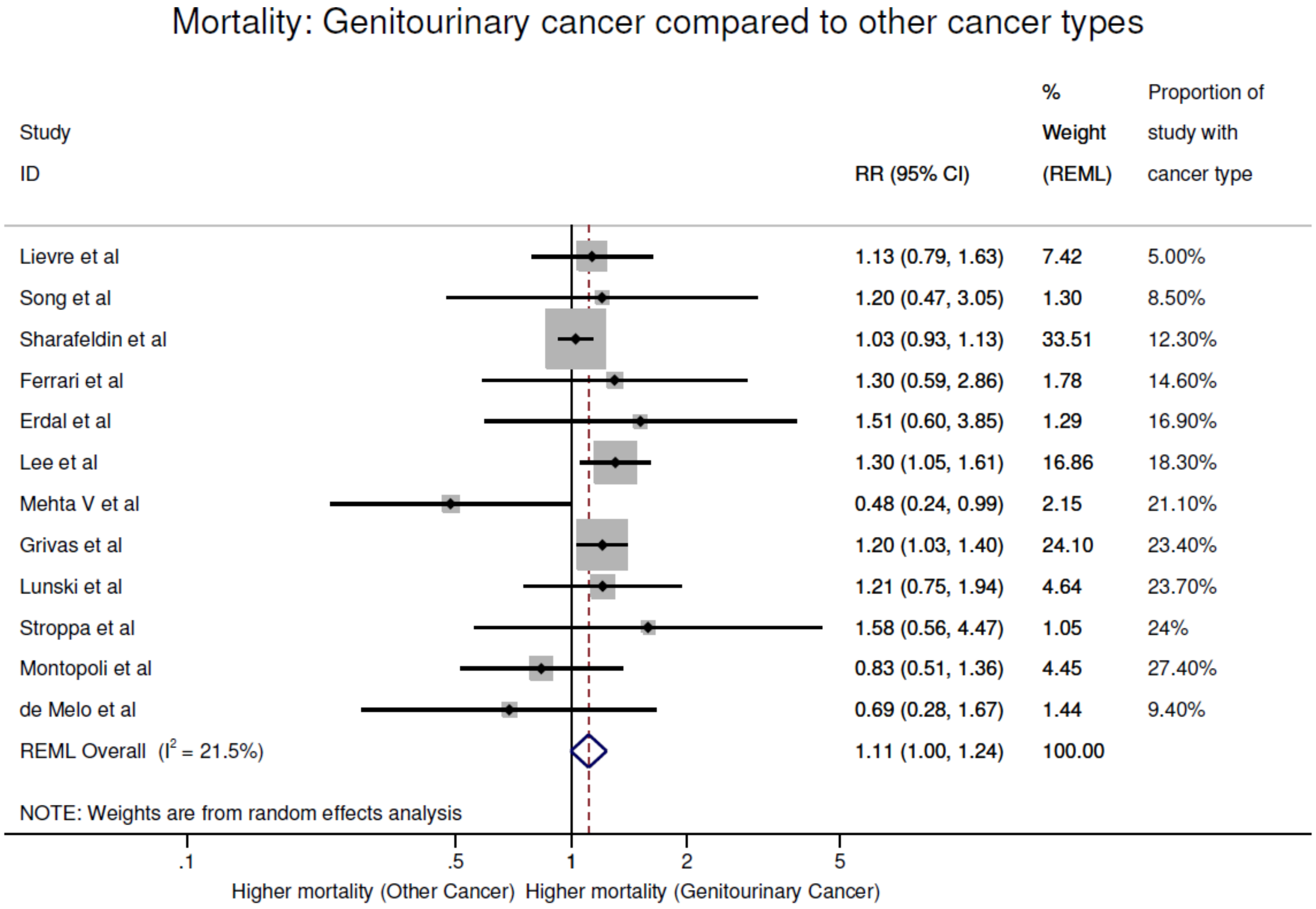

© 2022 Khoury E et al. *JAMA Network Open.*

E

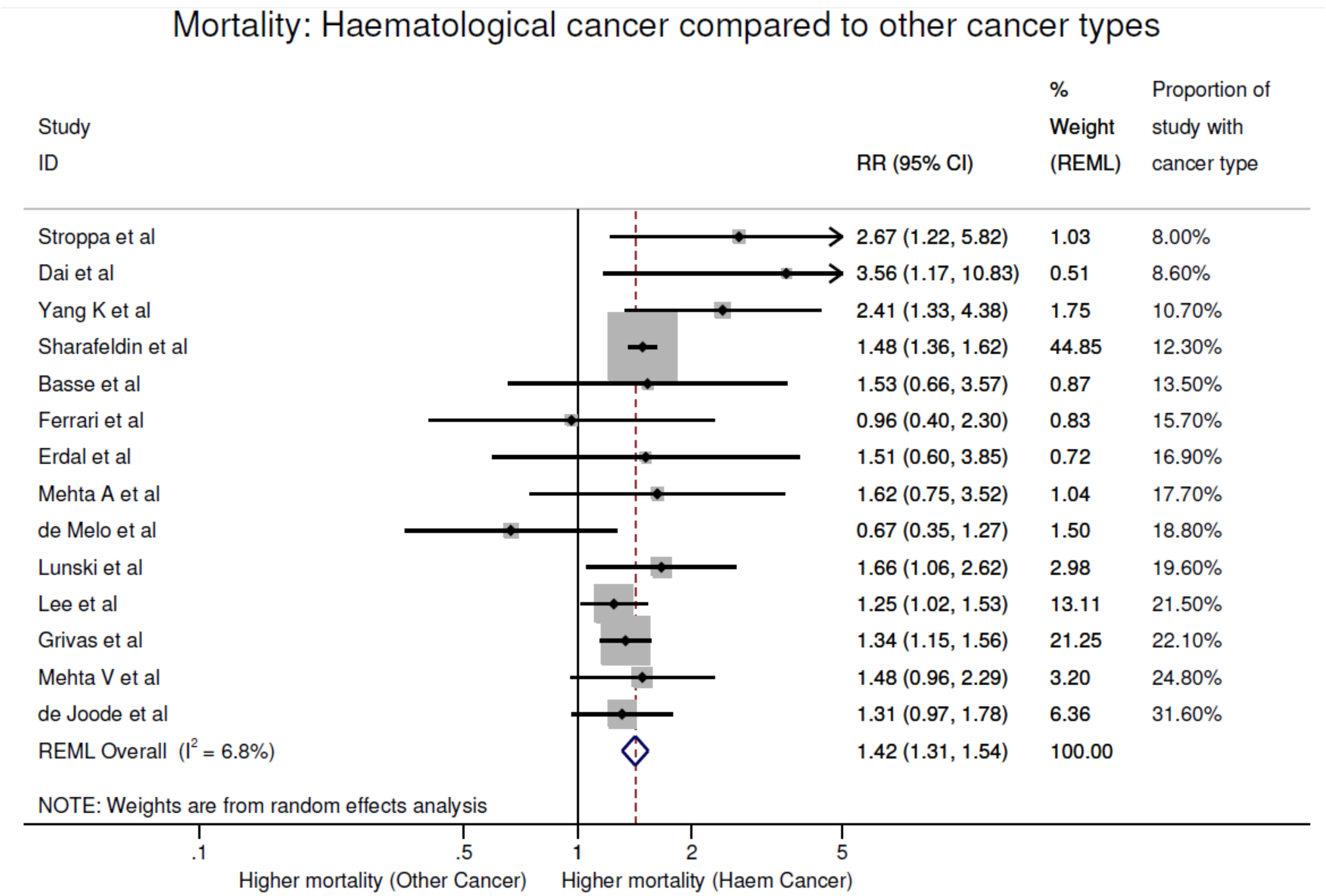

© 2022 Khoury E et al. *JAMA Network Open*.

eFigure 20. Continued

A) Patients with gastrointestinal cancer and COVID-19, compared to patients with non-gastrointestinal cancers and COVID-19. B) Patients with skin cancer and COVID-19, compared to patients with non-skin cancers and COVID-19.

A

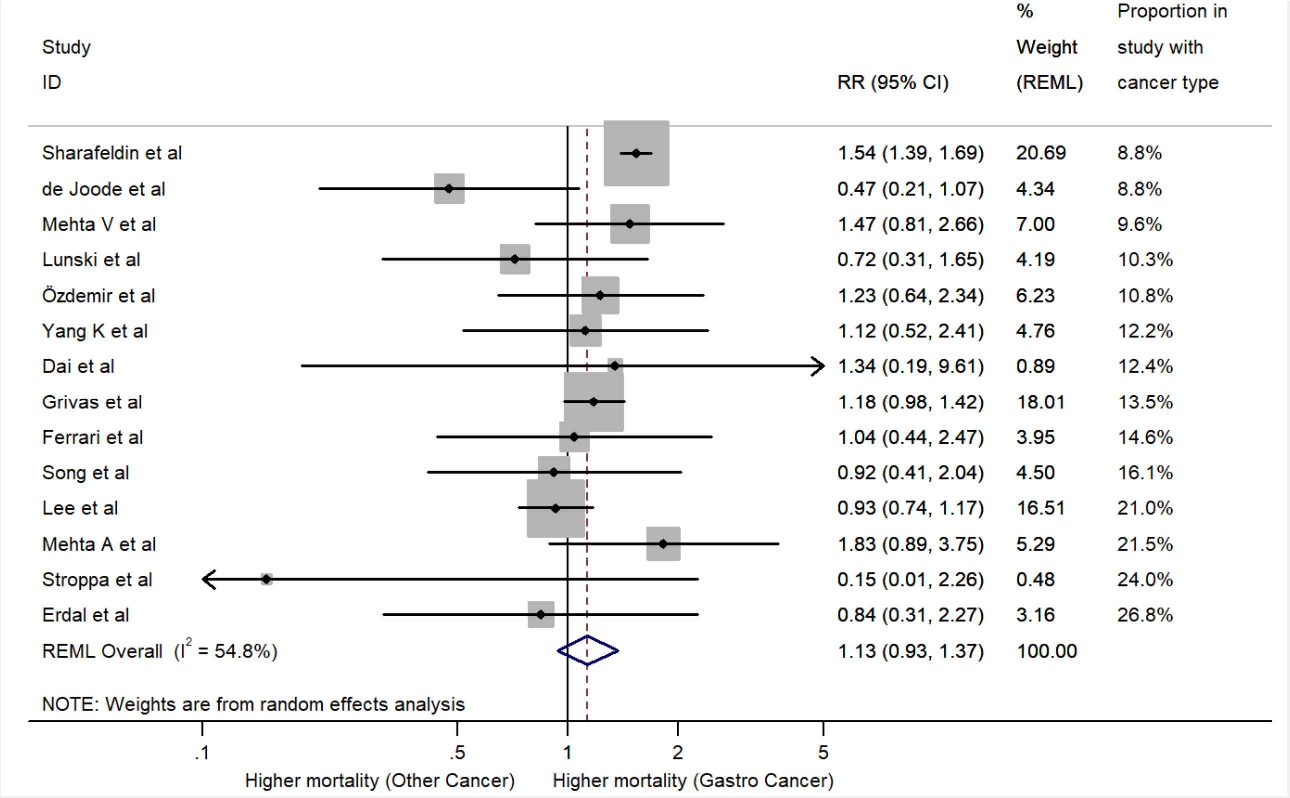

© 2022 Khoury E et al. *JAMA Network Open.*

B

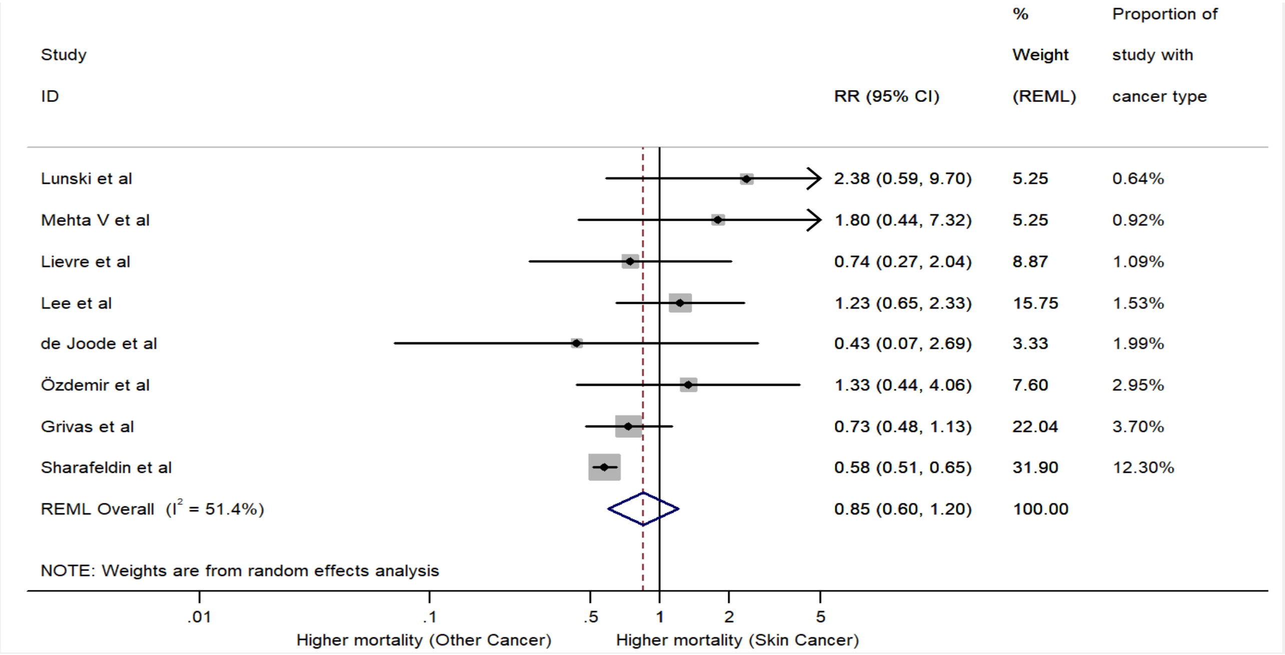

© 2022 Khoury E et al. *JAMA Network Open*.
